# The role of price and convenience in use of oral rehydration salts to treat child diarrhea: A cluster randomized trial in Uganda

**DOI:** 10.1371/journal.pmed.1002734

**Published:** 2019-01-24

**Authors:** Zachary Wagner, John Bosco Asiimwe, William H. Dow, David I. Levine

**Affiliations:** 1 Department of Economics, Sociology and Statistics, RAND Corporation, Santa Monica, California, United States of America; 2 School of Planning and Statistics, Makerere University, Kampala, Uganda; 3 School of Public Health, University of California at Berkeley, Berkeley, California, United States of America; 4 Haas School of Business, University of California at Berkeley, Berkeley, California, United States of America; Makerere University Medical School, UGANDA

## Abstract

**Background:**

Over half a million children die each year of diarrheal illness, although nearly all deaths could be prevented with oral rehydration salts (ORS). The literature on ORS documents both impressive health benefits and persistent underuse. At the same time, little is known about why ORS is underused and what can be done to increase use. We hypothesized that price and inconvenience are important barriers to ORS use and tested whether eliminating financial and access constraints increases ORS coverage.

**Methods and findings:**

In July of 2016, we recruited 118 community health workers (CHWs; representing 10,384 households) in Central and Eastern Uganda to participate in the study. Study villages were predominantly peri-urban, and most caretakers had no more than primary school education. In March of 2017, we randomized CHWs to one of four methods of ORS distribution: (1) free delivery of ORS prior to illness (free and convenient); (2) home sales of ORS prior to illness (convenient only); (3) free ORS upon retrieval using voucher (free only); and (4) status quo CHW distribution, where ORS is sold and not delivered (control). CHWs offered zinc supplements in addition to ORS in all treatment arms (free in groups 1 and 3 and for sale in group 2), following international treatment guidelines. We used household surveys to measure ORS (primary outcome) and ORS + zinc use 4 weeks after the interventions began (between April and May 2017). We assessed impact using an intention-to-treat (ITT) framework. During follow-up, we identified 2,363 child cases of diarrhea within 4 weeks of the survey (584 in free and convenient [25.6% of households], 527 in convenient only [26.1% of households], 648 in free only [26.8% of households], and 597 in control [28.5% of households]). The share of cases treated with ORS was 77% (448/584) in the free and convenient group, 64% (340/527) in the convenient only group, 74% (447/648) in the free only group, and 56% (335/597) in the control group. After adjusting for potential confounders, instructing CHWs to provide free and convenient distribution increased ORS coverage by 19 percentage points relative to the control group (95% CI 13–26; *P <* 0.001), 12 percentage points relative to convenient only (95% CI 6–18; *P <* 0.001), and 2 percentage points (not significant) relative to free only (95% CI −4 to 8; *P* = 0.38). Effect sizes were similar, but more pronounced, for the use of both ORS and zinc. Limitations include short follow-up period, self-reported outcomes, and limited generalizability.

**Conclusions:**

Most caretakers of children with diarrhea in low-income countries seek care in the private sector where they are required to pay for ORS. However, our results suggest that price is an important barrier to ORS use and that switching to free distribution by CHWs substantially increases ORS coverage. Switching to free distribution is low-cost, easily scalable, and could substantially reduce child mortality. Convenience was not important in this context.

**Trial registration:**

Trial registry number AEARCTR-0001288.

## Abstract 2

Zachary Wagner and colleagues reveal an increased use of rehydration salts in children to prevent diarrhoea when freely distributed in a randomised trial in Uganda.

## Author summary

**Why was this study done?:**

- A large body of evidence documents that oral rehydration salts (ORS) are extremely effective at preventing child mortality from diarrhea.
- At the same time, a large evidence base documents persistent underuse of ORS around the globe.
- The existing literature provides limited insight into why ORS continues to go underused and what interventions could effectively increase ORS coverage.

**What did the researchers do and find?:**

- We hypothesized that price and inconvenience are important barriers to ORS use.
- We tested this hypothesis by experimentally varying ORS price and convenience using a cluster-randomized controlled trial (RCT) with a factorial design.
- We demonstrate that making ORS free and convenient through a novel home delivery intervention carried out by community health workers (CHWs) increased ORS coverage from 56% to 77%.
- Moreover, we use our factorial design to show that price was an important barrier to ORS use, whereas convenience was not important in our setting.

**What do these findings mean?:**

- Although most caretakers in low-income countries seek care in the private sector, where they are required to pay for ORS, our findings suggest that prices create an important barrier to use.
- Having CHWs switch from charging to free ORS distribution is low cost, easily scalable, and could substantially reduce child mortality.

## Introduction

Diarrhea continues to kill over half a million children each year, almost all in poor nations [1]. Fortunately, use of oral rehydration salts (ORS) could avert nearly all of these deaths [2–6]. Thus, in 1978, *The Lancet* lauded ORS as “potentially the most important medical advance of this century” [7].

Despite well documented effectiveness in preventing death from diarrhea, most diarrhea cases in low-income countries still are not treated with ORS [8–13]. In Uganda, the location of this study, only 46% of diarrhea cases are treated with ORS [14]. More recently, zinc was added to diarrhea treatment guidelines (in combination with ORS), after zinc was demonstrated to reduce illness severity and prevent recurrence [15–17]. However, only 40% of cases in Uganda receive zinc [14].

At the same time, little is known about why use remains low and what can be done to increase use [18]. Two potentially important barriers to ORS + zinc use are the price and the inconvenience of accessing these products. Many studies highlight price as a key barrier to take-up of several other health products [19–22]. Although ORS + zinc is free at public health clinics, most caretakers seek care in the private sector where they are required to pay [14]. Similarly, several studies find inconvenience of access reduces health product take-up [19,20]. Convenience may be especially important for treating child diarrhea because most children have several cases per year, requiring caretakers to make this inconvenient trip repeatedly [14].

In this study, we used a four-armed randomized controlled trial (RCT) to evaluate the impact of several novel community health worker (CHW)-based interventions aimed at eliminating financial and access constraints to ORS + zinc use (ORS use is our primary outcome, but the interventions included both ORS and zinc). CHWs are increasingly utilized to expand access to health products in low-income countries and are a promising mechanism for improving ORS + zinc coverage. A novel preemptive delivery intervention made ORS + zinc free and conveniently available inside the home before a child came down with diarrhea (free and convenient). A preemptive home sales intervention made accessing ORS + zinc convenient, but not free (convenient only). A “free upon retrieval” intervention made ORS + zinc free, but not as convenient as home delivery (free only). Finally, a control group had CHWs carry out their normal activities (ORS + zinc were not free and usually not delivered). This design compares the effectiveness of competing CHW distribution strategies and sheds light on reasons for underuse of ORS + zinc.

## Methods

### Trial design

This study used a cluster randomized factorial design in Mukono, Jinja, and Buikwe districts in Central and Eastern Uganda. We chose a cluster randomized design because the interventions were administered by CHWs, each of whom covers one village. We allocated equal shares of CHWs (and villages) to each of the four study arms.

### Participants

Community Health Promoters, a CHW program implemented by BRAC Uganda, carried out the intervention. BRAC hires community members to act as health workers in their village. Our interventions were designed to change the way that BRAC’s CHWs distribute ORS + zinc. Under the status quo (our control group), CHWs sell ORS + zinc in addition to an array of health products, such as chlorine, bed nets, malaria treatment, and other basic household items (e.g., soap). CHWs purchase products from BRAC at a subsidized price and sell them to community members for a profit (usually at the market price). BRAC also trains CHWs to provide very basic primary care and health education, but CHWs do not have any formal medical training. BRAC instructs CHWs to visit each household once per month. In practice, most CHWs visit only a minority of homes each month [23]. Promotion of ORS + zinc is a key aspect of BRAC’s CHW program. The market price for a sachet of ORS is 500 Ugandan Shillings (about US$0.15), while a strip of 10 zinc tablets costs 1,000 Ugandan shillings (about US$0.30).

In July 2016, we worked with BRAC to recruit 118 CHWs (in 118 villages) to participate in the study. We selected villages to minimize travel costs, so our sample is not representative of all of Uganda. All villages were within a 3-hour drive from Kampala, Uganda’s capital city, and most villages were peri-urban (S1 Appendix Fig A has a map of the study area and more details on the setting).

CHWs are responsible for roughly 88 households with a child under 5 years old (“under 5”). We estimate that the total population of these villages was 10,384 households with a child under 5.

At the household level, study participants were sampled caretakers of children under 5 who lived in enrolled villages.

### Interventions

We randomly assigned each CHW/village to one of four groups.

#### 1. Free and preemptive home delivery (free and convenient)

We instructed CHWs to visit all of the households in their catchment area with a child under-five at the beginning of the study and give caretakers two free packets of ORS and ten free tablets of zinc per child under-five (the recommended quantity for one case of diarrhea) to store in their homes. In addition, we asked CHWs to provide BRAC’s standard information on ORS + zinc (see S1 Appendix Fig B which is translated from Luganda).

#### 2. Preemptive home sales (convenient only)

We instructed CHWs to visit all of the households in their catchment area with a child under five at the beginning of the study and offer to sell ORS + zinc to caretakers at the market price (US$0.15 per ORS packet and US$0.30 for 10 tablets of zinc, about 13% of the average daily household income in Uganda [24]). CHWs retained the revenue from any sales. We also asked CHWs to provide the standard information on ORS + zinc. This intervention is nearly identical to how BRAC designed their CHW program; however, in practice, most CHWs do not make these household visits.

#### 3. Free upon retrieval (free only)

We instructed CHWs to visit all of the households in their catchment area that contained a child under five years old at the beginning of the study and provide caretakers with one voucher per child under five that they could redeem at the CHW’s home for two packets of ORS and 10 tablets of zinc. We also asked CHWs to provide the standard information on ORS + zinc. On average, it takes about 10 minutes for caretakers to walk to the CHW’s home.

#### 4. Control

No intervention took place, and CHWs carried out their normal activities. Caretakers in these villages had standard access to ORS + zinc at local health facilities and pharmacies. At baseline, most caretakers obtained ORS from a private seller (43%), CHW (33%), or a public clinic (20%). Almost all caretakers retrieved ORS after the child came down with diarrhea. Although ORS is free at public health facilities, over 70% of caretakers that used ORS at baseline paid for it.

We asked all CHWs to make one visit per household at the beginning of the study. The only difference between free and convenient distribution and convenient only distribution is the price charged by the CHW. Therefore, comparing these groups examines the role of ORS price (and its effect on both CHW effort and household demand) while holding other factors constant. Similarly, the only difference between free and convenient distribution and free only distribution is whether the CHW delivers the ORS + zinc ahead of illness or whether someone must retrieve the products. Therefore, comparing these groups examines the role of convenience (Table 1).

**Table 1:** Mechanisms of interventions.

	Preemptive home access	Free distribution	Increased supply	Increased information
**Control**				
**Free and convenient**	X	X	X	X
**Convenient only**	X		X	X
**Free only**		X	X	X

Households assigned to the free and convenient arm were also informed that they would receive a small incentive (about US$0.30) if they retained the packaging of the treatment that was delivered, whether or not the treatment was used. Observation of packaging was then used as an objective measure to validate self-reported ORS usage. It was not feasible to incentivize retention of packaging in the other three groups, as that would have incentivized acquisition of new ORS packets. See S1 Appendix Section S3 for more details on how the field team implemented the incentives for package retention and for evidence on why this does not bias self-reported estimates.

### Randomization

The authors conducted randomization stratified by six BRAC branches, which are local offices responsible for coordinating CHW activities (19–20 CHWs per branch), and baseline ORS use. We used the “randtreat” package in StataSE version 14.2 (StataCorp, College Station, TX) to randomize, which bins villages in each of the different strata and randomly assigns to a study arm within strata using a random number generator. Masking of participants was not feasible; data analysts were not masked either, but analysis followed a detailed pre-analysis plan.

### Training CHWs to carry out interventions

We asked CHWs that were assigned to one of the treatment groups to attend a 1-hour training session at the local BRAC office. We conducted the trainings for the three different interventions separately and asked trainees not to discuss the training with other CHWs. Trainings were identical across treatment groups aside from instructions on distributing ORS + zinc. See S1 Appendix Section S1 for full instructions.

After the training, we gave all trainees a box filled with two ORS packets and 10 zinc tablets per child under 5 in their catchment area. CHWs were responsible for an average of 88 households with a child under 5, and we provided each CHW with an average of 290 packets of ORS and 145 strips of zinc (10 tablets per strip). We provided the same quantity of ORS + zinc regardless of intervention group assignment.

CHWs agreed to make these deliveries or sales visits within 3 days of the training. We paid CHWs roughly US$6 at the training and said we would pay them an additional US$6 after they carried out the interventions. Although CHWs may have perceived this payment as performance-linked, we ultimately provided the full payment to all CHWs that we trained. Payment instructions and distribution was identical for each treatment arm.

### Sampling and data collection

After enrollment but prior to random assignment, CHWs listed all households in their catchment area with a child under 5, which was used as a sampling frame. After the village listing, we conducted a baseline survey (July 20, 2016 to August 3, 2016), in which we visited the 40 households on the list who lived closest to the CHW’s home. We used the baseline sample for stratified randomization (described above) to assess village-level balance between study groups on important baseline characteristics (e.g., ORS use) and to control for potential pre-intervention village-level characteristics. Therefore, baseline sample size is less important for statistical power in our endline analysis, and we chose a larger endline sample (80 households per village) to maximize statistical power.

One month after the interventions were implemented, we conducted an endline survey, in which we visited the 80 closest households to the CHW’s home on the list. If a village had fewer than 80 households with an under-5 child (roughly 50%), we visited all homes on the list. It is important to note that we instructed CHWs to carry out the intervention for all of the households in their catchment area, not just the 80 we surveyed. They also did not know which households we would survey. We ended up sampling 75% of the household in the sampling frame. A 1-month follow-up period was a sufficient duration for roughly one-third of the households to have a case of diarrhea, which is what we used to estimate statistical power. Caretakers gave oral informed consent to participate.

At baseline and endline, caretakers who reported a diarrhea case within the last 4 weeks were asked a series of question about case management as well as about prior diarrhea treatment behavior, knowledge, and characteristics about the household, caretaker, and child. Caretakers who did not report a recent case completed only a short survey asking about take-up of ORS + zinc and contact with the CHW. The main analysis includes only caretakers who cared for a child with a recent case of diarrhea.

### Outcomes

Because some caretakers cared for multiple children with diarrhea, we assessed outcomes at the diarrhea case level. Our prespecified primary outcome was caretaker-reported ORS use for a case of child (under-5) diarrhea that occurred within the last 4 weeks. We measured ORS use through a series of questions about whether the caretaker used ORS to treat the recent case. Each case was coded as 1 if the caretaker reported using any ORS and 0 otherwise.

We also examined several secondary treatment outcomes. We examined zinc use in combination with ORS, which is the USAID-/WHO-/UNICEF-recommended treatment [17]. We examined the time between diarrhea onset and ORS use. Finally, we examined use of antibiotics, which are typically unnecessary for child diarrhea. Thus, use of antibiotics largely contributes to antibiotic resistance without benefiting the child. At the request of an anonymous reviewer, we also added an ex post outcome of any use of zinc.

### Sample size

We powered the study to detect (at the 95% level) an 11- to 14-percentage point difference (depending on assumptions about intraclass correlation) in ORS use between treatment arms with power of 0.8.

### Statistical analysis

We used an intention-to-treat (ITT) framework for our main analyses, which tested three hypotheses: (1) making ORS free and convenient increases ORS use relative to a control group, (2) making ORS free and convenient increases ORS use more than making it convenient only (role of price), and (3) making ORS free and convenient increases ORS use more than making it free only (role of convenience). Although some CHWs did not carry out the intervention properly, we included all CHWs and all households in an ITT analysis to preserve the unbiasedness benefits of randomization. Thus, our estimated effects capture how each intervention affected the behavior of both CHWs and households.

We used logistic regressions to examine the impact of the interventions. For diarrhea treatment outcomes, we used an unadjusted model and an adjusted model that included several pre-specified control variables to correct for potential imbalance and improve the precision of our estimates. Prespecified control variables included branch fixed-effects, caretaker characteristics (age, education, number of children), child characteristics (age, diarrhea frequency, blood in stool, concurrent fever), household characteristics (water source, latrine type, main source of income), and baseline village characteristics (percentage of households using respective treatment, percentage of households visited by CHW in past month, percentage of households aware of free ORS in village, percentage of households with ORS stored in their home).

We clustered standard errors at the village level (using Stata’s -cluster- command) and analyzed data at the case level [25,26]. All analyses were carried out in StataSE version 14.2. See S1 Appendix section S2.1 for more details on the regression specifications.

For secondary treatment outcomes, we adjusted *P* values for multiple hypothesis testing using the free step-down resampling method to control the false discovery rate (see section S6 of the S1 Appendix for details) [27].

A study protocol and pre-analysis plan are available at the AEA RCT Registry (registry number AEARCTR-0001288). There were no adverse events reported.

### Ethics approval

We obtained ethics approval from the UC Berkeley Committee for Protection of Human Subjects, Mildmay Uganda Research Ethics Committee, and Uganda National Council for Science and Technology.

## Results

**Fig 1:**
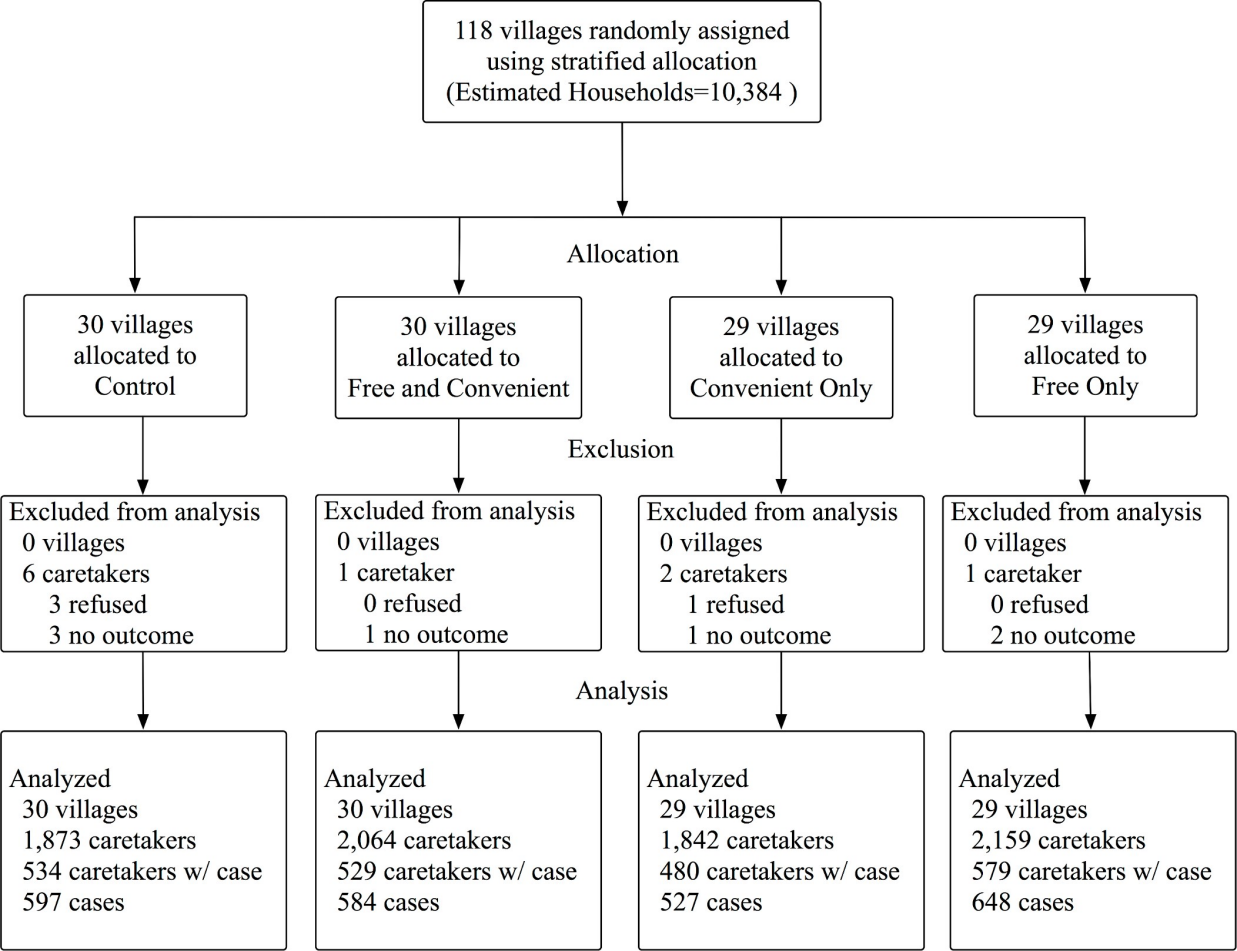
Randomization diagram. Number of villages is equivalent to number of CHWs. “Caretakers” include the sample that completed the endline survey. “Caretakers w/ case” include the sample that completed the endline survey and cared for a case of diarrhea in the last 4 weeks. “No outcome” implies that caretakers did not know whether the case was treated or what treatment was used. “Estimated households” only includes households with a child under 5 and assumes 88 households with a child under 5 per village (the average in our sample).

We recruited 4,150 caretakers to participate in the baseline survey in July 2016. We randomized and trained CHWs to carry out the interventions in March of 2017 and followed up with a household survey of 7,949 caretakers in April and May of 2017 (4 weeks after the interventions started). Random assignment resulted in 30 CHWs in the free and convenient arm, 29 CHWs in the convenient only arm, 29 CHWs in the free only arm, and 30 CHWs in the control arm (see Fig 1). Of the 88 CHWs invited to a training session, 86 attended. In two villages assigned to free and convenient distribution, the CHW quit after random assignment and BRAC did not hire a replacement. Therefore, the intervention was not carried out in these villages. However, we still included these villages in the ITT analysis.

We identified 1,770 cases of diarrhea at baseline (33% of caretakers reported a case) and 2,363 cases at endline (27% of caretakers reported a case). Four caretakers refused to participate in the survey, and we excluded seven cases because the caretaker did not know how the diarrhea was treated. We analyzed 2,356 endline cases of diarrhea: 584 in free and convenient (25.6% of households), 527 in convenient only (26.1% of households), 648 in free only (26.8% of households), and 597 in control (28.5% of households).

Table 2 displays characteristics of the sample and baseline diarrhea treatment characteristics of the villages. Characteristics, including ORS coverage, were similar between groups at baseline. However, a multinomial logit regressing treatment assignment on all covariates produced a chi-squared test statistic with a *P* value of less than 0.01 (S1 Appendix section S2.2). This result suggests that covariates are jointly predictive of treatment assignment, which is indicative of imbalance and provides motivation for including controls.

**Table 2:** Sample characteristics and baseline diarrhea case management.

	Control	Free and convenient	Convenient only	Free only
**Households with case of diarrhea**	28.5%	25.6%	26.1%	26.8%
**Caretaker characteristics**				
Age	28.5	30.1	28.3	29.7
Number of children	2.9	3.1	3.0	3.1
Highest education				
*None*	8.0%	11.8%	5.5%	9.4%
*Primary*	54.2%	45.6%	46.5%	48.4%
*Secondary+*	37.8%	42.6%	48.0%	42.2%
**Child characteristics**				
Child age (months)	22.9	23.9	22.2	24.3
Male	53.8%	54.4%	52.9%	53.3%
**Diarrhea case characteristics**				
Blood in stool	7.0%	5.8%	10.6%	6.2%
Concurrent fever	55.3%	51.3%	56.3%	57.3%
**Water source**				
Piped	14.2%	18.5%	23.8%	27.1%
Protected well	69.3%	68.4%	57.8%	56.1%
Unprotected source	13.3%	9.6%	12.9%	13.4%
**Baseline diarrhea treatment characteristics of village**^**a**^				
Percentage of cases used ORS (last 4 weeks)	60.9%	62.2%	58.6%	59.1%
Percentage of cases used ORS + zinc (last 4 weeks)	35.2%	35.6%	29.9%	36.9%
Percentage heard of ORS	96.6%	94.6%	95.6%	98.5%
Percentage aware of free ORS in village	27.4%	25.5%	38.0%	33.2%

^a^Baseline diarrhea treatment characteristics were collapsed to the village level and merged with endline data.

Unit of observation = case of diarrhea. Multinomial logit regressing treatment assignment on all covariates in the table produces chi-squared test statistic with *P <* 0.01, which rejects perfect randomization.

**Abbreviation:** ORS, oral rehydration salts.

**Fig 2:**
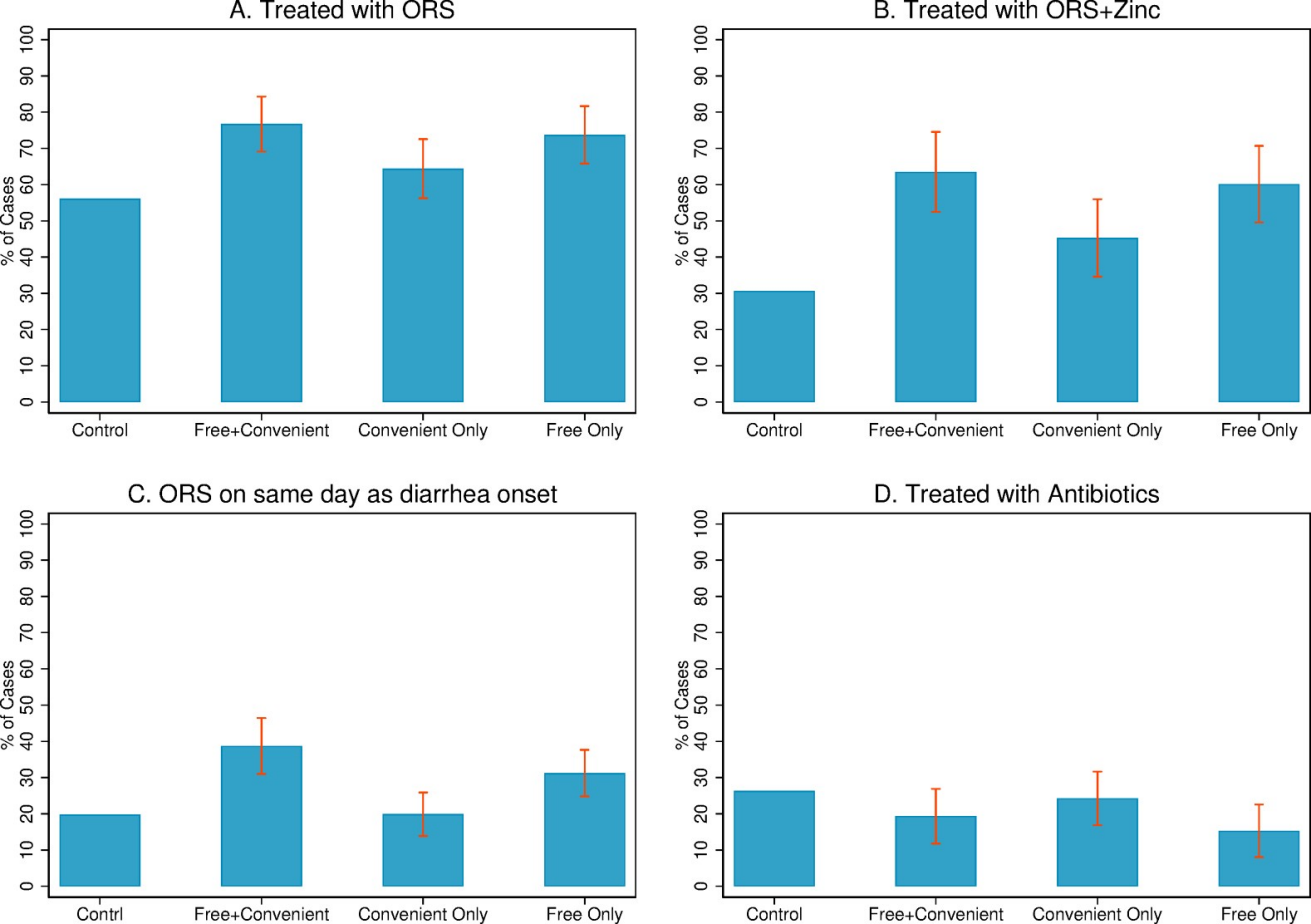
Unadjusted diarrhea treatment outcomes. Exposure to interventions was incomplete (60% in free and convenient; 19% in convenient only; 42% in free only). Estimates are ITT, and the sample includes all households with a case of diarrhea in 4 weeks leading up to the interview (control = 597; free and convenient = 584; convenient only = 527; free only = 648). The confidence intervals were estimated using logistic regressions with standard errors clustered by village, calculated using the Delta method [28]. All were outcomes prespecified. ITT, intention to treat; ORS, oral rehydration salts.

Fig 2 presents unadjusted diarrhea treatment outcomes for cases that occurred in the prior 4 weeks to the endline survey. ORS coverage was 76.7% (448/584) in the free and convenient group, 64.3% (340/528) in the convenient only group, 73.7% (477/647) in the free only group, and 56.1% (335/597) of cases in the control group (see S1 Appendix Table F for unadjusted means).

**Fig 3:**
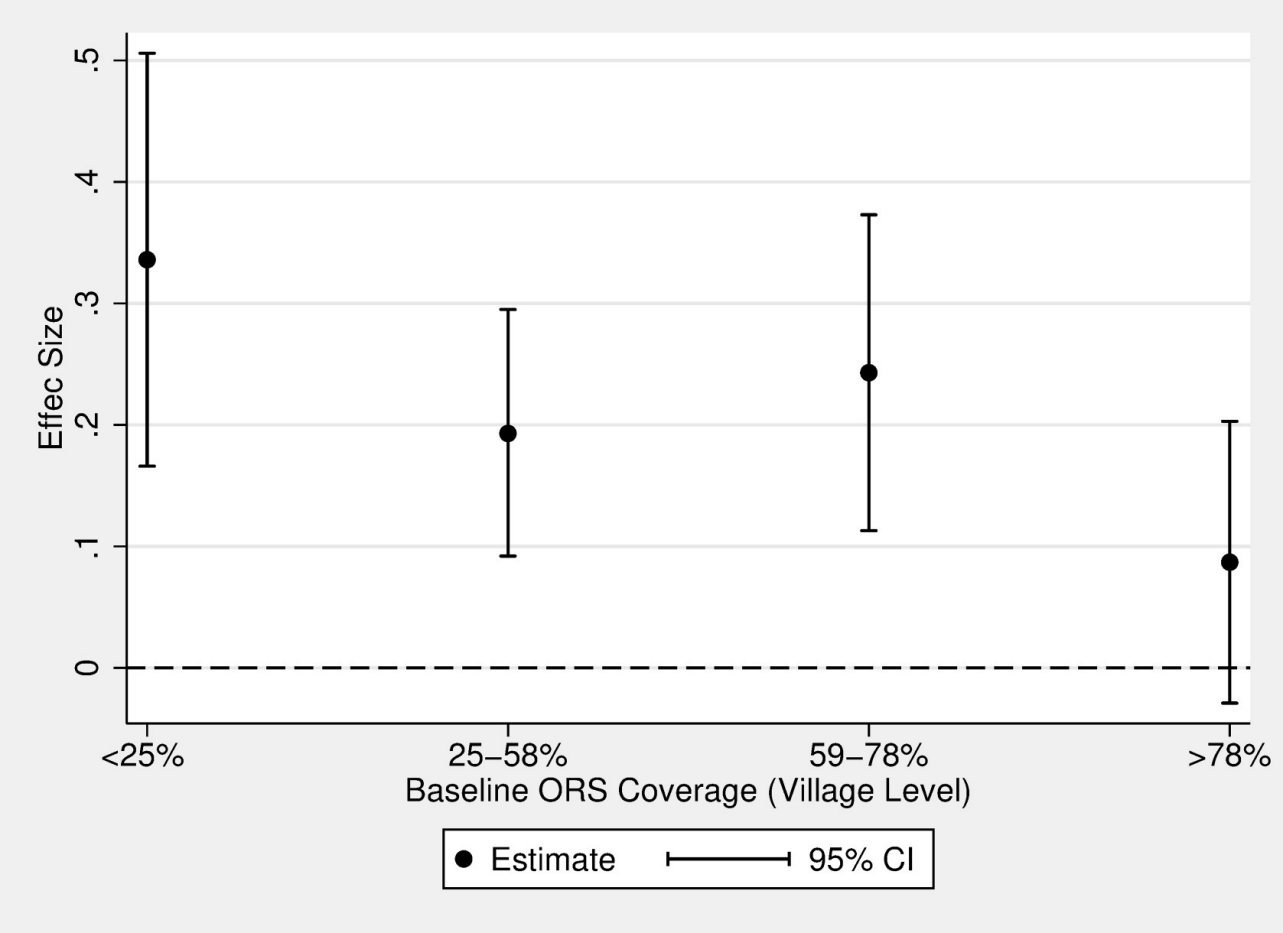
Endline ORS use by baseline village-level ORS use. This figure presents the effect size of the free + convenient intervention relative to the control group at different levels of baseline ORS coverage. The x-axis represents the cut points of the quartiles of baseline village-level ORS coverage; quartile 1 = 11%–24%; quartile 2 = 25%–58%; quartile 3 = 59%–78%; and quartile 4 = 79%–100%. The y-axis represents endline ORS coverage. This figure demonstrates that the intervention had a stronger effect among villages with particularly low ORS use at baseline. Interaction models show that the treatment effect is statistically different between <25% and >78% (interaction term = 0.249; *P* = 0.018) and between 59%–78% and >78% (interaction term = 0.156; *P* = 0.079). ORS, oral rehydration salts.

Table 3 presents unadjusted and adjusted effect sizes of free and convenient distribution relative to the other groups. *P* values for secondary outcomes—used ORS on same day as diarrhea began, used ORS + zinc, and used antibiotics (family size of three)—were adjusted for multiple hypotheses as describe above. We present estimates as the impact of free and convenient relative to the other treatment arms because our primary research questions all involve this comparison (i.e., impact of free and convenient distribution, role of price, and role of convenience). After adjusting for covariates, the free and convenient distribution arm had 19 percentage points higher ORS coverage than the control group (95% CI 13–26; *P <* 0.001), 12 percentage points higher than the convenient only arm (the role of price; 95% CI 6–18; *P <* 0.001), and 2 percentage points higher (not significant) than the free only arm (the role of convenience; 95% CI −4 to 8; *P* = 0.38). The increase in ORS usage for the free and convenient distribution arm was largest in villages that had lower ORS coverage prior to the intervention (Fig 3).

**Table 3:** Effect sizes from logistic regressions: Free and convenient distribution compared to other arms.

	Free and Convenient Versus Control		Free and Convenient Versus Convenient Only (role of price)		Free and Convenient Versus Free Only (role of convenience)	
	Unadjusted	Adjusted	Unadjusted	Adjusted	Unadjusted	Adjusted
**Primary Outcome**						
Used ORS (primary outcome)	0.21	0.19	0.12	0.12	0.03	0.02
	(0.13 to 0.28)	(0.13 to 0.26)	(0.05 to 0.19)	(0.06 to 0.18)	(−0.04 to 0.10)	(−0.04 to 0.08)
**Secondary Outcomes**						
Used ORS on same day as diarrhea began	0.19	0.18	0.19	0.18	0.07	0.07
	(0.11 to 0.27)	(0.11 to 0.25)	(0.11 to 0.27)	(0.10 to 0.25)	(−0.01 to 0.16)	(−0.01 to 0.15)
Used ORS + zinc	0.33	0.31	0.18	0.17	0.03	0.03
	(0.22 to 0.44)	(0.21 to 0.41)	(0.09 to 0.28)	(0.08 to 0.26)	(−0.06 to 0.13)	(−0.06 to 0.12)
Used antibiotics	−0.07	−0.08	−0.05	−0.05	0.04	0.03
	(−0.14 to 0.01)	(−0.14 to −0.01)	(−0.12 to 0.03)	(−0.12 to 0.02)	(−0.03 to 0.12)	(−0.04 to 0.10)
**Ex-Post Outcome**						
Used zinc	0.29	0.27	0.15	0.15	0.02	0.02
	(0.18–0.41)	(0.17 to 0.37)	(0.06–0.24)	(0.06 to 0.23)	(−0.08 to 0.12)	(−0.07 to 0.12)

95% CIs from logistic regression in parentheses. Differences estimated using recycled predictions from logistics regressions (see S1 Appendix Section S2.1). Standard errors clustered at the village level and estimated using the Delta method [28]. Adjusted models control for prespecified control variables including branch fixed-effects, caretaker characteristics (age, education, number of children), child characteristics (age, diarrhea frequency, blood in stool, concurrent fever), household characteristics (water source, latrine type, main source of income), and baseline village characteristics (percentage of households using respective treatment, percentage of households visited by CHW in past month, percentage of households aware of free ORS in village, percentage of households with ORS stored in their home). See S1 Appendix Table A for full regression results. Secondary outcomes were prespecified, and *P* values were adjusted for multiple hypotheses (see S1 Appendix Section S6). Ex-post outcome was not prespecified and was requested ex-post by an anonymous reviewer.

**Abbreviations:** CHW, community health worker; ORS, oral rehydration salts.

ORS + zinc coverage was more than twice as large in the free and convenient group relative to the control group (63.5% versus 30.7%; adjusted difference of 31 percentage points; 95% CI 21–41; *P <* 0.001) and 40% larger in the free and convenient group relative to convenient only (63.5% versus 45.3%; adjusted difference of 17 percentage points; 95% CI 9–26; *P <* 0.001). The gap in ORS + zinc use between free and convenient and free only was small and not statistically significant (adjusted difference of 3 percentage points; 95% CI −6 to 12; *P* = 0.603).

Free and convenient distribution also reduced the time to ORS use; 19.5% of cases in the control arm and 19.6% in the convenient only arm were treated with ORS on the same day as diarrhea onset. In the free and convenient group, 38.7% of cases were treated on the same day (adjusted difference of 18 percentage points; *P <* 0.001 for both). Free and convenient distribution also increased ORS use on the same day as diarrhea onset by 7 percentage points relative to the free only arm, although results were not statistically significant (adjusting for controls, 95% CI −1 to 15). Results were similar with a Cox proportional hazard model estimating days to ORS use (truncated at 7 days, S1 Appendix Table B).

In the control group, 26.3% of cases used an antibiotic, which was modestly higher than the 19.3% usage in the free and convenient group (adjusted difference of −8 percentage points; 95% CI −14 to −1; *P* = 0.04). This is the only result we report in which including covariates makes a difference, as the raw gap is only statistically significant at the 8% level. There was no statistically significant difference in antibiotic use among the several treatment arms.

Exposure to the interventions was modest, suggesting that effects could be larger with better implementation (Table 4). Just over 60% of households in villages assigned to the free and convenient group reported a free delivery of ORS, under 20% of households in the convenient only group received an offer to sell ORS at the home, and 42% of households in the free only group received vouchers. There was also some spillover of the interventions; 9.8% and 17.6% of households in the convenient only and free only groups, respectively, received a free delivery of ORS + zinc, both statistically significant increases over the control group (*P <* 0.01 for both).

**Table 4:** Intermediate outcomes in 4 weeks following interventions (recall = last 4 weeks).

	Control	Free and Convenient	Convenient Only	Free Only
**Exposure to Interventions (bolded numbers represent the share of households that reported receiving the assigned intervention)**				
Received preemptive delivery^a^	3.1%	60.5%^***^	9.8%^***^^###^	17.6%^***^^###^
Received home sales offer^a^	8.1%	11.9%	19.4%^***^^#^	11.9%
Received voucher^a^	2.2%	4.4%	2.4%	42.4%^***^^###^
**Take-Up (in Last 4 Weeks) and Home Storage**				
Obtained any ORS^a^	24.7%	70.1%^***^	37.2%^***^^###^	54.3%^***^^###^
ORS stored when diarrhea episode began^b^	8.5%	50.3%^***^	15.7%**^###^	33.3%^***^^###^
Obtained any zinc^a^	14.7%	67.1%^***^	28.1%^***^^###^	49.7%^***^^###^
Zinc stored when diarrhea episode began^b^	6.8%	48.1%^***^	11.2%*^###^	31.0%^***^^###^
**Treatment Seeking and CHW Contact in Last 4 Weeks**				
Received home visit from CHW^b^	23.7%	61.0%^***^	35.5%**^###^	55.3%^***^
Visited CHW’s home^b^	18.7%	27.2%*	22.9%	44.1%^***^^###^
Sought treatment outside home^b^	65.8%	52.6%^***^	70.5%^###^	66.6%^###^
Sought treatment from non-CHW^b^	59.8%	35.2%^***^	54.4%^###^	39.0%^***^

^a^Includes all caretakers.

^b^Includes only caretakers that reported a diarrhea episode in last 4 weeks.

^*^P < 0.1 relative to control.

^**^P < 0.05.

^***^P < 0.01.

^#^*P <* 0.1 relative to preemptive delivery.

^##^*P <* 0.05.

^###^*P <* 0.01

**Abbreviations:** CHW, community health worker; ORS, oral rehydration salts.

Intermediate outcomes (take-up and storage of ORS) were as expected (Table 4). All treatment arms had more interaction with the CHWs and more take-up of ORS than did the controls. The free and convenient group had more ORS take-up and home storage than the free only and convenient only arms (*P <* 0.001 for all). This suggests that the interventions worked through the expected channels.

CHWs assigned to free and convenient or free only distribution made more home visits than CHWs assigned to convenient only (Table 4). Follow-up interviews with CHWs suggest that they avoided sales visits when they expected a household to decline. In contrast, delivering free ORS or vouchers was appealing to CHWs because it increased status and/or invoked altruism. These results suggest that instructions to charge for ORS reduces CHW effort (in addition to reducing household demand).

We used several strategies to validate our primary outcome of self-reported ORS use. First, we paid households in the free and convenient group a small incentive to retain the packaging for the treatment delivered by the CHWs (both used and unused). Although most households disposed of empty packets, 92% of households that reported using ORS had fewer packets to show than they reported obtaining, suggesting that the missing packets had indeed been used (S1 Appendix Section S3 and Table C). Next, we used negative controls (placebo tests) to assess the impact of the interventions on behaviors related to child health that should not be affected by the interventions: malaria treatment, bed net usage, consumption of unclean food or liquid, and hand washing. We found no statistically significant effect of any of the treatments on these placebo outcomes (S1 Appendix Table D). Finally, we used shorter recall periods to mitigate recall bias (i.e., any diarrhea in the last 7 days [instead of 4 weeks] and cases ongoing at the time of the survey). Point estimates from these analyses are similar to the results in Table 3 (S1 Appendix Table E).

We were also concerned about differential reporting of diarrhea episodes, which could compromise the comparability of the groups. To bound this potential bias, we assumed that prevalence in the free and convenient group was identical to that in the control group, adding about 71 cases to the free and convenient group. We assumed that none of these cases used ORS, which reduced ORS coverage in the free and convenient group to 69%, still a 13 percentage point increase over the control group (*P <* 0.001). Thus, potentially differential reporting of a diarrhea episode cannot fully explain the higher ORS use in the free and convenient arm.

## Discussion

Instructing CHWs to distribute ORS + zinc for free (either with free delivery ahead of illness or with a voucher) substantially increased household usage of these products relative to a sales model. Relative to the status quo, the free and convenient distribution model increased ORS coverage by 37% (21 percentage points) and more than doubled ORS + zinc coverage (33 percentage point increase). Moreover, only 60% of households actually received the free home deliveries, suggesting that usage could be improved further with better CHW adherence. These results suggest that CHW programs that sell ORS + zinc should consider switching to free distribution.

Our study is the first we are aware of to examine the causal impact of the price of ORS + zinc on utilization [18]. Households in the free and convenient arm had 19% (12 percentage points) higher ORS coverage and 40% (18 percentage points) higher ORS + zinc coverage relative to households in the convenient only arm. This suggests that price is an important barrier to ORS + zinc use and has implications beyond CHW programs. Other efforts to increase ORS + zinc coverage should focus on increasing access to free treatment (e.g., through preemptive free distribution during child immunization and health check-ups or through public-private partnerships with private health clinics).

Part of the lower usage in the convenient only arm was driven by fewer CHW visits than in the other treatment arms. CHWs in the free and convenient and free only arms were nearly twice as likely as CHWs in the convenient only arm to visit a given household. In addition to distributing either ORS or vouchers, these added visits in the free distribution arms may have increased ORS use through increased ORS education or by making ORS use more salient.

The lower share of households visited by a CHW in the convenient only arm surprised us because CHWs in this arm retained 100% of sales revenue as profits. Ex-post conversations with the CHWs in the home sales arm revealed that they found sales visits unpleasant. In contrast, CHWs liked free distribution because it increased their status in the community and may have invoked altruism. These results suggest that sales visits carried a social penalty, whereas free distribution was socially rewarding.

There is growing support for entrepreneurial CHW models [23,29]. However, our study suggests that a market-driven model can both lower ORS demand from households and reduce CHW motivation (compared to free distribution). Our findings add to the existing evidence that prosocial incentives are sometimes more effective than financial incentives at motivating health workers [30,31].

Ours is also the first study we are aware of to examine the causal impact of convenience on ORS use [18]. There was no statistically significant difference in ORS use between free and convenient distribution and free only distribution arms, suggesting that convenience was not an important barrier to use in this setting. Most caretakers were willing to endure the small hassle cost of retrieving the free ORS from the CHW’s home. Over half of caretakers lived within a 5-minute walk to the CHW’s home. Thus, it is possible that this short distance was not enough to dampen demand. This lack of detectable effects for convenience could also partly be explained by our sampling strategy, which excluded the most distant households in the CHW’s catchment area. Therefore, in the context of our study the free and convenient arm and free only arm had very similar interventions. Lack of convenience could be a stronger barrier in less densely populated areas, where retrieval costs are larger. Indeed, people that were further from the CHW’s home were less likely to use ORS in all study arms (S1 Appendix Fig C). Future research should examine how programs such as ours affect households living further from the CHW than in our sample.

In addition to increasing ORS coverage, free and convenient distribution also reduced the time between diarrhea onset and ORS initiation. Mortality from diarrhea can occur quickly, and treatment guidelines recommend immediate initiation of ORS after the first symptoms [17]. Accelerating the start of ORS could provide meaningful health benefits. It appears that the reduction in time to ORS initiation was partly driven by a convenience effect—making ORS more convenient through preemptive delivery made ORS use happened quicker than requiring retrieval from the CHW’s home—but this result was not statistically significant at conventional levels. However, many caretakers in the free only arm redeemed their vouchers prior to a child having a diarrhea episode (33% of cases had ORS stored when the episode began, relative to only 8% in control villages; Table 4). Thus, these households already had ORS conveniently available when the child came down with diarrhea. This preemptive retrieval could partly explain why we do not observe a convenience effect.

This is one of the first randomized trials studying ways to increase ORS use [18,23,32]. A recent systematic review found a shortage of rigorous studies that evaluated interventions to increase ORS use [18]. In work since this review, Bjorkman, et al. (2016) used an RCT to examine the impact of the BRAC CHW program on ORS use in Uganda (relative to no CHWs) and found that the program led to a modest increase in ORS + zinc coverage (from 33% to 38%) [23]. Our results suggest that simply switching to free CHW distribution of ORS + zinc can lead to ORS + zinc coverage levels over 60%, a substantial improvement over the status quo.

This study also contributes to our limited understanding of why ORS use remains stagnant [8,13]. Although there is a substantial amount of resources allocated towards increasing ORS use, there is little evidence on whether these interventions are targeting the right barriers. Our results suggest that resources should be allocated towards increasing access to free ORS.

Free distribution of ORS + zinc could be scaled up by BRAC without much additional effort from health workers or administrators—supply chains are already in place, BRAC already distributes ORS to CHWs monthly (at a cost), and BRAC already instructs CHWs to make household visits. Many CHWs in Uganda and elsewhere sell ORS (e.g., BRAC’s CHWs are in over 3,000 villages in Uganda and in 12 other countries). Thus, having CHWs switch to providing ORS for free is a simple intervention that could lead to large health improvements. For example, if all of BRAC’s 3,000 CHWs in Uganda switched to free distribution of ORS, this intervention would result in about 14,400 additional cases treated with ORS per month, saving about 19 lives per month (see S1 Appendix Section S4 for calculation details). If scale-up in areas where there is no existing CHW or where CHWs are regularly not stocked with ORS supply would require more up-front effort. However, the impact of free and convenient ORS distribution could be even larger in these areas because they are likely to have lower baseline ORS coverage (see Fig 3).

Although the price of ORS (US$0.15) appears to be a barrier to use for caretakers, it is cheap from an implementer perspective—it would cost less than US$10 per month to implement the free preemptive delivery program in one village. Because ORS is one of the most cost-effective child health interventions available [33], switching from charging to free distribution is extremely cost-effective. In preliminary work, we estimate that switching to free and convenient distribution would cost only US$64 per disability-adjusted life year averted [34].

Our results cannot be extrapolated to other health products. However, this work adds to a growing body of literature suggesting that free distribution of other health products substantially increases product coverage (e.g., point-of-use water treatment, bed nets, and deworming medication) [20]. This suggests that having CHWs distribute other products for free might also be the preferred strategy.

A key limitation of this study is that we only identified short-term impacts. It is not clear whether the effectiveness of free distribution will continue in the long run if scaled up. Moreover, a model of free distribution might not be financially sustainable over time. However, governments throughout the world have been distributing ORS for free for several decades. In Uganda, 88% of public facilities have ORS stocked, suggesting that, in the public sector experience, free distribution is financially sustainability [35]. Moreover, children treated for diarrhea in the public sector are much more likely to be treated with ORS than those treated in the private sector [12]. Free distribution of other health products—e.g., deworming pills, chlorine for drinking water, and bed nets—have been scaled up with great success by private organizations in a sustainable way [36–39]. Although private nongovernmental organizations, such as Population Services International (PSI), initially “socially marketed” these products, evidence on the benefits of free distribution [21] changed practice, and now PSI distributes many products for free [36].

Another potential concern not captured in our short follow-up period is that free distribution could reduce the number of private providers who stock ORS. This could dilute the effectiveness of our intervention. Free distribution of other health products has not been shown to effect local markets [40], but more evidence is needed to understand the private sector response to an increase in free ORS.

This study has several other limitations. First, our measure of ORS use relies on caretaker reports. Although caretaker reports are used to monitor ORS use globally, self-reported data rely on accurate memory and could be subject to social desirability bias. We demonstrated that comparing observed packets to total packets obtained produced similar ORS coverage estimates as caretaker reports (S1 Appendix Section S4). However, we were only able to count packets in the free and convenient group and therefore were not able to use this measure as an outcome. Future studies should identify a more robust way of measuring ORS use. Second, our sample is not representative of the rest of Uganda. All villages had a CHW present in the village at baseline, which could explain above average baseline ORS use (60% compared to a 46% country average). Moreover, most villages were peri-urban, whereas much of Uganda is rural. Therefore, it is unclear what these effects would look like if scaled up nationwide. However, we find that the effect of free and convenient distribution is larger for households in villages with lower baseline ORS use, which are more representative of the rest of Uganda (Fig 3). Therefore, it seems plausible to expect that the effect of free and convenient distribution would be at least as large in the short term if scaled up to other Ugandan villages. Finally, 9.8% and 17.6% of CHWs in the convenient only and free only arms delivered treatment for free, which attenuates our estimates of the role price and the role of convenience. Informal conversations with CHWs and the study team revealed that they were socially rewarded for giving treatment away for free, which could explain this deviation from the study protocol. However, CHW deviation from protocol is likely to play out upon scale-up as well. Thus, the extra free delivery in these groups could be an accurate portrayal of the true effect of each intervention.

ORS + zinc is extremely effective at preventing mortality from diarrhea, yet it remains largely underused. As a result, hundreds of thousands of children die each year. This study suggests that having CHWs provide these products for free rather than charging has the potential to save many of these lives. Implementers of CHW programs should consider free distribution of ORS + zinc.

## Supporting information

S1 CONSORT Checklist Completed CONSORT checklist for cluster randomized trials. CONSORT, Consolidated Standards of Reporting Trials.
(DOCX)

S1 Appendix Supplementary appendix. (PDF)

S1 Protocol Study protocol and pre-analysis plan registered at AEARCTR-0001288. (PDF)

S1 Data Study data in csv format. (CSV)

S1 Replication Code Stata replication code to create figures from main text. (TXT)

S2 Replication Code Stata replication code to create tables from main text. (TXT)

## Abbreviations

CHW: community health worker
ITT: intention to treat
ORS: oral rehydration salts
PSI: Population Services International
RCT: randomized controlled trial

## References

[1] Liu L, Oza S, Hogan D, Perin J, Rudan I, Lawn JE, et al Global, regional, and national causes of child mortality in 2000–13, with projections to inform post-2015 priorities: an updated systematic analysis. The Lancet. 2015;385(9966):430–40. 10.1016/S0140-6736(14)61698-6 25280870

[2] Cash RA, Nalin DR, Rochat R, Reller LB, Haque ZA, Mizanur Rahman ASM. A Clinical Trial of Oral Therapy in a Rural Cholera-Treatment Center. Am J Trop Med Hyg. 1970;19(4):653–6. 5425504 10.4269/ajtmh.1970.19.653

[3] Pierce NF, Sack RB, Mitra RC, Banwell JG, Brigham KL, Fedson DS, et al Replacement of water and electrolyte losses in cholera by an oral glucose-electrolyte solution. Ann Intern Med. 1969;70(6):1173–81. 5789507 10.7326/0003-4819-70-6-1173

[4] Santosham M. Oral Rehydration Therapy of Infantile Diarrhea A Controlled Study of Well-Nourished Children Hospitalized in the United States and Panama. N Engl J Med. 1982;306(18):1070–6. 10.1056/NEJM198205063061802 7040950

[5] Spandorfer PR, Alessandrini EA, Joffe MD, Localio R, Shaw KN. Oral Versus Intravenous Rehydration of Moderately Dehydrated Children: A Randomized, Controlled Trial. Pediatrics. 2005;115(2):295–301. 10.1542/peds.2004-0245 15687435

[6] Munos MK, Walker CLF, Black RE. The effect of oral rehydration solution and recommended home fluids on diarrhoea mortality. Int J Epidemiol. 2010;39(suppl 1):i75–i87. 20348131 10.1093/ije/dyq025 PMC2845864

[7] Lancet. Water With Sugar And Salt. Lancet. 1978;2(8084):300–1. 79090

[8] Forsberg BC, Petzold MG, Tomson G, Allebeck P. Diarrhoea case management in low- and middle-income countries: an unfinished agenda. Bull World Health Organ. 2007;85:42–8. 10.2471/BLT.06.030866 17242757 PMC2636206

[9] Pantenburg B, Ochoa TJ, Ecker L, Ruiz J. Use of Commercially Available Oral Rehydration Solutions in Lima, Peru. Am J Trop Med Hyg. 2012;86(6):922–4. 10.4269/ajtmh.2012.11-0581 22665594 PMC3366533

[10] Ram PK, Choi M, Blum LS, Wamae AW, Mintz ED, Bartlett AV. Declines in case management of diarrhoea among children less than five years old. Bull World Health Organ. 2008;86:E-F. 10.2471/BLT.07.041384 PMC2647400 18368194

[11] Santosham M, Chandran A, Fitzwater S, Fischer-Walker C, Baqui AH, Black R. Progress and barriers for the control of diarrhoeal disease. The Lancet. 2010;376(9734):63–7. 10.1016/S0140-6736(10)60356-X 20609988

[12] Sood N, Wagner Z. Private Sector Provision of Oral Rehydration Therapy for Child Diarrhea in Sub-Saharan Africa. Am J Trop Med Hyg. 2014;90(5):939–44. 10.4269/ajtmh.13-0279 24732456 PMC4015590

[13] Sreeramareddy CT, Low Y-P, Forsberg BC. Slow progress in diarrhea case management in low and middle income countries: evidence from cross-sectional national surveys, 1985–2012. BMC Pediatr. 2017;17(1):83 10.1186/s12887-017-0836-6 28320354 PMC5360044

[14] DHS. Uganda Demographic and Health Survey. 2016.

[15] Aggarwal R, Sentz J, Miller MA. Role of zinc administration in prevention of childhood diarrhea and respiratory illnesses: a meta-analysis. Pediatrics. 2007;119(6):1120–30. 10.1542/peds.2006-3481 17545379

[16] Bhutta Z, Black RE, Brown K, Gardner JM, Gore S, Hidayat A, et al Prevention of diarrhea and pneumonia by zinc supplementation in children in developing countries: pooled analysis of randomized controlled trials. J Pediatr. 1999;135(6):689–97. 10586170 10.1016/s0022-3476(99)70086-7

[17] USAID/UNICEF/WHO. Diarrhoea Treatment Guidelines: Including new recommendations for the use of ORS and zinc supplementation for clinic-based healthcare workers Arlington, VA: USAID Micronutrient Program; 2005.

[18] Lenters LM, Das JK, Bhutta ZA. Systematic review of strategies to increase use of oral rehydration solution at the household level. BMC Public Health. 2013;13(3):S28. 24564428 10.1186/1471-2458-13-S3-S28 PMC3847633

[19] Dupas P, Miguel E. Impacts and determinants of health levels in low-income countries Handbook of economic field experiments. 2: Elsevier; 2017 p. 3–93.

[20] Kremer M, Glennerster R. Improving Health in Developing Countries. Handbook of Health Economics. 2011;2:201–315.

[21] Cohen J, Dupas P. Free distribution or cost-sharing? Evidence from a randomized malaria prevention experiment. The Quarterly Journal of Economics. 2010:1–45.

[22] Dupas P, Hoffmann V, Kremer M, Zwane AP. Targeting health subsidies through a nonprice mechanism: A randomized controlled trial in Kenya. Science. 2016;353(6302):889–95. 10.1126/science.aaf6288 27563091 PMC5003414

[23] Bjorkman Nyqvist M, Guariso A, Svensson J, Yanagizawa-Drott D. Effect of a micro entrepreneur-based community health delivery program on under-five mortality in Uganda: a cluster-randomized controlled trial. 2016.

[24] Uganda Bureau of Statistics (UBOS). Uganda National Household Survey 2012/2013. Kampala, Uganda; 2014.

[25] Rogers W. Regression standard errors in clustered samples. Stata Technical Bulletin. 1994;3(13).

[26] Abadie A, Athey S, Imbens GW, Wooldridge J. When Should You Adjust Standard Errors for Clustering?: National Bureau of Economic Research; 2017.

[27] Anderson ML. Multiple inference and gender differences in the effects of early intervention: A reevaluation of the Abecedarian, Perry Preschool, and Early Training Projects. Journal of the American Statistical Association. 2008;103(484):1481–95.

[28] Efron B, Tibshirani R. Bootstrap methods for standard errors, confidence intervals, and other measures of statistical accuracy. Statistical Science. 1986:54–75.

[29] Kopf D. Health workers can be wildly successful at saving lives when they have a profit motive. Quartz. 2016.

[30] Ashraf N, Bandiera O, Jack BK. No margin, no mission? A field experiment on incentives for public service delivery. Journal of Public Economics. 2014;120:1–17.

[31] Deserranno E. Financial Incentives as Signals: Experimental Evidence from the Recruitment of Village Promoters in Uganda mimeo Northwestern University; 2016.

[32] Aung T, McFarland W, Khin HSS, Montagu D. Incidence of pediatric diarrhea and public–private preferences for treatment in rural Myanmar: a randomized cluster survey. J Trop Pediatr. 2012;59(1):10–6. 10.1093/tropej/fms033 22874876

[33] Black RE, Levin C, Walker N, Chou D, Liu L, Temmerman M, et al Reproductive, maternal, newborn, and child health: key messages from Disease Control Priorities 3rd Edition. The Lancet. 2016;388(10061):2811–24. 10.1016/S0140-6736(16)00738-8 27072119

[34] Wagner Z. Working With Community Health Workers to Increase Use of ORS and Zinc to Treat Child Diarrhea In Uganda: A Cluster Randomized Controlled Trial [Dissertation]: UC Berkeley; 2017.

[35] Ministry of Health (MOH) [Uganda] and Macro International Inc. Uganda Service Provision Assessment Survey 2007 Kampala, Uganda: Ministry of Health and Macro International Inc; 2008.

[36] Population Services International. Long-Lasting Insecticide-Treated Nets 2018. Available from: https://www.psi.org/program/long-lasting-insecticide-treats-nets/. [cited 2018 Oct 16].

[37] Evidence Action. Deworm The World 2018. Available from: https://www.evidenceaction.org/dewormtheworld/. [cited 2018 Oct 16].

[38] Evidence Action. Safe Water Dispensers 2018. Available from: https://www.evidenceaction.org/dispensersforsafewater/#track-record. [cited 2018 Oct 16].

[39] Eze IC, Kramer K, Msengwa A, Mandike R, Lengeler C. Mass distribution of free insecticide-treated nets do not interfere with continuous net distribution in Tanzania. Malar J. 2014;13(1):196. 24884786 10.1186/1475-2875-13-196 PMC4046070

[40] Wydick B, Katz E, Janet B. Do in-kind transfers damage local markets? The case of TOMS shoe donations in El Salvador. Journal of Development Effectiveness. 2014;6(3):249–67.

