# Supplementary material for: The role of price and convenience in use of oral rehydration salts to treat child diarrhea: A cluster randomized trial in Uganda
Source: PLoS Med. 2019 Jan 24;16(1):e1002734. doi: 10.1371/journal.pmed.1002734 (PMC6345441; doi:10.1371/journal.pmed.1002734)
Supplement: S1 Appendix — (PDF) [file pmed.1002734.s002.pdf]

The role of price and convenience in underuse of oral rehydration salts: a cluster randomized trial in Uganda

Zachary Wagner[1*], John Bosco Asiimwe[2], William H. Dow[3], David I. Levine[4]

December 14, 2018

Technical Appendix

[1]RAND Corporation, Santa Monica, CA, USA
[2]School of Planning and Statistics, Makerere University, Kampala, Uganda
[3]School of Public Health, University of California at Berkeley, Berkeley, CA, USA
[4]Haas School of Business, University of California at Berkeley, Berkeley, CA, USA
[*]Corresponding Author: Please send inquiries to zwagner@berkeley.edu

Contents

1 Training of Community Health Workers **3**

2 Regression Analysis Details **6**

2.1 Main regression specification . . . 6

2.2 Multinomial logit model to test for balance between groups . . . 7

3 Packet Counting **7**

4 Estimating Deaths Averted Per Month **8**

5 Efforts to avoid contamination **9**

6 Description of adjustment for multiple inference based on Anderson (2008) **9**

List of Figures

A Map of Study Villages . . . 11

B Flier used to provide ORS and zinc knowledge to caretakers . . . 12

C ORS coverage by proximity to CHW's home . . . 13

D Missing ORS from CHW inventory vs. number of packets reported by households 14

List of Tables

A Full Regression Result from Primary Analysis in Table 3 . . . 15

B Time to ORS Use After Diarrhea Onset: Cox Proportional Hazard Model . . . 17

C Validating Self Report Using Packet Counting . . . 18

D Placebo Tests (Health Behaviors That Should Not Be Affected) . . . 19

E Impact On ORS Use (Shorter Recall Periods) . . . 20

1 Training of Community Health Workers

Community health workers assigned to one of the intervention arms were invited to local BRAC offices (6 branches in total) for a 1 hour training session on how to carry out the intervention. The study team called each the CHWs ahead of time to ensure that every CHW showed up. Training was conducted in Lugand (the local language) and adhered to a strict protcol. The trainings for the differnt treatment arms were conducted seperately and were scheduled so that there would not be any overlap. CHWs were also asked not to discuss the training with any of the other CHWs. Trainings were structured to be identical across treatment groups aside from instructions on ORS and zinc distribution. The trainings started after Below is a summary of the instructions provided to CHWs during the trainings (same order for each intervention):

1. Visit all households with child under-5
2. Ask for main caretaker
3. Intervention Specific [provide free ORS+zinc/offer to sell ORS+zinc/provide voucher]
4. Provide standard information on using ORS and zinc (show caretaker flier, Appendix Figure 1)
5. Re-visit a household if primary caretaker is not home
6. Visit closest households first
7. We will check to make sure these tasks are followed appropriately
8. We will pay you 12 USD to make these household visits (half now, half in one month after verification of intervention)
9. Start immediately
10. Should take about 3 days
11. Don't discuss this with other CHWs
12. Please keep any remaining ORS and zinc for our records

The order of the instructions was kept the same across all interventions and item (3) was the only area where the instructions differed. After the trainings, we allowed CHWs to ask any questions they might have about the interventions and distributed the first half of their payment ($6) plus payment for transportation. Below is the script for each of the different treatment arms

Script for free+convenient arm:
In the next few days, please visit all of the households in your village that have a child under 5-years-old. When you visit the households, we would like you to provide the primary caretaker with two packets of ORS and 1 strip of zinc for each child under 5-years olds in home. If there are 2 children under-5 then please provide 4 packets of ORS and 2 strips of zinc. We will provide you with compensation for making these deliveries. Please inform the caretaker that this is a 1-time gift, and that you would like the caretaker to keep these products stored in the home for future use. There will not be any more free ORS given away. Inform the caretaker that the products should be stored in a safe and dry place. In addition, please give the caretaker the standard information on ORS and zinc (Show flier). If the primary caretaker is not home, please revisit the household at a later time to ensure that all households receive their free ORS

and zinc. Please make these deliveries to the closest households to your home first, in case you run out of ORS and zinc.

It is very important that you leave the ORS and zinc at the home with caretakers. Please do not store the ORS and zinc yourself for the caretaker. The only reason for you to not leave the ORS and zinc with the caretaker is if the caretaker expresses that they do not want the ORS and zinc.

In addition, please ask the caretakers to retain the ORS and zinc packaging after they use it. There will be a research team that comes to some the households in your village in about 1 month, and caretakers will be provided with a small compensation if they still have the ORS packaging, whether it is used or unused. We just want to see the used or unused packaging for our records. For example, if a caretaker uses ORS to treat her child's diarrhea and if they retain the packaging, they will be paid for it. Similarly, if a caretaker has unused ORS packages to in the home to show, they will also be paid. However, if they do not have the packaging (whether used or unused) they will not be paid. If 5 weeks pass and no one has come to a caretaker's home to distribute compensation, the household is free to discard the packaging as all compensation will have already been given out.

We expect that it will take you up to 3 days to make all of these deliveries. Please start making these deliveries as soon as possible, ideally starting tomorrow. Once you are finish with the deliveries, please go back to carrying out your duties as you normally would. You are free to keep any remaining ORS and zinc if you have extra. Please keep any remaining ORS stored in your home, as we would like to take inventory at a later date.

Please do not discuss this with any of the other CHPs at this branch that are not here. We only have enough resources to do this with a few of you, so not all CHPs get the free ORS and zinc and the payment. Also, please do not give any of the ORS or zinc to any of the other CHPs as this will compromise our study. It is important that you use all of these products for your community.

Script for convenient only arm:
We are going to give all of you ORS and zinc for free. In the next few days, we would like to visit all of the households in your village that have a child under 5-years-old. We will provide you with compensation for making these visits. When you visit the households, please bring some of this ORS and zinc with you to sell. We would like you to ask for the primary caretaker and give the caretaker the standard information on ORS and zinc (Show flier). Once you provide this information, please offer to sell the primary caretaker two of these ORS packets and one of these strips of zinc at 500 UGX for1 ORS packet and 1000 UGX for 10 tabs of zinc. You can use the money from any sales that you make. It is yours! It is very important that you do not give away this ORS for free at any point. It is okay for caretakers to pay with credit if you believe that they will they will get paid back. Inform the caretaker that this is the standard course of treatment for child diarrhea. Although we would like you to offer two packets of ORS and 1 strip of zinc, the caretaker is free to purchase as much or as little ORS as they would like. Tell the caretaker that a child will surely come down with diarrhea in the future, and purchase now will ensure that the ORS and zinc are readily available when this happens. It is important to start treatment right away when the child comes down with diarrhea and this will allow them to start treatment sooner.

If purchased, inform the caretaker that the products should be stored in a safe and dry place. If the primary caretaker is not home, please revisit the household at a later time until you have offered to sell ORS and zinc to all caretakers of children under-5 in your village.

We expect that it will take you up to 3 days to make all of these visits. Please start making these home visits as soon as possible, ideally starting tomorrow. Once you finish the visits, please go back to carrying out your duties as you normally would. You are free to keep any remaining ORS and zinc that you do not sell during these visits for future sale, but we ask that you do not give any away for free. Please keep any ORS and zinc that you don't sell stored in your home and don't mix it with other ORS and zinc that you might have stored already, as we would like to take an inventory at a later date (in about a month).

Please do not discuss this any of the other CHPs at this branch that are not here. We only have enough resources to do this with a few of you, so not all CHPs get the free ORS and zinc and the payment. Also, please do not give any of the ORS or zinc to any of the other CHPs as this will compromise our study. It is important that you use all of these products for your community.

Script for free only arm:
We are going to give all of you ORS and zinc for free and we would like you to keep this ORS and zinc stored in your home for free distribution to your community. This is one-time free distribution and all children under-5 will be eligible for 2 packets of ORS and 1 strip of zinc. In the next few days, we would like you to visit all of the households in your village that have a child under 5-years-old. When you visit the households, we would like you to ask for the primary caretaker and give the caretaker the standard information on ORS and zinc (Show flier). Once you provide this information, please give the caretaker one voucher per-child under-5, which can be redeemed for 2 sachets of ORS and 10 tablets of zinc [Show Voucher]. Please provide 2 sachets of ORS and 10 tablets of zinc to any caretaker that presents you with one of these vouchers. Only provide 2 packets of ORS and 1 strip of zinc per child. It is very important that you do not give ORS and zinc away during these initial household visits, but instead require that the caretakers retrieve the ORS from your home using the voucher. Tell the caretaker that she can use the voucher whenever she pleases and that the supply will not run out, so there is no rush. Please keep any vouchers that are used for our records. It is also very important that you do not sell any of this ORS and zinc and that it is retained to be given away for free in exchange for the voucher. Once you finish off the supply of ORS and zinc that we provide you, you can go back to selling these products as you normally would. Please make these voucher deliveries to the closest households to your home first, in case you run out of vouchers.

We expect that it will take you up to 3 days to make all of these household visits and voucher deliveries and we will provide you with compensation for doing this. This compensation will also make up for any lost sales you might encounter. Please start making these visits as soon as possible, ideally starting tomorrow. Please keep this ORS and zinc stored separately from any other ORS and zinc that you might have, as we would like to take an inventory at a later date (in about a month).

Please do not discuss this any of the other CHPs at this branch that are not here. We only have enough resources to do this with a few of you, so not all CHPs get the free ORS and zinc and the payment. Also, please do not give any of the ORS or zinc to any of the other CHPs as this will compromise our study. It is important that you use all of these products for your community.

2 Regression Analysis Details

2.1 Main regression specification

We conducted two logistic regressions for each treatment outcome; one unadjusted and one adjusted. Adjusted models included pre-specified control variables including branch fixed-effects, caretaker characteristics (age, education, number of children), child characteristics (age, diarrhea frequency, blood in stool, concurrent fever), household characteristics (water source, latrine type, main source of income), and baseline village characteristics (% of households using respective treatment, % of households visited by CHW in past month, % of households aware of free ORS in village, % of households with ORS stored in their home). For secondary treatment outcomes, we adjusted p-values for multiple hypothesis testing using the free step-down re-sampling method to control the False Discovery Rate (FDR) (Anderson, 2008). We clustered standard errors at the village level. Unadjusted regressions took the following form.

$$\Pr(Y_{iv}) = \text{expit}(\beta_0 + \beta_1 FreeConvenient_{iv} + \beta_2 ConvenientOnly_{iv} + \beta_3 FreeOnly_{iv} + \epsilon_{iv}) \quad (1)$$

Where Y_{iv} is the respective treatment outcome for child i in village v; $FreeConvenient$, $ConvenientOnly$, and $FreeOnly$ are group assignment indicators, with the control group as the reference category. The β's represent the log odds of the treatment effect of each intervention relative to the control group.

Adjusted regressions took the following form:

$$\begin{aligned}\Pr(Y_{ivbt}) = \text{expit}(&\beta_0 + \beta_1 FreeConvenient_{ivb} + \beta_2 ConvenientOnly_{ivb} + \beta_3 FreeOnly_{ivb} \\ &+ \beta_4 Y_{v(t-1)b} + \boldsymbol{X_{ivb}\beta_5} + \lambda_b + \epsilon_{ivbt})\end{aligned} \quad (2)$$

Here, $Y_{v(t-1)b}$ represents share of cases treated with treatment Y in the child's village at baseline, X_{ivb} is a vector of caretaker, child, and village characteristics, and λ_b is a set of branch fixed effects.

We used these equations to estimate the following average marginal effects, which we refer to as effect sizes in the main text and in Table 3:[1]

$\mathbb{E}[Y|FreeConvenient = 1] - \mathbb{E}[Y|Control = 1]$: Effect of free and convenient distribution relative to the control group (Columns 1 and 2 of Table 3)

$\mathbb{E}[Y|FreeConvenient = 1] - \mathbb{E}[Y|ConvenientOnly = 1]$: The effect of free and convenient distribution relative to convenient only—the price-effect (Columns 3 and 4 of Table 3).

$\mathbb{E}[Y|FreeConvenient = 1] - \mathbb{E}[Y|FreeOnly = 1]$: The effect of free and convenient distribution relative to free only—the convenience effect (Columns 5 and 6 of Table 3).

Table A present full regression results for the primary outcome.

[1] In practice, we used Stata's margins command which uses the same process described here for effect sizes but also estimates standard errors using the delta method. See https://www.stata.com/support/faqs/statistics/compute-standard-errors-with-margins/ for an explanation of how standard errors are estimated.

2.2 Multinomial logit model to test for balance between groups

We used a multinomial logit regression model to test for balance between groups. The multinomial regression tests for how well a broad set of covariates jointly predict assignment to the different treatment arms. This is superior to comparing each characteristics separately, because separate comparison ignores the potential for joint significance of multiple characteristics. This model takes the following form.

$$\Pr(T_{iv}) = \text{expit}(\boldsymbol{X_{iv}}\boldsymbol{\beta} + \epsilon_{ivb}) \tag{3}$$

Where T_{ivb} is the treatment assignment (control, free and convenient, convenient only, or free only) of individual i in village v, and $\boldsymbol{X}$ is set of all covariates listed in Table 1 of the main paper. We estimate $\boldsymbol{\beta}$, a vector of coefficients that indicate the association with treatment assignment of each of the X's. The χ^2 test statistic associated with this model tests the null hypothesis that the sum of all the model coefficients is equal to zero ($\sum \boldsymbol{\beta} = 0$). If the χ^2 test statistic rejects the null hypothesis (i.e., $p < 0.05$), this is indicative of imbalance. As we report in the main text, we reject the null hypothesis from this model, which is implies imbalance, and motivates the importance of controlling for the variables in Table 3..

3 Packet Counting

In the free and convenient group, CHWs instructed caretakers to keep the ORS and zinc packets that were delivered (used and/or unused), and that if packets were available for our survey team to observe, they would be provided a small incentive (about $0.30 (USD)). It was not feasible to incentivize packet retention in the other three groups, as that would have incentivized acquisition of ORS. The incentive could bias our self-reported estimates if the incentive for packet retention impacted self-reported ORS use. However, we found no difference in ORS use between the free and convenient arm (where the incentive was provided) and the convenient only arm (where no incentive was provided). Therefore, it appears the incentive had no impact on self-reported outcomes.

During the endline survey, enumerators recorded 1) if any packets were observed, 2) the number of used packets, and 3) the number of unused packets. Of the 518 diarrhea cases from households visited in the free and convenient villages[2], 59% retained at least some of the ORS/zinc packaging. However, not all households received a free delivery. Of those that reported receiving a free delivery, 80% had some the packaging left. We used the results from counting these ORS packets to create several alternative measures of ORS use that are less reliant on self-report and can be used to validate self-report measures. We used two different metrics based on observed packets to identify ORS use: 1) at least 1 empty packet observed (implying that the contents of the packet was used), and 2) fewer packets observed than obtained in the last 4 weeks.[3] We also restricted the sample in two ways to help refine the measure. First, we included only cases that received a delivery, since only households the received a delivery would be expected to have any packets to observe. Second, we included only cases where caretakers had at least 1 packet to show the enumerator. We also restricted to caretakers that had both of these criteria satisfied.

[2]Number excludes villages where no CHW carried out the intervention (40 cases)

[3]number of packets obtained was recored in an earlier survey question unrelated to counting observed packets

Results for 8 different alternative measures are presented in Table C. We present the average estimate for each sample using both the counting measures (empty packets or observed<obtained) and self-report measure. We also present estimates stratified by self-reported ORS use, which provides insight into how frequently the counting and self-reported measures are in agreement. The first thing to note is that very few caretakers had empty packets (top panel). Row 1 shows that only 34% of cases were in households that had at least one empty packet (42% of self-reported ORS users), whereas 77% of these caretakers reported using ORS. This either means that many caretakers used ORS and did not save the empty packets or they were over-reporting ORS use. Restricting the sample to those that received a delivery (row 2) improves consistency with self report, however still only 47% saved at least one empty packet (51% of self-reported ORS users). Restricting the sample further increases the likelihood of an empty packet, however agreement with the self-reported ORS users never exceeds 65% and the average measure never exceeds 58% of cases treated, substantially lower than the self-reported measure.

Many caretakers reported disposing of empty packaging, which could explain the discrepancy between observed empty packets and reported use. To account for this, we created a more flexible measure, where a case is coded as treated with ORS if the household had fewer full ORS packets than they reported acquiring in the last 4 weeks. Row 1 of panel 2 presents this measure for the full sample. Overall, we found that 75% of caretakers had fewer full ORS packets than they reported obtaining (panel 2, row 1). Moreover, among those that reported using ORS, 92% had fewer packets to show than they reported obtaining, suggesting strong agreement with the self-reported measure. Further restricting the sample improves agreement with the self-reported measure and increases the estimated share of cases treated using the counting measure. The final row of (Table C) recodes the households that reported no ORS to zero (i.e. assumes that no one under-reports ORS use), which lowers the estimated share of cases treated to 83% (column 3).

These results suggest that there was likely only a small degree of over reporting, however most households that reported ORS use had fewer packets to show than they reported obtaining. Caretakers would have had to plan out their miss-reporting in a sophisticated way to report obtaining more packets than they had available to show. Since households were paid to show their packets, it is unlikely that they would withhold packets and forgo the incentive.

4 Estimating Deaths Averted Per Month

If all of BRAC's 3,000 CHWs in Uganda that require purchase of ORS and zinc switched to free distribution, this would result in about 14,400 additional cases treated with ORS and 19 lives saved per month. Assuming that the 3,000 villages each have 24 cases per month (the average in our sample), this would be 72,000 cases per month. Under the status quo, 56% of cases get treated with ORS and under free and convenient 76% of cases get treated. This give additional cases treated as 72,000*.76-72,000*.56=14,400. Using the case specific death rate of .0014 Liu et al. (2015), this means 20.16 of these 14,400 children would die under the status quo (14,400*.0014=20.16). However, applying the effectiveness of ORS, 93% reduction in mortality rate (Munos et al., 2010), only 1.4 of these 14,400 children would die under free and convenient (14,400*.0014*.07=1.4). This gives 18.76 lives saves (20.16-1.4=18.76).

5 Efforts to avoid contamination

While the reason for a cluster-randomize controlled trial is to avoid contamination of the intervention accross individuals within a cluster, there is still the potential for contamination across clusters. Although CHW catchment areas never overlap, some catchment areas could be border eachother. As a results, we took several additional measures to avoid contamination. First, we organized the trainings for the different treatment arms on different days so that CHWs in different arms would not cross paths. Second, we set the date of the trainings to be just after the pre-scheduled monthly CHW trainings conduct by BRAC to avoid intermingling between CHWs in different arms. This means that there was no reason why CHWs in different treatment arms would cross paths during this month long intervention as a result of their profession obligations. Third, during the trainings we told all CHWs not to discuss the trainings or the intervention with other CHWs.

6 Description of adjustment for multiple inference based on Anderson (2008)

We adjusted each p-value for secondary outcomes in Table 2 of the main text according to the following steps. The following is taken from Anderson (2008).

1. Sort outcomes $y_1, ..., y_M$ in order of decreasing significance (increasing p-value), that is, such that $p_1 < p_2 < ... < p_M$. 2. Simulate the data set under the null hypothesis of no treatment effect using the resampling procedure described below.

a Draw binary treatment assignments z_{i*} from the empirical distribution of the original treatment assignments without replacement.

b Calculate the t statistic for the difference in means between treated and untreated groups.

c Repeat the procedure 100,000 times and compute the frequency with which the simulated t statistics——which have expectation zero by design——exceed the observed t statistic.

3. Calculate a set of simulated p-values, $p_1^*, ..., p_M^*$, for outcomes $y_1, ..., y_M$ using the simulated treatment status variable. Note that they will not display the same montonicity as $p_1, ..., p_M$.
4. Enforce the original monotonicity: Compute $p_r^{**} = min p_r^*, p_{r+1}^* ..., p_M^*$, where r denotes the original significance rank of the outcome, with $r = 1$ being the most significant and $r = M$ the least significant.
5. Perform $L \geq 100,000$ replications of steps 2–4.Foreach outcome y, tabulate S, the number of times that $p_r^{**} < p_r$.
6. Compute $p^{fwer*_r} = S_r/L$.
7. Enforce monotonicity a final time: $p_r^{fwer} = min p_r^{fwer*}, p_{r+1}^{fwer*} ..., p_M^{fwer*}$. (This final monotonicity enforcement ensures that larger unadjusted p-values always correspond to larger adjusted p-values.)

References

Michael L Anderson. Multiple inference and gender differences in the effects of early intervention: A reevaluation of the abecedarian, perry preschool, and early training projects. *Journal of the American Statistical Association*, 103(484):1481–1495, 2008.

Li Liu, Shefali Oza, Daniel Hogan, Jamie Perin, Igor Rudan, Joy E Lawn, Simon Cousens, Colin Mathers, and Robert E Black. Global, regional, and national causes of child mortality in 2000–13, with projections to inform post-2015 priorities: an updated systematic analysis. *The Lancet*, 385(9966):430–440, 2015.

Melinda K. Munos, Christa L Fischer Walker, and Robert E Black. The effect of oral rehydration solution and recommended home fluids on diarrhoea mortality. *International Journal of Epidemiology*, 39(suppl 1):i75–i87, 2010.

Figure A: Map of Study Villages

Enumerators recorded that 70% of villages were rural and 30% were urban. However, "rural" households in our sample lived in villages where households were relatively close together and these areas were less rural than other areas of Uganda (some villages categorized as rural could be considered peri-urban)

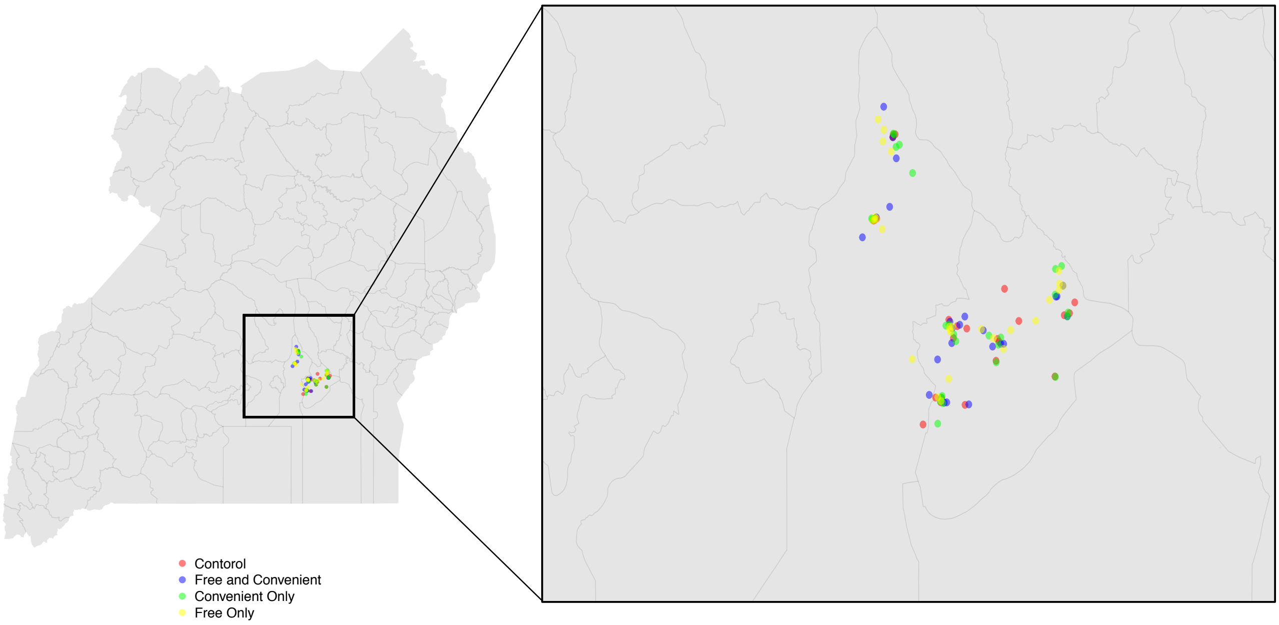

Figure B: Flier used to provide ORS and zinc knowledge to caretakers

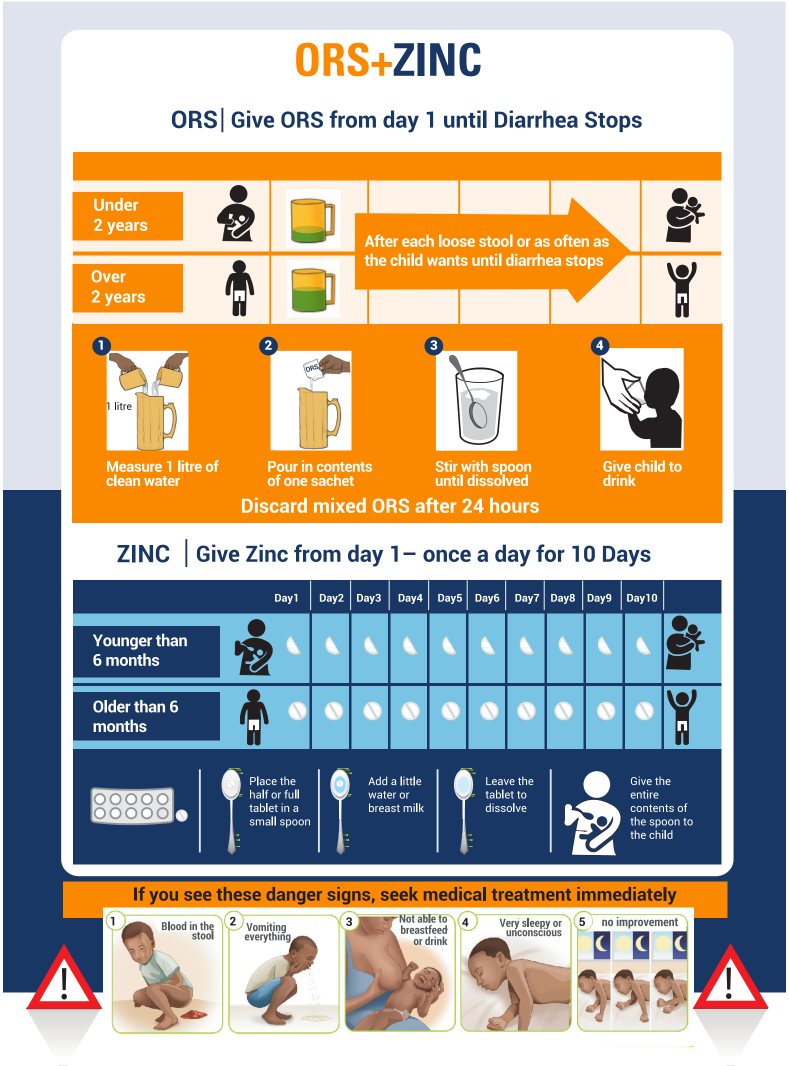

Figure C: ORS coverage by proximity to CHW's home

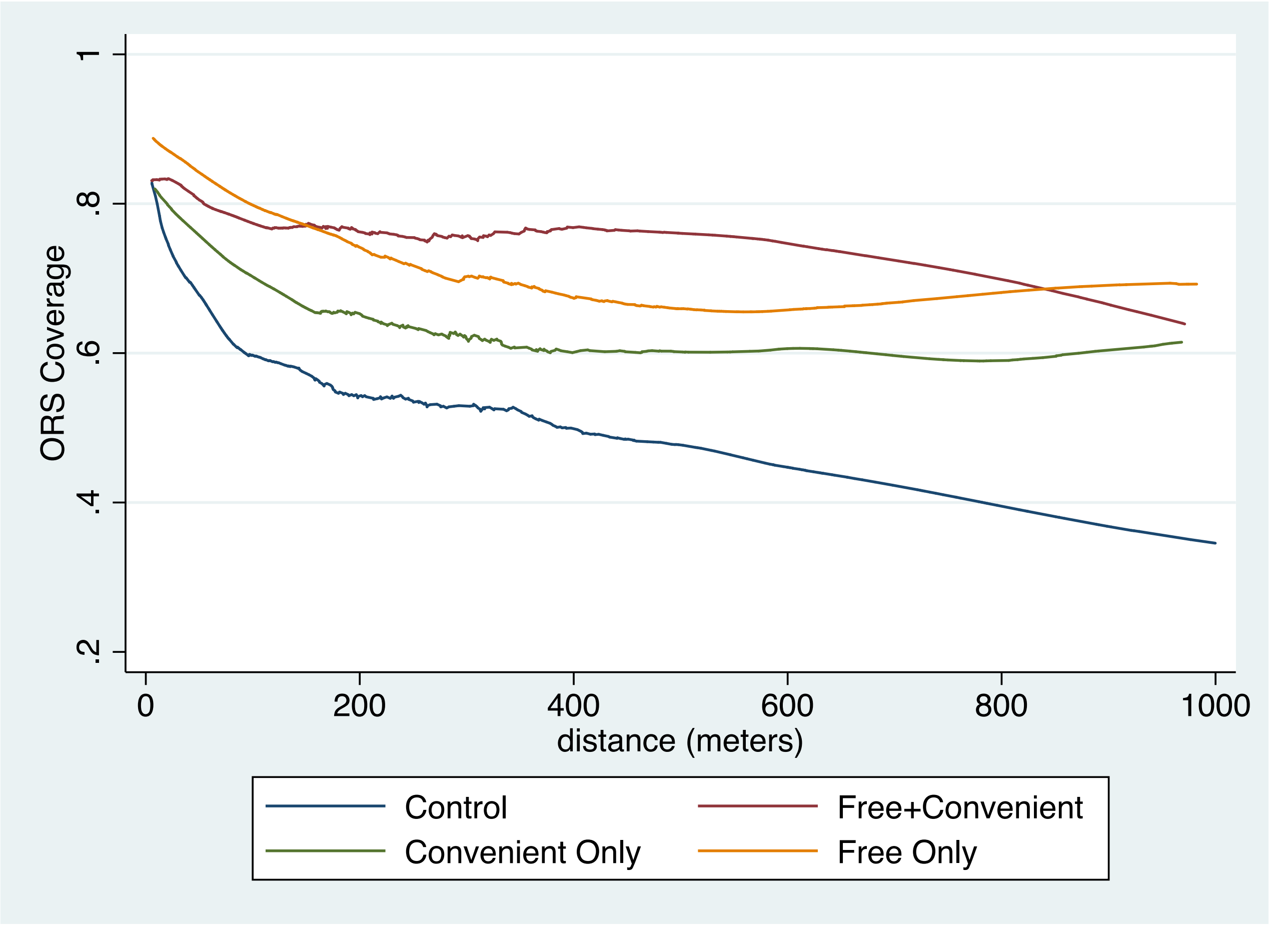

Figure D: Missing ORS from CHW inventory vs. number of packets reported by households

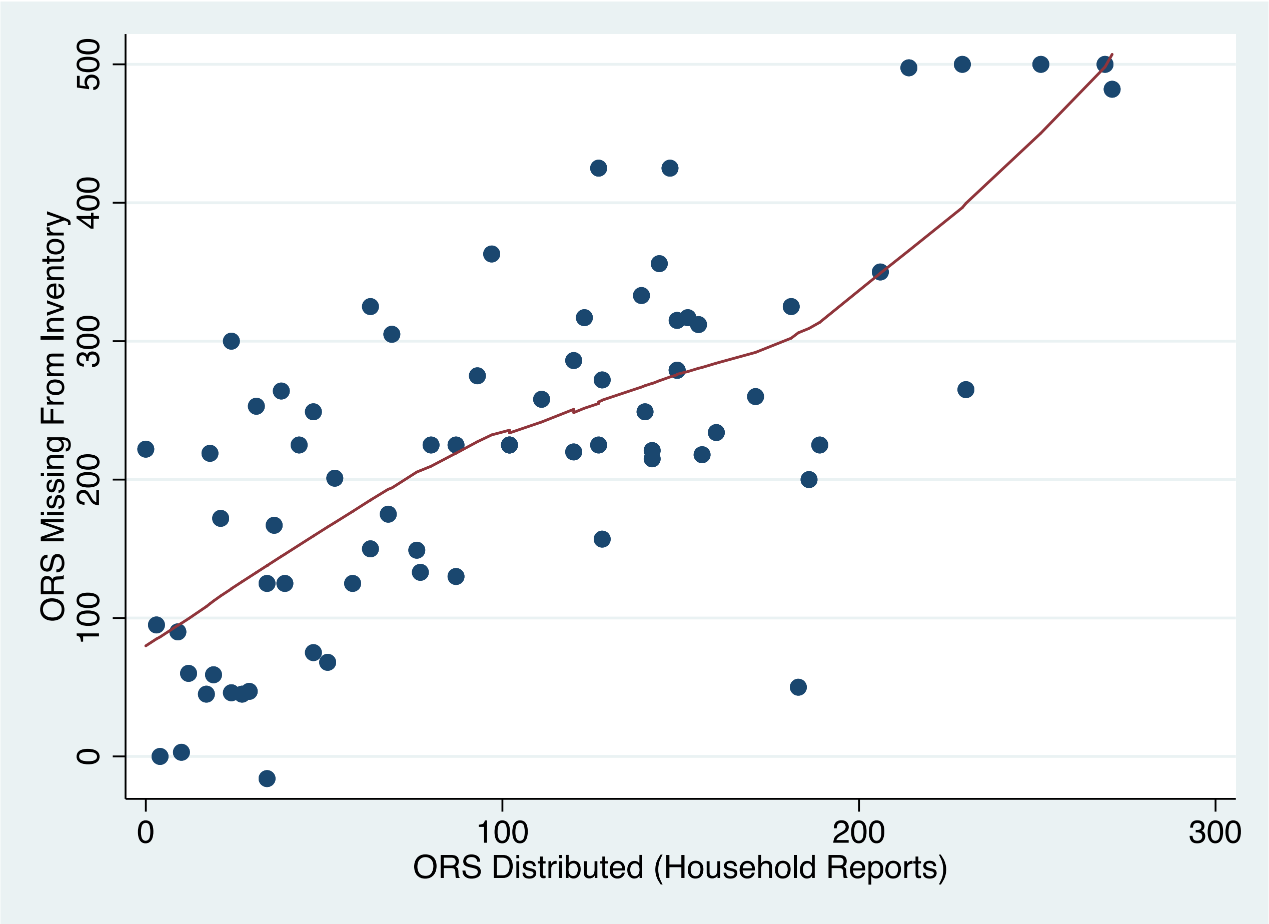

Table A: Full Regression Result from Primary Analysis in Table 3

	(1)<br>Logit Coefficients	(2)<br>Risk Differences
Treatment Effects: Relative to Control		
Free and convenient	0.927***	0.193***
	(0.163)	(0.0334)
Convenient Only	0.371**	0.0837**
	(0.152)	(0.0342)
Free Only	0.831***	0.176***
	(0.162)	(0.0339)
**Caretaker Characteristics**		
Age	0.0137**	0.00275**
	(0.00668)	(0.00133)
Number of Children	0.0457	0.00917
	(0.0384)	(0.00770)
Education: Relative to None		
Primary	-0.00505	-0.00104
	(0.206)	(0.0425)
Secondary+	0.227	0.0453
	(0.198)	(0.0403)
**Child Characteristics**		
Age	0.00397	0.000798
	(0.00332)	(0.000665)
Diarrhea Frequency: Relative to Monthly		
Every 2 months	0.109	0.0222
	(0.140)	(0.0285)
Every 3 months	0.105	0.0214
	(0.143)	(0.0291)
Every 4 months	0.137	0.0279
	(0.153)	(0.0308)
Less than every 4 months	0.183	0.0369
	(0.128)	(0.0257)
Blood in Stool	0.211	0.0424
	(0.180)	(0.0362)
Concurrent Fever	0.477***	0.0959***
	(0.114)	(0.0227)

**Households Characteristics**		
Water Source: Relative to Piped		
Protected Well	0.258*	0.0521*
	(0.145)	(0.0300)
Unprotected Source	-0.250	-0.0540
	(0.181)	(0.0391)
Main Source Of Income: Relative to Agriculture		
Public Sector	0.0818	0.0160
	(0.307)	(0.0591)
Private Sector	-0.142	-0.0288
	(0.167)	(0.0338)
Informal	0.00879	0.00174
	(0.127)	(0.0250)
Latrine Type: Relative to Covered		
Uncovered	-0.206**	-0.0414*
	(0.105)	(0.0212)
Bush	-1.001	-0.218
	(0.776)	(0.0981)
**Village Characteristics (Baseline)**		
% Visited by CHW Last 4-Weeks	0.456**	0.0917**
	(0.210)	(0.0419)
% Aware of Free ORS in Village	-0.383*	-0.0769*
	(0.230)	(0.0462)
% With ORS Stored in Home	0.118	0.0237
	(0.394)	(0.0791)
% Used ORS	-0.0224	-0.00450
	(0.320)	(0.0643)
Observations	2,356	2,356

Standard errors in parentheses
*** p<0.01, ** p<0.05, * p<0.1

Table B: Time to ORS Use After Diarrhea Onset: Cox Proportional Hazard Model

	Days to ORS (Hazard Ratios)	
	(1)	(2)
	Unadjusted	Adjusted
Free and Convenient vs. Cntl	1.653***	1.600***
	(.158)	(.14)
Free and Convenient vs. Convenient Only	1.426***	1.349***
	(.119)	(.102)
Free and Convenient vs. Free Only	1.120	1.072
	(.095)	(.08)
Controls	No	Yes
Control Mean	4.45	
Obs	2356	2356

$***p < .01, **p < .05, *p < .1$
Village Clustered SEs in parentheses
PHM=Proportional Hazard Model
Estimates from Cox PHM in columns 1 and 2 are hazard ratios
Covariates for adjusted model described in section 2
Unit of observation = case of diarrhea

Table C: Validating Self Report Using Packet Counting

	Counting Empty Packets				
	Pr(Empty Packet)			Self Report	
**Sample**	ORS=0	ORS=1	All	Pr(ORS)	Obs
Full	0.07	0.42	0.34	0.77	518
Delivery	0.13	0.51	0.47	0.88	327
Any to Show	0.20	0.63	0.57	0.87	306
Any+Delivery	0.15	0.65	0.58	0.87	262
	Fewer Packets Observed Than Obtained				
	Pr(Observed<Obtained)			Self Report	
**Sample**	ORS=0	ORS=1	All	Pr(ORS)	Obs
Full	0.20	0.92	0.75	0.76	505
Any to Show	0.36	0.90	0.83	0.87	306
Delivery	0.40	0.94	0.88	0.88	319
Delivery (Recode)	0.00	0.94	0.83	0.88	319

Pr(Empty Packet)=Probability at least 1 empty packet observed
Pr(Observed<Obtained)=Probability that fewer packets were observed than obtained
Sample identifies sub-group from Free and Convenient group used for estimates
Delivery=Household received free delivery
Any to Show=Household had at least 1 packet to show
Recode=Households that reported no ORS use are recoded to zero for counting measure
Analysis excludes 2 Free and Convenient villages where CHW did not participate
"ORS= 0,1" indicates whether the caretaker reported using ORS

Table D: Placebo Tests (Health Behaviors That Should Not Be Affected)

	(1) Malaria Treatment	(2) Unclean Water/Food	(3) Bed Net	(4) Hand Washing
Free+Convenient vs. Control	-0.041	-0.012	0.046	0.016
	(0.042)	(0.058)	(0.051)	(0.049)
Free+Convenient vs. Convenient	0.012	-0.066	0.018	-0.029
	(0.041)	(0.054)	(0.050)	(0.054)
Free+Convenient vs. Free	-0.026	-0.023	0.023	0.009
	(0.037)	(0.051)	(0.048)	(0.048)
Controls	Yes	Yes	Yes	Yes
Control Mean	0.783	0.538	0.535	0.718
Obs	1146	2141	2354	2363

$***p < 0.01, **p < 0.05, *p < 0.1$
Estimates are marginal effects from a logit regression
Village Clustered SEs in parentheses
Controls described in section 2
Malaria Treatment=child given malaria treatment in last 4 weeks (if malaria symptoms)
Unclean Water/Food=child given unclean water or food in a last 4 weeks
Bednet=child always" slept under a bed net during last 4 weeks
Hand Washing=child washed hands at least once per day in last 4 weeks
p-values not adjusted for multiple hypotheses

Table E: Impact On ORS Use (Shorter Recall Periods)

	ORS Use					
	Last 14-days		Last 7-days		Current Case	
	(1)	(2)	(3)	(4)	(5)	(6)
Free and Conven't vs. Cntrl	0.220***	0.208***	0.211***	0.199***	0.144**	0.142**
	(0.042)	(0.036)	(0.043)	(0.04)	(0.058)	(0.061)
Free and Conven't vs. Conven't Only	0.141***	0.129***	0.126**	0.113**	0.016	-0.001
	(0.036)	(0.033)	(0.037)	(0.034)	(0.062)	(0.062)
Free and Conven't vs. Free Only	0.033	0.017	0.04	0.016	-0.021	-0.03
	(0.035)	(0.031)	(0.037)	(0.033)	(0.057)	(0.057)
Controls	No	Yes	No	Yes	No	Yes
Control Mean	0.545	0.545	0.527		0.422	
Obs			1622	1622	600	600

$* * *p < 0.01, * * p < 0.05, *p < 0.1$
Estimates are marginal effects from a logit regression
Village Clustered SEs in parentheses
Controls described in section 2
7-Days implies case ended within 7 days
14-Days implies case ended within 14 days
Current case implies case ongoing at time of survey

Table F. Treatment of Child Diarrhea in 4-Weeks Following Interventions

	% of cases in last 4-weeks using each treatment			
	**Control (Cases=597)**	**Free and Convenient[a] (Cases=584)**	**Convenient Only[a] (Cases=527)**	**Free Only[a] (Cases=648)**
**Primary Outcome**				
Used ORS	56.1% (335/597)	76.7%*** (448/584)	64.4%**### (340/528)	73.7%*** (477/647)
**Secondary Outcomes[b]**				
Used ORS on Same Day as Diarrhea Onset	19.8% (118/597)	38.7%*** (226/584)	19.9%### (105/528)	31.2%*** (202/647)
Used ORS+Zinc	30.7% (183/597)	63.5%*** (371/584)	45.3%**### (239/528)	60.1%*** (389/647)
Used Antibiotics	26.3% (157/597)	19.3%* (113/584)	24.2% (128/528)	15.3%*** (99/647)
**Ex-post Outcome[c]**				
Used Zinc	37.5% (224/597)	67.0%*** (391/584)	51.9%### (274/528)	64.8%*** (419/647)

Sample includes all households with a case of diarrhea in four-weeks leading up to the interview

Percentages are unadjusted coverage of each treatment outcome within each treatment group

* * * $p < 0.01$, * * $p < 0.05$, * $p < 0.1$ test coefficients in the other three arms relative to the control group.

# # # $p < 0.01$, # # $p < 0.05$, # $p < 0.1$ test coefficients in the other two treatment arms relative to the Free and Convenient study arm.

Significance levels of differences estimated using logistic regressions with standard errors clustered by village, calculated using the Delta method[30]

[a]Exposure to interventions was incomplete (60% in Free and Convenient; 19% in Convenient Only; 42% in Free Only)

[b] Secondary outcomes were pre-specified. P-values of the three secondary outcomes were adjusted for multiple hypothesis testing using the free step down resampling method to control the false discovery rate[21]

[c]Outome not prespecified and was requested ex-post by an anonymous reviewer

Table G. Relative Risk Ratios from Logistic Regressions

	Free and Convenient Distribution Compared to Other Arms (Relative Risk Ratios)					
	Free and Convenient vs. Control		Free and Convenient vs. Control		Free and Convenient vs. Control	
	**Unadjusted**	**Unadjusted**	**Unadjusted**	**Unadjusted**	**Unadjusted**	**Unadjusted**
Used ORS (Primary Outcome)	1·37	1·34	1·19	1·18	1·04	1·03
	(1·23 - 1·50)	(1·23 - 1·46)	(1·08 - 1·30)	(1·09 - 1·28)	(0·95 - 1·13)	(0·95 - 1·11)
Used ORS on Same Day as Diarrhea Began	1·96	1·90	1·95	1·88	1·24	1·23
	(1·57 - 2·35)	(1·54 - 2·26)	(1·55 - 2·34)	(1·51 - 2·25)	(0·98 - 1·50)	(0·98 - 1·48)
Used ORS+Zinc	2·07	2·00	1·40	1·38	1·06	1·05
	(1·71 - 2·43)	(1·68 - 2·32)	(1·19 - 1·62)	(1·17 - 1·58)	(0·90 - 1·22)	(0·90 - 1·20)
Used Antibiotics	0·74	0·71	0·80	0·80	1·26	1·21
	(0·45 - 1·02)	(0·45 - 0·97)	(0·48 - 1·11)	(0·52 - 1·09)	(0·78 - 1·75)	(0·75 - 1·66)

95% confidence intervals from logistic regression in parentheses

Standard errors clustered at the village level
