# Supplementary material for: The role of price and convenience in use of oral rehydration salts to treat child diarrhea: A cluster randomized trial in Uganda
Source: PLoS Med. 2019 Jan 24;16(1):e1002734. doi: 10.1371/journal.pmed.1002734 (PMC6345441; doi:10.1371/journal.pmed.1002734)
Supplement: S1 Protocol — (PDF) [file pmed.1002734.s003.pdf]

Pre-Analysis Plan: Working With Community Health Workers to Increase Use of ORS and Zinc to Treat Child Diarrhea In Uganda

Zachary Wagner, John Bosco Asiimwe, David I. Levine, and William H. Dow

April 2, 2017

Abstract

Many cost-effective health products are underused in poor countries, although the burden from diseases that could benefit from these products remains high. Using community health workers (CHWs) to increase utilization of essential health products is a promising strategy, yet little is known about how best to structure such programs to maximize coverage (e.g. should they distribute products for free or should they charge a fee?). In this study, we measure the impact of different CHW distribution strategies (free delivery, home sales, and free upon retrieval), while simultaneously assessing the role of two barriers that could contribute to low health product utilization–price and convenience. Oral rehydration salts (ORS) and zinc, the focus of this study, are highly effective at preventing death from diarrhea, yet they are widely underused throughout sub-Saharan Africa. However, diarrhea remains the second leading cause of death for children in the region. We designed a series of novel CHW interventions in Uganda that experimentally vary the price and convenience of accessing ORS and zinc. We will use a four-armed, cluster -randomized factorial design (three intervention groups and a control group) to assess the individual and combined impact of overcoming these barriers on ORS and zinc use. A novel *preemptive home delivery* intervention will make ORS and zinc freely available inside the home when a child comes down with diarrhea (free and convenient). A *preemptive home sales* intervention will make accessing ORS and zinc convenient available at the home, but not free. Finally, a *free upon retrieval* intervention will make ORS and zinc free but not convenient. This design allows us to isolate for the price effect (free delivery vs. home sales) and the convenience effect (free delivery vs. free upon retrieval). Our primary outcome is self-reported ORS use to treat a case of child diarrhea in the past 4 weeks. Self-reported outcomes are validated with packet counting, shorter recall, and placebo tests (assessing the impact on health products that should be minimally affected by the intervention). Secondary outcomes include ORS+zinc use, antibiotic use (inappropriate for most cases), and the time it takes to start a child on proper treatment. We will also examine the role of price and convenience in targeting subsidized ORS and zinc to those that are likely to use it and to children at higher risk of death from diarrhea. The results of this study will be used to inform CHW program design and to provide insight into what other interventions are likely to effectively and efficiently increase use of ORS and zinc.

Contents

1 Introduction **5**

2 Background **8**
2.1 Overview of Policy Environment Around ORS Use 8
2.2 Provider Interventions 8
2.3 Community Interventions 9
2.4 Social Marketing Interventions 9
2.5 Summary 9

3 Conceptual Framework: The Decision to Use ORS and Intervention Mechanisms **10**

4 Conceptual Framework: Evidence of Barriers **12**
4.1 Knowledge 12
4.2 Price 13
4.3 Convenience 14
4.4 Provider Barriers and Default Treatment Options 14

5 Research Questions **15**
5.1 Primary Research Questions 15
5.2 Secondary Research Questions 15
5.3 Tertiary/Exploratory Research Questions 16

6 Research Strategy **16**
6.1 Sampling 17
6.1.1 Population and Sampling Frame 17
6.1.2 Assignment to Treatment 18
6.1.3 Statistical Power 18
6.2 Field Work 19
6.2.1 Instruments 19
6.2.2 Data Collection 19
6.2.3 Data Processing 19

7 Empirical Analysis **20**
7.1 Variables 20
7.1.1 Primary Treatment Outcome: ORS Use 20
7.1.2 Secondary Treatment Outcomes: Zinc+ORS and Antibiotic Use 20
7.1.3 Time to Treatment Initiation Outcomes 20
7.1.4 Controls/Balance Check Variables 21
7.2 Balance Checks 22
7.3 Evaluation of Intermediate Outcomes 22
7.4 Treatment Effects 22
7.4.1 Reduced form 22
7.4.2 Time to ORS Use 24
7.4.3 Impact Of Home Storage: Instrumental Variables Analysis 24
7.5 Heterogeneous Treatment Effects 25
7.5.1 Heterogeneity by ORS Access 25
7.5.2 Heterogeneity by Child Vulnerability 26
7.5.3 Super Learner for Heterogeneous Treatment Effects 27

7.6 Targeting subsidized ORS to those that will use it 27
7.7 Cost-Effectiveness 29
7.8 Standard Error Adjustment 30
7.9 Outcomes 30

8 Robustness Checks **31**
8.1 Addressing Problems With Self-Reported Outcomes 31
8.1.1 Intentional Over Reporting of ORS Use 31
8.1.2 Unintentional Misreporting Of ORS Use 32
8.2 Attrition and Changes in Group Composition 32
8.2.1 Differential Household Refusal 32
8.2.2 Differential Reporting of Diarrhea Episodes 33
8.3 Ruling out alternative mechanisms 34

9 Discussion **35**

10 Conclusion **36**

11 Research Team **36**

12 Deliverables **36**

13 Calender **36**

14 Tables **41**

15 Appendix **57**
15.1 Appendix 1: Appendix Tables 57
15.2 Appendix 2: Survey Instrument 62

List of Tables

1 Balance Between Groups (Exogenous Variables Assessed at Endline) 41
2 Balance Between Groups (Endogenous Variables Assessed at Baseline) 42
3 Intermediate Outcomes 43
4 Impact On ORS Use 44
5 Impact On Secondary Treatment Outcomes 45
6 Impact On Time to ORS Use After Diarrhea Initiation 46
7 Impact of Home Storage: 2SLS 47
8 Heterogeneous Treatment Effects: Access 48
9 Heterogeneous Treatment Effects: Child Vulnerability 49
10 Targeting ORS Subsidies (Take-Up and Use) 50
11 Targeting ORS Subsidies (Lost Packets and Non-Child Use) 51
12 Cost-Effectiveness Analysis 52
13 Impact On ORS Use (Packet Counting) 53
14 Placebo Tests (Health Behaviors That Should Not Be Affected) 54
15 Impact On ORS Use (Shorter Recall Periods) 55
16 Differential Attrition 56
17 Balance Between Groups (Summary Statistics-Exogenous Variables) 57

18 Balance Between Groups (Summary Statistics - Endogenous Variables Assessed at Baseline) 58
19 Impact On ORS Use (Full Regression Results) 59
20 Impact On Secondary Treatment Outcomes (Full Regression Results) 60
21 Impact On Time to ORS and Zinc Use (Full Regression Results) 61

1 Introduction

In many developing countries, illnesses for which we have long had prevention technologies and effective treatments available remain leading causes of death (e.g. diarrhea and bacterial pneumonia). As a result, one of the defining challenges for the global health community is to understand 1) *why* effective health products are underused and 2) *how* to increase use. One potential barrier to take-up of effective health products is price. Although it is often argued that cost-sharing is necessary to effectively target products to those who will use them (PSI, 2003), positive prices might also contribute to low utilization. Several recent randomized controlled trials (RCTs) have demonstrated such an effect, showing that even highly subsidized prices lead to large reductions in demand and subsequent use (see Kremer et al. (2011a) for a review). Another, potentially important barrier to health product take-up is convenience of access (distance and hassle costs). Several studies have found that demand for health products is sensitive to the time required to access them, suggesting that making health products more convenient and reducing hassle costs could increase demand (Thornton, 2008; Banerjee et al., 2010; Kremer et al., 2011b).

In this study, we will measure the role of price and convenience in the context of treatment for child diarrhea in Uganda. Diarrheal diseases are the second leading cause of death globally for children under five-years-old, with roughly 500,000 deaths annually (Liu et al., 2015). In Uganda, 90 in every 1000 live births die before their 5th birthday (UDHS, 2011) and diarrheal illnesses account for about 13% of these deaths (Liu et al., 2012). Diarrhea death is particularly tragic since roughly 93% of deaths could be averted with the use of oral rehydration salts (ORS) (Munos et al., 2010). ORS effectively treats diarrhea induced dehydration, which is the underlying cause of most diarrhea deaths (Cash et al., 1970; Pierce et al., 1969; Santosham, 1982; Spandorfer et al., 2005). In 1978, ORS was lauded as one of the most important medical advances of the 20th century by the medical journal *The Lancet* (Lancet, 1978) and since 1980, when ORS became widely available, there has been more than a two-thirds reduction in global deaths from diarrhea for children under five-years-old (Victora et al., 2000; Liu et al., 2012). Due to its low cost and high effectiveness, ORS is recommended by the WHO for all cases of child diarrhea regardless of illness severity (USAID, 2005). More recently, zinc was introduced as a recommended treatment for child diarrhea to compliment ORS after it was demonstrated to reduce illness severity and provide short term prevention benefits (Bhutta et al., 2000).

Despite the effectiveness of ORS and zinc in preventing death from diarrhea, utilization remains dangerously low, particularly in sub-Saharan Africa (SSA) (Forsberg et al., 2007; Pantenburg et al., 2012; Ram et al., 2008; Santosham et al., 2010; Liu et al., 2012; Sood and Wagner, 2013). In Uganda, the location of the proposed study, only about 46% of diarrhea cases are treated with ORS (UDHS, 2011). Finding ways of increasing use of ORS is an essential step towards reducing child mortality in Uganda and throughout the region.

Although there is an extensive body of medical literature assessing the health gains from ORS (Cash et al., 1970; Pierce et al., 1969; Santosham, 1982; Spandorfer et al., 2005; Munos et al., 2010) and identifying the problem of underuse (Forsberg et al., 2007; Pantenburg et al., 2012; Ram et al., 2008; Santosham et al., 2010; Liu et al., 2012; Sood and Wagner, 2013) there is little evidence on *why* ORS use remains low and what interventions could be used to increase use. A recent systematic review by (Lenters et al., 2013) found only 19 studies that assessed interventions to increase ORS use, and only 3 RCTs. Nearly all interventions were some form of social marketing and studies were skewed geographically towards South Asia. The authors concluded that most of the studies reviewed were of low quality and as a result much more evidence is needed on potential strategies for increasing ORS use, particularly in SSA.

There are several potential explanations for why ORS use remains low. First, it is possible that people are unaware of the life-saving benefits of ORS (the *information barrier*). However, this is unlikely to be an important barrier since ORS has been widely available and socially marketed for over 3 decades, and awareness in Uganda is nearly universal (UDHS, 2011). As a result, we do not directly measure the role of information in this study.

Second, although ORS is free at public health clinics, over half of caretakers seek care for diarrhea in the private sector, where they are required to pay for ORS (UDHS, 2011). Moreover, many community health workers in Uganda sell ORS at a subsidized price. In our study villages, over 70% of caretakers that used ORS, paid for it. Since ORS does not provide an *observable* benefit to the child (no effect on volume or duration of diarrhea), caregivers might undervalue ORS and might not be willing to pay the small price (USD $0.30 per treatment course) (the *price barrier*). Moreover, caregivers in poor communities might be liquidity and credit constrained, and thus might not have the cash-on-hand to pay the small fee.

Third, it can be an inconvenience to visit health facilities or drug shops to retrieve ORS, particularly since most children have diarrhea many times throughout the year. Many caretakers in Uganda are required to walk long distances to retrieve ORS. Even for those living in relatively close proximity to ORS distributors, cognitive biases such as time-inconsistent preferences, inertia, or limited attention could hinder ORS retrieval (the *convenience barrier*).

Fourth, even conditional on a visiting a provider for treatment, many providers fail to provide ORS and zinc when presented with a case of child diarrhea (Sood and Wagner, 2013; Mohanan et al., 2015) (the *provider provision barrier).* In Uganda, only 50% of children who visit a health provider receive ORS and under 10% receive zinc (UDHS, 2011). Providers, particularly in the private sector, often distribute antibiotics without ORS. However, antibiotics do not treat dehydration, the reason for nearly all deaths. Moreover, most cases of child diarrhea in sub-Saharan Africa are viral, which means antibiotic provision often contributes to antibiotic resistance without providing any benefits.

We designed a series of interventions that experimentally vary the price and convenience of accessing ORS and zinc. We will use a four-armed, cluster randomized factorial design (three intervention groups and a control group) to assess the relative and combined impact of overcoming these barriers on ORS use (primary outcome). We will work with Community Health Promoters (CHPs), a program supported by BRAC Uganda, to carry out the interventions. A novel *preemptive home delivery* intervention will make ORS and zinc freely available inside the home when a child comes down with diarrhea (free and convenient). Under this intervention, all households with a child under 5-years-old will receive a free delivery of ORS and zinc from the CHP to store in their homes at the beginning of the study. Having ORS convenient in the home will also addresses the provider provision barrier since caretakers will be able to forgo seeking treatment outside of the home. A *preemptive home sales* intervention will make accessing ORS convenient but not free. Under this intervention, households with a child under 5-years-old will receive a household visit at the beginning of the study with an offer to sell ORS and zinc at a subsidized price. Finally, a *free upon retrieval* intervention will make ORS and zinc free but not convenient. Under this intervention, all households with a child under 5-years-old will receive a household visit from the CHP informing them that they can retrieve free ORS and zinc from the CHP's home. This experimental design allows us to evaluate the impact of a novel approach to utilizing community health workers to encourage ORS use as well to isolate for the role of price and convenience as barriers to ORS use.

This analysis contributes to the growing body of economics literature assessing the role of price and convenience in demand for health products in developing countries. Although sev-

eral economists have demonstrated that price and convenience are important barriers that contribute to low use of other health technology, including clean water products to *prevent* diarrhea (Ashraf et al., 2010; Dupas et al., 2016; Kremer et al., 2011b), it remains unclear to what extent these barriers contribute to low ORS use to *treat* diarrhea.

In addition the primary analysis described above, we will conduct several additional analyses investigating other important results of these interventions. First, in addition to measuring the impact of these interventions on ORS and zinc use, we will also investigate how they affect time to treatment initiation. Death can occur very quickly after the initiation a diarrhea episode, and the world health organization (WHO) recommends immediate initiation of both ORS and zinc.

Second, in addition to understanding the impact of these CHW interventions, a related question is how *home ORS storage* impacts ORS use. If home storage significantly increases ORS use, other programs could also focus on ensuring households have ORS stocked (e.g. maternal and child health clinics). Since some CHWs may not carry out the intervention correctly and some caretakers might lose the ORS after the delivery, not all households who receive free deliveries will have ORS stored in the home when the child becomes ill. Moreover, some households that do not receive free deliveries *will* have ORS stored in the home when the child becomes ill. To measure the impact of home ORS storage on use, we will use random group assignment as an instrument for having ORS stored in the home.

Third, we will investigate the role of price and convenience in targeting the most vulnerable cases of diarrhea (very young children and more severe episodes). It has been argued that cost-sharing for health products better targets the most vulnerable beneficiaries, whereas free distribution could lead to expanded coverage among those with less need. It is possible that free ORS delivery expands coverage to less vulnerable children (children with little mortality risk). Several other studies have assessed the role of price in targeting health products to those most likely to benefit and there is little evidence that free-distribution expands coverage to those with less need. Kremer and Miguel (2007) find that parents of children with higher levels of parasitic worms are no more likely to purchase deworming treatment. Cohen and Dupas (2010) find that pregnant women who are anemic (a sign of a prior malaria case) are no more likely to purchase a mosquito net than non-anemic women. Ashraf et al. (2010) and Kremer et al. (2011c) find that households with young children (who more vulnerable to death from diarrhea) are not willing to pay more for point-of-use water treatment. Our work is the first to assess the role of both prices and hassle costs in terms of targeting subsidized diarrhea treatment to the most vulnerable.

Finally, we will also investigate whether free delivery does a worse job of targeting ORS to people with a higher propensity to use it than hassle costs or home sales. It is often argued that charging for health products does a better job at targeting subsidies to people that will use the products (PSI, 2003). Although several studies have shown that charging for health products reduces both demand and coverage, there is mixed evidence on how price affects targeting products to those most likely to use them (Cohen and Dupas, 2010; Ashraf et al., 2010; Kremer et al., 2011a). Moreover, very few studies have directly compared monetary prices, hassle costs, and free delivery in terms of efficient allocation of subsidies (Dupas et al., 2016), and this work will be the first to do so in the context of diarrhea treatment. Moreover, we will compliment our resource targeting analysis with a cost-effectiveness analysis, which will provide a more complete picture of efficient resource allocation.

The rest of this paper proceeds as follows. Section 2 discusses the background on ORS use and the recent evidence on what works to increase ORS use, sections 3 and 4 provide a conceptual

framework that highlights the mechanisms through which our interventions can be expected to increase ORS use, section 5 outlines our research questions, section 6 describes our research design and strategy, section 7 describes our empirical analysis, section 8 describes robustness checks and validity tests to compliment our main analysis, section 9 discusses how our findings will contribute to the existing literature, and section 10 concludes.

2 Background

2.1 Overview of Policy Environment Around ORS Use

Although most developing country governments and international aid organizations include expansion of ORS coverage as a stated goal, there is little evidence on what interventions are effective at doing so. There were substantial efforts to increase ORS use in the 1980s and 1990s, and over 100 countries had ORS promotion programs in place by 1988 (Organization et al., 1990). These programs appear to have been successful, increasing use of ORS or other forms of oral rehydration therapy (ORT) from close to 0 in 1980 to around 40% in 1990 (Forsberg et al., 2007). Moreover, awareness of ORS was nearly universal. However, most programs aimed at increasing ORS use were comprised of many different interventions (e.g. provider training, social marketing, supply chain management, etc.) making isolation for the impact of each mechanism difficult. Moreover, after the big push to increase ORS use during the 1980s and 1990s, the share of diarrhea cases that are treated with ORS has leveled off at around 40%, suggesting that novel interventions are needed to overcome this "last mile" problem.

In Uganda, the ministry of health (MoH) and other international organizations recognize the need for intervention and have programs in place aimed at increasing ORS use. In 2001, the MoH started the Village Health Team project, where community members are assigned to act as a liaison between rural areas and the health system by providing basic health care needs including ORS distribution and diarrhea education. The Clinton Health Access Initiative (CHAI) in Uganda focuses on reducing the price of ORS and zinc in the private sector, where many people seek treatment. USAID funds the Strengthening Health Outcomes through the Private Sector (SHOPS) project, which focuses on increasing provision of ORS and zinc in the private sector. Living Goods and BRAC both have CHP programs which focus on increasing knowledge of and access to ORS by having community members sell the products at a subsidized price. Plan International focuses on ensuring sufficient supply of ORS and zinc in rural areas. Although there is an immense amount of effort being put towards many different interventions aimed at increasing ORS use, it is not clear what the remaining barriers to ORS are and which interventions are likely be effective. Below, we outline the evidence in the 3 areas where most of the recent empirical research has focused.

2.2 Provider Interventions

There is substantial evidence demonstrating that health providers, particularly in the private sector, fail to provide ORS when presented with a case of diarrhea (Sood and Wagner, 2013; Wagner et al., 2014; Mohanan et al., 2015). However, there is little evidence demonstrating why such under-provision in the private sector occurs. Wagner et al. (2014) find that private providers in India are less likely to directly distribute ORS and suggest that making ORS more convenient to private sector patients could increase take-up. Friedman et al. (2015) randomly assigned drug shop sellers in Ghana to receive text messages encouraging ORS provision.

Although drug sellers who received the messages reported increased ORS provision, their observed ORS provision practices did not change. Clearly, much more work is needed in order to understand why private providers underprovide ORS and how to increase provision. However, many caretakers (potentially the most vulnerable) do not seek care from a provider at all and therefore would not benefit from provider focused interventions.

2.3 Community Interventions

Several community interventions have shown to be successful at increasing ORS use. In a recent cluster RCT in Myanmar, Aung et al. (2014) find that a social franchising intervention that provided community education and community supply of ORS and zinc increased ORS and zinc use from 1.8% to 13.7%. Awor et al. (2014) use a quasi-experimental design to evaluate an integrated community case management (ICCM) intervention in Uganda that trained private drug shops, provided supply of ORS, and provided education to community members. They found that provision of ORS and zinc increased 12-fold as a result of the intervention. An unpublished study that experimentally evaluated the impact of the Living Goods and BRAC CHP program (the same program that will carry out our intervention) found that ORS use increased from 33% to 39% as a result of the CHP program.

There is also evidence that introduction and promotion of zinc in a community as a compliment to ORS results in increased ORS use (Lenters et al., 2013). Baqui et al. (2004) randomly assigned introduction of zinc to communities in Bangladesh and found that access to zinc increased use of ORS. Bhandari et al. (2008) find similar results in India.

2.4 Social Marketing Interventions

There are several observational studies that assess the impact of social marketing and mass media campaigns on ORS use. Kassegne et al. (2011) found that ORS use increased from 20% to 30% after a PSI sponsored social marketing campaign in Berundi. Rao et al. (1998) found that ORS use in India during a time when the government promoted ORS through mass media increased more for mothers that had exposure to a radio, television, or cinema. Lenters et al. (2013) reviewed several studies in a meta-analysis assessing the impact of social marketing and mass media campaigns on ORS use and found a pooled risk ratio of 2.05, although this estimate was not statistically significant.

2.5 Summary

Increasing ORS use appears to be an important part of many national health agendas, yet we know very little about what effectively achieves this goal. Provider interventions appear to have potential, although the evidence is lacking. Community and social marketing interventions have shown to be effective, but neither appear to achieve the desired coverage rates, which suggest they alone are not sufficient. Not only will the current study evaluate the impact of a novel intervention to increase ORS use, but it will also isolate for the mechanisms at work, which will help guide future ORS promotion interventions. Next, we provide a conceptual framework for the mechanisms and specific channels through which each intervention is expected to work.

3 Conceptual Framework: The Decision to Use ORS and Intervention Mechanisms

Each of our interventions are likely to affect ORS use through different channels. Figure 1 displays a diagram of the caretaker's choice to use ORS to treat their childs diarrhea and the various barriers that she faces at each stage. The child starts off healthy and during this time, caretakers can either acquire ORS for later use (preemptive take-up) or not acquire ORS. The decision to acquire ORS for later use has many potential barriers including financial barriers (prices and liquidity/credit constraints), convenience barriers (distance to provider, limited time, and limited attention or mental bandwidth), knowledge barriers, and other barriers not addressed by this study (e.g. low perceived probability diarrhea, preferences for other treatments, and cultural barriers). If the caretaker decides to take-up ORS pre-emptively and the child becomes ill with diarrhea, then most caretakers will have ORS stored at home upon diarrhea initiation (although some could lose or give away the product). With ORS stored at home when diarrhea initiates, then the choice to use ORS is fairly easy, only impeded by barriers unrelated to price and convenience (i.e.knowledge, low perceived severity of illness and "other" barriers described above).

On the other hand, if caretakers do not preemptively acquire ORS and the child comes down with diarrhea, then they have to make a series of complex choices and face an array of potential barriers after the child comes down with diarrhea before acquiring and using ORS. First, they choose whether/where to seek treatment for the child. For, simplicity, we assume that caretakers can either seek treatment from a CHP, another provider, or choose not to seek treatment. Seeking treatment after a diarrhea initiation has the same barriers as preemptive take-up, except that at this point the child might be in danger, and time is an issue. If the caretaker decides to seek treatment, receipt of ORS is not guaranteed and is subject to provider barriers (provider recommendation and supply), as well as financial and knowledge barriers. As mentioned above, many providers in Uganda do not provide ORS when presented with diarrhea. If the caretaker does received ORS from the provider, she then has the choice of using the ORS to treat the child.

This diagrams highlights an important point. Preemptive take-up of ORS makes the decision to use ORS when the child comes down with diarrhea much less complicated with far fewer barriers than if ORS is acquired after diarrhea initiation. Caretakers that do not take-up ORS pre-emptively have to make several complex decisions and face many barriers to ORS take-up and use after the becomes ille.g. the caretaker could avoid treatment because the provider is too far away or they are busy with other activities, the provider could recommend an antibiotic instead of ORS or they could have a stock out. By preemptively acquiring ORS, the caretaker bypasses barriers to seeking treatment once the child becomes ill and barriers to receiving ORS from a provider.

Each of our interventions will alter the likely pathway taken by the caretaker in different ways by addressing a different set of barriers (although all interventions will address the knowledge barrier).

Free preemptive home-delivery with information
The effected pathway for free preemptive home-delivery is indicated with a green dotted line and the barriers addressed are indicated by * in figure 1. This intervention will increase the likelihood of preemptive ORS take-up to nearly 100%. Financial barriers, convenience barriers, and knowledge barriers will all be addressed. Since nearly all households will have ORS stored at home when the child comes down with diarrhea, ORS use is only hindered by barriers

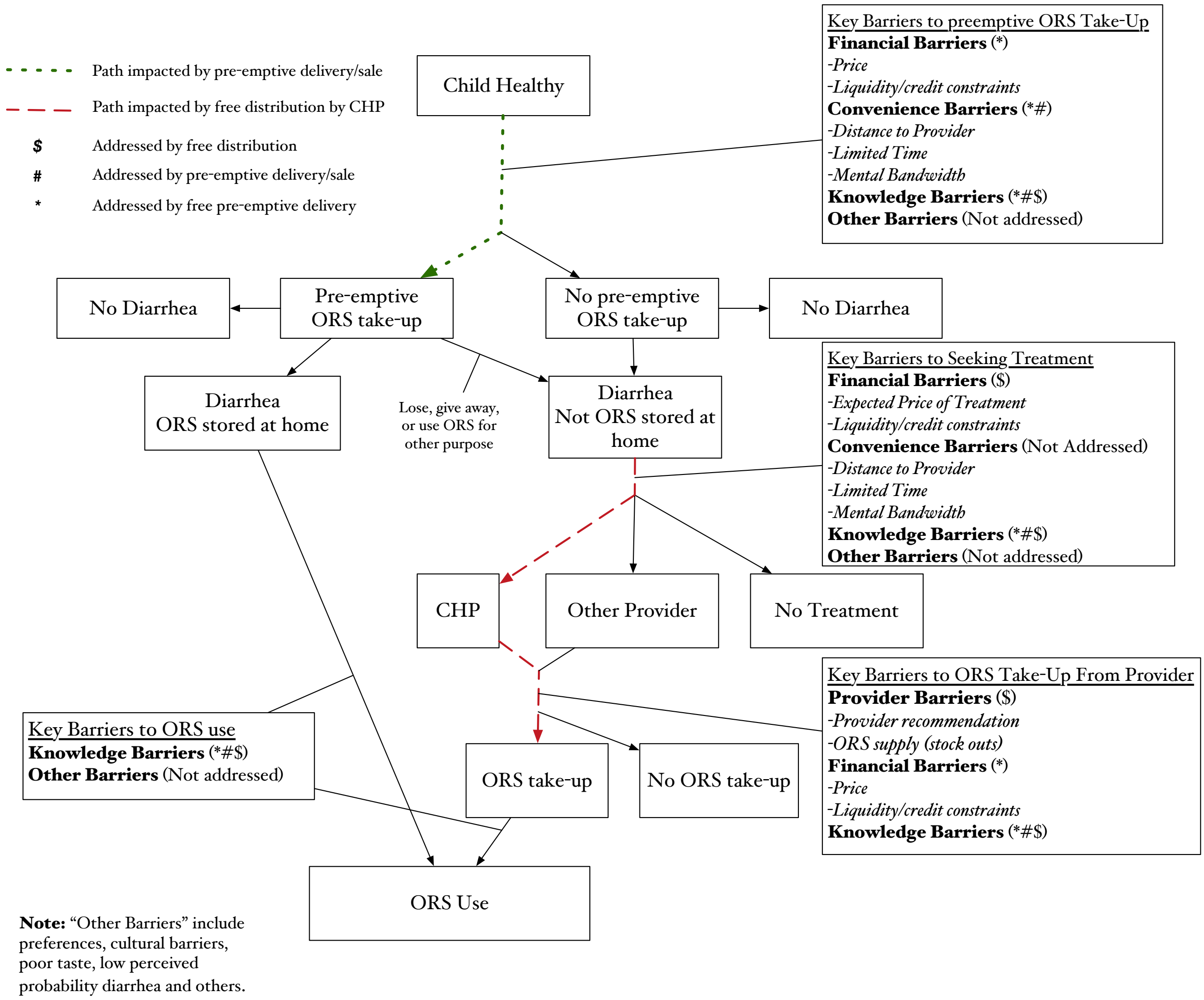

Figure 1: Flow diagram of ORS take-up and use

unrelated to price, convenience, and knowledge.

Preemptive home sales with information
Preemptive home visits with an offer to sell ORS will alter the average caretaker's preferred path in a similar way as free delivery (also indicated by a green dotted line), however it will not overcome all of the same barriers (indicated by # in figure 1). Only convenience and knowledge barriers are addressed, leaving financial barriers as impediments. Therefore, the extent to which financial barriers impact preemptive ORS take-up can be measured by taking the difference between the home delivery group and the home sales group.

Free distribution upon retrieval from CHPs home with information
Free distribution of ORS upon retrieval from the CHPs home will only affect the decision making process after the child comes down with diarrhea. The effected pathway for this intervention is indicated with a red dashed line. This intervention will affect ORS take-up in two ways. First, the caretaker will have been informed that the CHP has free ORS available, which will shift the provider decision pathway towards seeking treatment from the CHP. This will also address financial barriers to seeking treatment, although it will not address convenience barriers. Second, upon seeking treatment from the CHP, financial barriers will no longer be present since ORS will be provided for free. Moreover, the CHP does not provide other treatments aside from ORS and zinc, and our intervention will ensure she is fully stocked, addressing the provider barriers, since nearly all mothers that seek treatment from the CHP will received ORS. This shifts the distribution of treatment seeking towards a provider with higher probability of providing ORS. The only difference between free preemptive delivery and free distribution upon retrieval is the effect of convenience.

4 Conceptual Framework: Evidence of Barriers

The barriers to ORS use that are addressed by our interventions are all related to either poor knowledge, price, convenience, or provider barriers. Although the evidence for some of these barriers is limited in the context of ORS use, there has been a substantial amount of work identifying and addressing these barriers in the context of other health products. Below we highlight the evidence for each of these barriers and how the evidence relates to ORS.

4.1 Knowledge

Potentially the most frequently offered explanation for low ORS use is that caretakers are unaware of the product or its benefits. This suggests that informing caretakers of the life saving benefits of ORS would be effective at increasing take-up. Providing information about healthy behaviors has had success in terms of behavior change in the past (Dupas, 2011; Kremer et al., 2011a) and this thinking has led to many social marketing campaigns aimed at spreading awareness of ORS (Lenters et al., 2013; Kassegne et al., 2011). Mass information campaigns in the 1980s are often credited with the high ORS usage rates in Bangladesh (Smillie, 2009) and the large reduction in diarrhea mortality in Egypt (Levine, 2004). Moreover, for the last 3 decades their has been a concerted effort to increase awareness of ORS to treat child diarrhea in Uganda and knowledge generation about proper diarrhea treatment is a key role of CHPs. It appears that this effort has been very effective, evidenced by near universal awareness of ORS across the country. In the most recent Demographic and Health Survey, over 90% of mothers of children under-5 were aware of ORS (UDHS, 2011). Similarly, among the population of the present study, where CHPs have already been working to increase ORS knowledge, over

96% of mothers had heard of ORS (baseline survey). Moreover, over 85% of the mothers in our sample had used ORS to treat diarrhea at some point in the past. However, this knowledge of ORS does not seem to translate into sufficient use as only 46% of diarrhea cases in the 2 weeks prior to data collection were treated with ORS (UDHS, 2011). Two points emerge from this discussion. First, awareness of ORS is reaching a ceiling and there is little room for increased awareness. Second, awareness of ORS is not enough to results in sufficient ORS use. Therefore, in order for information to affect ORS use, it must be provided in a strategic way that changes preferences or beliefs about ORS, a much more difficult task then simply raising awareness about the product. In this study, CHPs will reinforce ORS and zinc knowledge, however, most households will already have received this information. We expect the information provide through our study to have little affect on ORS use and we do not test for this effect directly.

4.2 Price

Another potential reason for under-use of ORS is unwillingness-to-pay even the small, often subsidized, price. Although ORS is freely available at public health clinics, most caretakers seek care in the private sector where they are required to purchase ORS. Moreover, many community health workers (including BRAC's CHPs) offer ORS at a subsidized price.

Several recent RCTs show that even highly subsidized prices can result in a substantial reduction in take-up and use of preventive health products relative to free-distribution. Kremer and Miguel (2007) found that free distribution of deworming medication to Kenyan children increased take-up from 18-75% relative to a small fee. Cohen and Dupas (2010) found that take-up of bed nets in Kenya falls by 60% when the price increases from 0 to $0.60. Ashraf et al. (2010) found that take-up of point-of-use water treatment in Zambia falls by 30% when price increases from $0.09 to $0.25. Similarly, Dupas et al. (2016) found that take-up of point-of-use water treatment in Kenya falls by 38% and use falls by 62% when the price increases from zero to a 50% discount. Kremer et al. (2011c) found that a majority of households use chlorine for water treatment in Kenya when provided for free, but only 10% use it at the market rate. Dupas et al. (2011) found that chlorine use increased nearly 3 fold when it was provided for free relative to a 50% discount. Spears (2009) found that take-up of hand washing soap in India falls from 84% to 13% when the price changes from 3-15 rupees. Taken together, these studies suggest that poor people in developing countries are very sensitive to prices of health products, and even highly subsidized prices can substantially reduce take-up and use.

Although people appear to be extremely sensitive to prices of preventive products, demand for remedial health products appears to be relatively price-inelastic. For example, Cohen and Dupas (2010) show that increasing the price of an antimalarial treatment course for young children by 250%, from US$0.30 to $1.5, does not reduce the share of households buying the treatment (about 32%). This discrepancy in price sensitivity for curative products and preventive products is often explained using concepts from behavioral economics such as present bias; the benefits from curative products pay off immediately whereas the benefits of preventive products, although a smart investment with high returns, pay off far into the future. Since ORS is only recommended once a child becomes ill with diarrhea, it could be thought of as remedial. Therefore, it is possible there is less price sensitivity than found in the above studies which focused on primary prevention products. However, ORS could also be thought of as secondary prevention (managing an illness to avoid poor outcomes) instead of curative and has similar features as primary prevention products from past studies. First, ORS has limited observable effects on the main diarrhea symptoms (i.e. volume and duration of episode), and instead

treats dehydration to prevent death. Therefore, similar to primary preventive products, the benefits of ORS (keeping the child alive and hydrated) might go unnoticed since the diarrhea persists. On the other hand, malaria treatment directly affects the main symptoms of malaria. Second, ORS initiation is recommended immediately after the diarrhea episode begins, prior to the child becoming dehydrated. Therefore ORS is *actually* recommended as prevention of dehydration. Finally and most importantly, similar to preventive products ORS use remains low although there appear to be substantial returns to investment.

It remains unclear if ORS will fall more in line with preventive or curative products in terms of price sensitivity. There is only poor evidence on how sensitive caretakers are to the price of ORS, and no experimental evidence. Aung et al. (2013), using a survey in Myanmar, find that less than 25% of caretakers are willing to pay the market rate for ORS. Several other studies have documented the impact of community based interventions to increase ORS use, some of which include free distribution (see Das et al. (2013) for a review), however, no studies have isolated for the impact of ORS price. The fact that ORS use remains low although it is widely available, low cost, and extremely effective suggests that caretakers are sensitive to ORS price. Therefore, we expect that free provision of ORS will result in increased coverage.

4.3 Convenience

A third potential barrier to ORS use is convenience of access or hassle costs. Many mothers are required to walk long distances or pay high transport costs to reach their nearest clinic. Time constraints may limit caregivers to rationally choose to only make the long journey if a case becomes "severe", at which point it could be too late. Even when access points are easily accessible, concepts from behavioral economics such as time-inconsistent preferences, inertia, or limited attention could hinder ORS retrieval. For example, mothers may have a preference for retrieval of ORS in a future time period since they are informed of the best practice, but when their child becomes ill in the current period, their preferences are different or a competing task occupies their mental space and they choose not to travel to retrieve ORS.

Several studies suggest that distance and inconvenience can be important barriers to take-up. Thornton (2008) found that distance to HIV testing centers was a key barrier, an even larger barrier than price, to retrieval of HIV test results in Malawi. Kremer et al. (2011b) found that individuals are only willing to walk 3.5 minutes further to collect water from a protected spring that produced clean water as opposed to retrieving contaminated water from an unprotected well. Banerjee et al. (2010) found that small incentives (less than a days wage) resulted in much greater willingness to travel to immunization camps. Taken together, these studies demonstrate that distance and convenience are important factors in take-up of health services, and that making products more convenient or nudging people to overcoming cognitive biases could increase utilization.

Although there is no direct evidence on how convenience of ORS affects use, several studies find that community interventions that increase ORS availability improved coverage (Das et al., 2013). However, other factors associated with community distribution could be driving these effects.

4.4 Provider Barriers and Default Treatment Options

Even if caretakers travel the long distance to a faraway health provider or overcome the inertia to visit a more convenient provider, they are faced with several treatment choices in addition to

ORS and zinc. Often treatment choices are left to the provider's discretion and although most providers are aware that treatment guidelines include ORS, they frequently provide alternatives such as antibiotics or antidiarrheals instead, both of which are often unnecessary and potentially harmful (Sood and Wagner, 2013; Mohanan et al., 2015). In 2011, Only 50% of children in Uganda who visited a health provider for diarrhea care received ORS and under 10% received zinc (UDHS, 2011). Although there is limited evidences to help understand why providers fail to give caretakers ORS, it is often conjectured that private providers have a preference for selling higher cost products. Directly providing ORS and zinc to households for storage and making ORS and zinc freely available may have the effect of making these treatments the default choice. Having ORS delivered and stored in the household or freely available from the CHP will eliminate the need to visit a provider for treatment where other products that don't address dehydration are likely to be given in place of ORS. There is a substantial literature demonstrating the power of defaults (White and Dow, 2015; DellaVigna, 2009), and we expect that making ORS and zinc the default choice will both increase use of ORS and zinc and reduce unnecessary and potentially harmful use of antibiotics.

5 Research Questions

5.1 Primary Research Questions

Primary Question 1: Does *preemptive*[1] *home delivery* with *free distribution* of ORS and zinc coupled with information about the importance of proper treatment result in greater use of ORS to treat child diarrhea relative to the status quo?

Primary Question 2 (Price Effect): Does preemptive home delivery with *free distribution* of ORS and zinc result in greater use of ORS to treat child diarrhea relative to preemptive home visits with *offers to sell* the products?

Primary Question 3 (Convenience Effect): Does free distribution with *preemptive delivery* of ORS and zinc for household storage result in greater use of ORS than free distribution upon *retrieval* from the CHP's home?

5.2 Secondary Research Questions

Secondary Question 1: Among those with free access to ORS, does having ORS stored in the home when a child comes down with diarrhea result in greater ORS use than not having ORS stored at home?

Secondary Question 2: Do *preemptive home visits* with an *offer to sell* ORS and zinc at the typical subsidized price currently charged by CHPs (roughly USD$0.30 per treatment course) coupled with information about the importance of proper treatment result in greater use of ORS to treat child diarrhea relative to the status quo?

Secondary Question 3: Does *free distribution* of ORS and zinc upon *retrieval by caretakers* from the CHP's home coupled with information result in greater use of ORS to treat child diarrhea relative to the status quo?

[1] "preemptive" implies prior to the occurrence of a diarrhea episode

Secondary Questions 4-6: Same as primary question but assessed for ORS and zinc combined.

Secondary Questions 7-9: Same as primary questions but assessed for time to ORS use after diarrhea initiation?

Secondary Questions 10-11: Same as secondary questions 2-3 but assessed for ORS and zinc combined.

Secondary Question 12: Does having ORS stored in the home when a child comes down with diarrhea result in less time between diarrhea initiation and ORS use than not having ORS stored at home?

Secondary Questions 13-15: Do these interventions reduce antibiotic use?

5.3 Tertiary/Exploratory Research Questions

Tertiary Question 1: Does *free distribution* of ORS and zinc upon *retrieval by caretakers* result in greater take-up and use of ORS relative to *preemptive home visits* with an *offer to sell* the products?

Tertiary Questions 2-4: Same as primary questions bus assessed for ORS take-up (probability of obtaining ORS).

Tertiary Question 5: How do these interventions compare in terms of targeting ORS to those that will use it?

Tertiary Questions 6: What is the impact of these interventions on *time between diarrhea initiation and zinc initiation*?

Tertiary Questions 7: Does free delivery of ORS do a worse job of targeting the most vulnerable children (youngest and most severe cases) than imposing hassle costs or prices?

Tertiary Questions 8: Does free delivery of ORS have a larger effect for households with the least access to ORS distributors?

6 Research Strategy

This project will use a cluster randomized controlled trial design. We will work with BRAC to select 120 villages (see sample size calculations below) where their CHP program is active (CHPs are active in over 2000 villages in Uganda). CHPs are community members who are hired by BRAC to sell essential health products to others in the village, which are purchased by CHPs from BRAC at a subsidized price. CHPs are also trained to provide very basic primary care and health education. The interventions will take place at the village level since one CHP is dedicated to serve an entire village. Each village will be randomly assigned to one of four groups.

Group 1 — Control: No intervention will take place. Caretakers will have standard access to ORS and zinc at local health facilities and pharmacies. Some CHPs in control villages could make household visits, however offers to sell diarrhea treatment pre-emptively are rare and CHPs are generally not the source of diarrhea treatment.

Group 2 — Household Visit + Free Distribution + Preemptive Delivery: CHPs will be provided a small incentive to visit all of the households in their catchment area that contain a child under 5-years-old (roughly 100 households) at the beginning of the study. CHPs will train caretakers on the dangers of diarrhea and the importance of ORS and zinc use. CHPs will then offer to give ORS and zinc to caretakers for free to store in their homes.

Group 3 — Household Visit + Cost Sharing + Preemptive Delivery: CHPs will be provided a small incentive to visit all of the households in their catchment area that contain a child under 5-years-old at the beginning of the study. CHPs will train caretakers on the dangers of diarrhea and the importance of ORS and zinc use. CHPs will then offer to sell ORS and zinc to caretakers at their standard subsidized price (roughly USD$0.30 per treatment course) to store in their homes.

Group 4 – Household Visit + Free Distribution Upon Retrieval: CHPs will be provided a small incentive to visit all of the households in their catchment area that contain a child under 5-years-old at the beginning of the study. CHPs will train caretakers on the dangers of diarrhea and the importance of ORS and zinc use. CHPs will then inform caretakers that they have ORS and zinc available for free that caretakers can retrieved from the CHPs home. The average distance to the CHPs household is about 15 minutes.

6.1 Sampling

6.1.1 Population and Sampling Frame

We will use six of BRAC's microfinance branches as our study sites (BRAC has 128 branches throughout the country). BRAC's "branches" are local offices which are used to administer their programs to the surrounding villages. Each branch corresponds to 20 CHPs resulting in 120 villages/CHPs in total to be included in our sample. All villages within selected branches will be enrolled in the study and randomized to one of the four groups described above. Branches were chosen based on 3 criteria: 1) high diarrhea prevalence, 2) branch managers are willing to participate and help with coordination, and 3) close proximity to Kampala (due to budgetary constraints).

Once branches and villages are selected, the study team will enroll 80 households with a child under 5-years-old in each village. Although most villages have 100+ households, we do not gain much power from including additional households in each cluster as power is driven mostly by the number of villages, and logistical constraints limit our ability to do a full census. In order to draw our sample of households, enumerators will start at the CHP's household (where her operations take place) and visit to the 40 nearest households with a child under-5 during the baseline survey and the 80 nearest households with a child under-5 during the endline survey. Although our sample might not be representative of the entire village population, it will be representative of the households most likely to benefit from the intervention.

Although we will enroll 40 and 80 households per villages at baseline and endline, respectively, only households with at least one child who was reported to have had a case of diarrhea in the past 4 weeks (during baseline or endline) will be included in the analysis (estimated sample size and power described below).

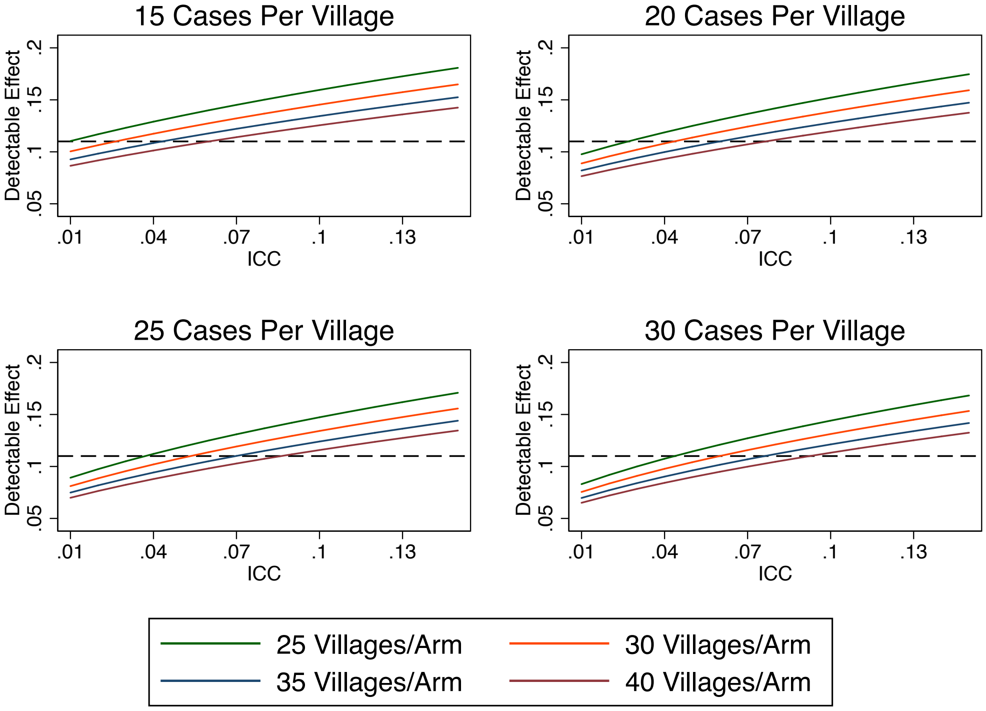

Figure 2: Detectable Effect Under Different Assumptions About Sample

6.1.2 Assignment to Treatment

Random assignment of villages will be stratified by BRAC branch (5 villages in each group per branch) and baseline ORS use. Baseline ORS use will be split into quintiles within each branch and random assignment will ensure that 1 village from each quintile-branch is in each of the 4 groups. We will use the *randtreat* package in Stata 14 to carry out this process.

6.1.3 Statistical Power

With 120 villages, 80 households enrolled in each village, and 25% of children having a case of diarrhea in the past month (UDHS, 2011), we expect to have a final sample of 2400 cases of diarrhea (600 per group). Assuming an intra-class correlation (ICC) of .05, we will be able to detect a minimum of an 11-percentage point increase in ORS use between each group, with a comparison group mean of 50% (UDHS 2011). However, we were unable to find a good estimate of the ICC, and therefore we are uncertain of the validity of this assumption. Moreover, there is also some uncertainty in regard to some of our other assumptions as well (e.g. diarrhea prevalence, number of households with a child under-5 per village, number of villages due to budgetary restrictions etc.). We therefore also conduct a series of additional sample size calculations under different assumptions (Figure 2). This figure shows that the detectable effect is particularly sensitive to the ICC. However, even under the worst case scenario—if we are short on funding and can only enroll 25 villages per arm, if the ICC is 0.15, and there are only 15 cases of diarrhea per village—we will still be able to detect a difference of 18 percentage points. During piloting of the Group 2 intervention we found that ORS use increased from 56% at baseline to 94% after the intervention. Therefore we expect that we will be sufficiently powered to detect important effects of these interventions.

6.2 Field Work

6.2.1 Instruments

Our survey instrument is attached as Appendix 1. Survey instruments were programmed into tablet devices which will be used for electronic data collection. We will use 3 survey instruments.

1) **Baseline Survey:** This instrument will be used to collect information on outcomes prior to the interventions, including whether a diarrhea episode occurred in the prior 4 weeks and which diarrhea treatments were used. The baseline survey will also collect information on ORS availability including price paid for diarrhea treatments (if used recently) and distance to nearest ORS distributor.
2) **Follow-up survey:** In addition to the outcome and access information collected at baseline, the follow-up survey will collect a variety of demographic information and other characteristics of the children, households, and caretakers enrolled in the study. This survey will also collect information on knowledge of diarrhea and proper treatment practices.
3) **CHP survey:** All 120 CHPs enrolled in the study will complete the CHP survey which will collect information on village characteristics and CHP practices.

6.2.2 Data Collection

Phase 1 — Listing of households (2 days per village): In the first phase of data collection, CHPs will create a list of all the households with a child under 5-years-old in their catchment area that includes the name and nickname of the household head. Our enumerators will use this list as a sampling frame to track households to enroll. Household will be listed prior to random assignment to avoid cherry picking by CHPs.

Phase 2 — Baseline Survey (1 day per village): Six teams of six enumerators will use the list provided by the CHPs and travel to the 40 closest households on the list (with the CHPs guidance) to conduct a baseline survey. Questionnaires will be completed by primary caretakers and will be recorded in tablet devices. Caretakers that reported a child to have had diarrhea in the past 4-weeks will be asked detailed questions about the diarrhea episode and caretaker treatment decisions. Enumerators will move on quickly from households with no recent diarrhea episode only recording that they did not have an episode (e.g. no demographic information). The CHP survey will also be conducted during the baseline phased.

Phase 3 — Endline Survey (1.5 days per village): Four weeks after the intervention is implemented, all households will be re-visited and asked to complete a follow-up survey that will follow the same protocol as the baseline survey, although it will capture more detailed information no demographics, village level characteristics, and knowledge of diarrhea treatment. Four weeks will be sufficient time for roughly 25% of children to have a post-intervention diarrhea episode (UDHS, 2011).

6.2.3 Data Processing

Data will be collected on tablet devices and stored on a BRAC server. Each survey will be automatically sent to the server via Internet. Raw data will be kept in tact and additional cleaned data sets will be created in Stata. Wagner, Asiimwe, Levine, and Dow will have full ownership over the data.

7 Empirical Analysis

7.1 Variables

7.1.1 Primary Treatment Outcome: ORS Use

The primary outcome for the study is self reported ORS use for a case of child diarrhea that occurred within the last 4 weeks. This will be measured through a series of survey questions which ask caretakers who reported having a child who had a diarrhea episode in the past 4 weeks the following:

1. Did you give (CHILD NAME) anything to treat the Diarrhea?

2. If yes, can you tell me or show me what treatments you gave (CHILD NAME) (either home-prepared or from outside of home)

3. If yes, can you tell me if you gave [CHILD NAME] any of the following treatments [INTERVIEWER WILL READ LIST]

Responses for (2) will not be prompted by the interviewer. For (3), respondents will be read the following list and asked if they used each of the treatments.

1. ORS
2. Zinc
3. Home-prepared treatment
4. Antibiotics

Our main outcome variable will be a binary variable that is set to 1 if the respondent reports that they used ORS in (3) and to 0 if they reported that they did not use ORS in (3) or if they reported that they gave no treatment to the child in (1). We will conduct a robustness check where we use the unprompted response from (2) to create our ORS use variable.

7.1.2 Secondary Treatment Outcomes: Zinc+ORS and Antibiotic Use

We will conduct an identical process for creating secondary treatment outcomes; zinc+ORS and antibiotic use. Zinc+ORS will be set to 1 if the case was treated with both zinc and ORS. All treatment outcomes will be set to missing if 1) the child was not reported to have had diarrhea in the last 4 weeks, or 2) if the caretaker did not know whether the child was given the respective treatment.

7.1.3 Time to Treatment Initiation Outcomes

For ORS and zinc treatments, we are also interested in the time between the diarrhea episode initiation and the treatment initiation. It is recommended by the WHO that both ORS and zinc are started immediately after the first symptoms of diarrhea. We will measure this using the following question, which will be asked to all caretakers that report giving the respective treatment to the child.

"How many days after the diarrhea began did you first give (CHILD NAME) [ORS/zinc]?" The enumerator will report '0' if treatment began on the same day as the diarrhea episode.

We will this measure 2 ways. First, for our main analysis we will keep this variable with days the units and truncate to 7 days to avoid influence of potential outliers. Next, we will also create a binary variable set to 1 if the caretaker started treatment on the same day that the diarrhea began.

7.1.4 Controls/Balance Check Variables

Caretaker Characteristics: We will create variables for caretaker's age (Question 101), education (none, primary, secondary+) (Question 102), marital status (Question 103), number of children (Q104), and employment status (Q115), ever used ORS (Q121), ever used zinc (Q122), heard of ORS (Q120), heard of zinc (Q126), aware of free ORS (Q124), aware of free Zinc (Q124), awre of CHP in village (Q290), visited by CHP in past 4 weeks (Q294),

Child Characteristics: We will create variables for child gender (Q202), age (Q203), frequency of diarrhea (Q206), and concurrent fever (Q210), blood in stool (Q211), diarrhea in last 4-weeks (question 2 from form B)

Household Characteristics: We will create variables for type of latrine used (covered, uncovered, or bush) (Question 109), main source of drinking water (piped, protected well/borehole, open well, surface water (river, damn, lake, etc.)) (Question 110), main source of income (agriculture, private sector, public sector, informal sector) (Question 113)

Baseline Village Characteristics: We will collapse the following information to the village level using the baseline survey wave only.

1. *Baseline ORS/Zinc/Antibiotic use:* A potentially important control variable is baseline treatment use, since this will adjust for potential preexisting differences in use between villages that were not balanced between groups after randomization. Moreover, baseline treatment use at the village level is likely a strong predictor of endline treatment use, and including it as a covariate will likely increase the power of our estimates. Since we will have different children at baseline and endline, we will not be able to control for each child's treatment use at baseline, which is why we will control for *village level* treatment use. We will create this variable by taking the mean of each treatment variable (ORS, zinc, and antibiotics) for each village at baseline.

2. *Baseline CHP Visit in Last 4-Weeks:* We will create this variable by taking the mean of Q294 by village.

3. *Baseline Access to Free ORS:* We will create this variable by taking the mean of Q124 (caretaker aware of free ORS) by village.

4. *Baseline Home ORS Storage:* We will create this variable by taking the mean of Qb1, which asks all respondents if they currently have ORS stored in their home (not just those with a diarrhea episode).

All of the above variables relating to ORS will also be created for ORS+zinc.

7.2 Balance Checks

Since our primary research questions compare Group 2 to the control group, Group 2 to Group 3, and Group 2 to Group 4, we will present all balance checks in terms of these comparisons.

We will test for balance between groups on both exogenous characteristics that should be unaffected by our interventions as well as endogenous characteristics that are likely to be affected by our interventions. For the exogenous characteristics listed in the section above, we will test for balance using data from our endline survey among the sample used for our main analysis (i.e. had diarrhea episode in the past 4 weeks). This sample will be different from our baseline analysis sample, since a different set of children will have experienced a case of diarrhea. Table **??** portrays how balance on exogenous variables will be presented.

For variables that are endogenous to our interventions (diarrhea treatment patterns, knowledge of treatment, access to treatment, and contact with CHP), we will test for balance using our baseline sample. Although this tests for balance among a different sample than used in our main analysis, the sample of villages remain the same, and this will provide a sense of village level balance before the interventions. We will include estimates from the full sample for questions referring to obtaining ORS and zinc, home storage of ORS, and all other variables will only be assessed for households with a case diarrhea. Table 2 portrays how balance on exogenous variables will be presented.

We will also fit a multinomial logit model with group type (Group 1-Group 4) as the dependent variable and report the χ^2 test statistic as the joint test for equality for each of the exogenous and endogenous variables. This will be reported in the appendix, Table XX.

7.3 Evaluation of Intermediate Outcomes

First, we will test for whether the intervention appeared to be carried out properly by measuring differences in CHP behavior in the past 4 weeks: home visits, delivery of ORS and zinc, free distribution of ORS and zinc. Next we will assess how each intervention affected acquisition and home-storage of ORS and zinc (at time of survey, any time in last 4 weeks, and when diarrhea episode initiated). We will fill in the cells of Table 3.

7.4 Treatment Effects

Our main analyses will be conducted at the child level. This is equivalent to the diarrhea episode level since most children will only have had one case of diarrhea and we only inquire about one case per child.

7.4.1 Reduced form

We will conduct several analyses to assess the impact of each treatment arm on ORS use. We will refer to our main estimates as reduced form estimates since it is possible some households in our treatment groups will not receive the treatment (e.g. if the CHP fails to make the delivery or does not comply with instructions to provide the products for free). All regressions with binary outcomes will be estimated using a logit model, which should produce similar estimate to a linear probability model (LPM), but with greater efficiency. We will present average marginal

effects from logit models using the delta method. We will use linear regression models (ordinary least squares) to assess continuous outcomes.

First, we will run an unadjusted regression using the post data only. We present all analyses in terms of ORS use, but analogous analyses will be conducted for each outcome of interest. We will estimate equation 1.

$$Pr(ORS_{iv}) = expit(\beta_0 + \beta_1 Group2_{iv} + \beta_2 Group3_{iv} + \beta_3 Group4_{iv} + \epsilon_{iv}) \quad (1)$$

We can then use this equation to estimate the following:

$\mathbb{E}[ORS|Group = 2] - \mathbb{E}[ORS|Group = 1]$ (*Primary Question 1*): The impact of the combined effect of free distribution, preemptive home delivery and information.

$\mathbb{E}[ORS|Group = 2] - \mathbb{E}[ORS|Group = 3]$ (price-effect, *Primary Question 2*): The effect of preemptive free home-delivery relative to preemptive home sales.

$\mathbb{E}[ORS|Group = 2] - \mathbb{E}[ORS|Group = 4]$ (convenience effect, *Primary Question 3*): The effect of preemptive free home-delivery relative to free distribution upon retrieval from the CHP's home.

$\mathbb{E}[ORS|Group = 3] - \mathbb{E}[ORS|Group = 1]$ (*Secondary Question 2*): Impact of preemptive home sales and information (i.e. with no free-distribution)

$\mathbb{E}[ORS|Group = 4] - \mathbb{E}[ORS|Group = 1]$ (*Secondary Question 3*): The impact of free-distribution upon retrieval from the CHPs home and information (no preemptive home access).

$\mathbb{E}[ORS|Group = 3] - \mathbb{E}[ORS|Group = 4]$ (price effect vs. distance/convenience effect, *Tertiary Question 1*): The effect of preemptive home sales relative to free-distribution upon retrieval from the CHP's household.

The estimates above do not account for potential baseline differences in ORS use between groups, nor do they account for potential confounders that might not be balanced between groups at baseline. To account for potential imbalance in baseline ORS use, we will include average village level ORS use at baseline as a covariate. When autocorrelation in the outcome is low, this is a more efficient way of controlling for baseline outcomes than a difference-in-differences approach (McKenzie, 2012).[2] We will also control for a set of caretaker, child, and village level characteristics to account for potential differences between treatment and control groups that could confound our estimates and to improve precision (see section 7.1 for details on how variables were created). We will run one regression that includes all of the following:

Caretaker Characteristics: age, education, number of children
Child characteristics: age, diarrhea frequency per month, blood in stool, concurrent fever

[2]Ideally, we would have each child's baseline ORS use, however the set of children with diarrhea episodes at endline is different than the set of children with diarrhea episodes at baseline, which makes autocorrelation likely to be low

Household Characteristics: water source, latrine type, main source of income
Baseline Village Characteristics: % of households visited by CHP in past month, % of households aware of free ORS in Village, travel time to nearest ORS distributor, % of households with ORS stored in their home

We will also include indicators for each BRAC branch corresponding to each village (6 in total).

$$Pr(ORS_{ivt}) = expit(\beta_0+\beta_1 Group2_{iv}+\beta_2 Group3_{iv}+\beta_3 Group4_{iv}+\beta_4 ORS_{v(t-1)}+X_{iv}\beta_5+\lambda_b+\epsilon_{ivt}) \quad (2)$$

Here we include the average ORS use in the child's village at baseline on the right hand side of the equation ($ORS_{v(t-1)}$). X_{iv} is a vector of caretaker, child, and village characteristics, and λ_b is a set of branch fixed effects.

Equation 2 is our preferred model since it should improve precision and control for potential bias that results from imbalance in important covariates. Example tables for the analyses from this section are presented in tables 3 - 5.

The above equation will account for any potential bias that occurs as a result imbalance in confounding factors between groups. However, the model fit that results from this crude inclusion of covariates can be improved upon by using a data adaptive method for including covariates. To better fit our model and improve the precision of our estimates, we will use the Super Learner package in R to choose which control variables from those listed above that result in the best cross validated model fit. All potential covariate combinations will include baseline village level ORS use and branch indicators. We expect that this model will produce more precise estimates of the treatment effects but to do a worse job of reducing bias that results from imbalance at baseline.

7.4.2 Time to ORS Use

We will estimate the impact on time to ORS initiation after diarrhea initiation (*Secondary Questions 10-12*) using two different units. First we will use days as units. For this we will use a cox proportional hazard model (truncated at 7 days) and include the same covariates as equation 2 for these estimates. Next will use a binary outcome that indicates that treatment was started on the same day as diarrhea (the recommendation by the WHO). For this we estimate the logit model from equation 2. We will present these results according to table 6.

7.4.3 Impact Of Home Storage: Instrumental Variables Analysis

Our reduced form estimates will help answer the question of whether the program and variation in how the program is designed effects diarrhea treatment practices. However, as mentioned above, some of the CHPs might not comply with intervention guidelines and therefore some households might not receive the program. A more fundamental question is whether having

ORS and zinc stored in the household preemptively (i.e. prior to a diarrhea episode) results in higher use than having to go retrieve the product once a diarrhea episode begins. If this is the case, then other interventions beyond CHP delivery could be used to increase home storage rates. In order to answer this question, we will use an instrumental variables approach where we use random group assignment as an instrument for preemptive home storage. For this analysis we will only use participants in Groups 2 and 4 since both groups have free distribution combined with information, with the only difference between groups being that Group 2 will have the products delivered prior to a diarrhea episode. We expect this to increase the probability of having the ORS and zinc stored in the household preemptively when the child comes down with diarrhea. We will use two-stage least squares (2SLS) to estimate the following equations.

First Stage:

$$Store_{ivt} = \beta_0 + \beta_1 Group2_{iv} + \beta_2 ORS_{v(t-1)} + X_{iv}\beta_4 + \lambda_b + u_{ivt} \tag{3}$$

Second Stage:

$$ORS_{ivt} = \alpha_0 + \alpha_1 Store_{iv} + \alpha_2 ORS_{v(t-1)} + X_{iv}\alpha_4 + \lambda_b + \epsilon_{ivt} \tag{4}$$

The key assumption that has to hold for random assignment to be a valid instrument is that random assignment into Group 2 instead of to Group 4 only affects ORS use through increasing home storage (i.e. $Group2 \perp \epsilon$). Since the only difference between groups is the timing and location at which ORS was provided for free, we expect this assumption to hold. This exclusion restriction might not hold if Groups 1 and 4 were included in the analysis.

We expect β_1 to be positive and significant, implying that assignment to $Group2$ indeed increases the probability of home-storage. We will reject assignment to Group 2 as a "weak instrument" if the *F-Statistic* from equation 3 is equal to 10 or greater. We also expect α_1 to be positive, implying that preemptive home-storage of ORS increases ORS use.

Table 7 presents an example table from equations 3 and 4.

7.5 Heterogeneous Treatment Effects

For targeting purposes it might be helpful to understand for what types of villages or households these interventions will be most effective. We will assess how the program affects outcomes differently based on several characteristics. All analyses in this section are exploratory and are likely underpowered.

7.5.1 Heterogeneity by ORS Access

We expect this program will be particularly effective for areas that are farther away from ORS and zinc distributors. Therefore, we will measure how each treatment arm affects outcomes differently for villages that are farther away from distributors (*Tertiary Question 12*). We will measure distance, as described in section 7.1, in terms of self reported *time* to reach the nearest place that distributes ORS.

In order to assess heterogeneous treatment effects by distance, we will create interaction terms that interact distance (time it takes to arrive at the nearest ORS distributor) with each treatment group indicator. We will then estimate the following equation.

$$ORS_{ivt} = \beta_0 + \beta_1 Group2_{iv} + \beta_2 Group3_{iv} + \beta_3 Group4_{iv} + \beta_4 Dist_v +$$
$$\beta_5 DistXGroup2_{iv} + \beta_6 DistXGroup3_{iv} + \beta_7 DistXGroup4_{iv} +$$
$$\beta_8 ORS_{v(t-1)} + X_{iv}\beta_9 + \lambda_v + \epsilon_{ivt} \tag{5}$$

Equation 5 tests how treatment effects vary by time to reach the nearest ORS distributor. A positive and significant coefficient on the interaction terms will suggest that people that had less access to ORS distributors (further away) experienced a larger improvement from the interventions, suggesting that lack of access/availability was an important barrier that the interventions helped overcome. Since all groups increase access to ORS, we do not expect heterogeneous effects to be different across groups. See table 9 for how these results will be presented.

7.5.2 Heterogeneity by Child Vulnerability

It is also important to understand how each of our interventions affects ORS use among the most vulnerable children (*Tertiary Question 11.* For example, does free delivery expand coverage to children that were not likely to die from ORS? Does charging for ORS or requiring small hassle costs do a better job of targeting resources to the most vulnerable than giving ORS away for free? To assess these questions, we will assess heterogeneity in intervention impacts by two different measures of child vulnerability.

1. **Age:** The majority of diarrheal mortalities happen within the first year of life. We will use a dummy variable indicating that the child is less than 12 months old.
2. **Severity of Episode:** There are two criteria used to identify severe episodes: concurrent fever and blood in the stool. We will code a case as "severe" if either of these criteria are satisfied.

We will test for how ORS use is affected differently based on these characteristics using the same interaction model framework outlined in equation 5.

$$ORS_{ivt} = \beta_0 + \beta_1 Group2_{iv} + \beta_2 Group3_{iv} + \beta_3 Group4_{iv} + \beta_4 Age_v +$$
$$\beta_5 AgeXGroup2_{iv} + \beta_6 AgeXGroup3_{iv} + \beta_7 AgeXGroup4_{iv} +$$
$$\beta_8 ORS_{v(t-1)} + X_{iv}\beta_9 + \lambda_b + \epsilon_{ivt} \tag{6}$$

$$ORS_{ivt} = \beta_0 + \beta_1 Group2_{iv} + \beta_2 Group3_{iv} + \beta_3 Group4_{iv} + \beta_4 Severe_v +$$
$$\beta_5 SevereXGroup2_{iv} + \beta_6 SevereXGroup3_{iv} + \beta_7 SevereXGroup4_{iv} +$$
$$\beta_8 ORS_{v(t-1)} + X_{iv}\beta_9 + \lambda_b + \epsilon_{ivt} \tag{7}$$

See table 9 for how these results will be presented.

7.5.3 Super Learner for Heterogeneous Treatment Effects

In addition to the heterogeneous treatment effects described above, we will use a data adaptive approach to test for heterogeneity in the intervention effects by a series of other characteristics that could modify the treatment effect. To do this, we will use the Super Learner package in R.

7.6 Targeting subsidized ORS to those that will use it

Ideally, a policy maker would like to use subsidies to maximize coverage of health products while minimizing wastage of these subsidized products. This is achieved by giving products to people with a high propensity for use (compliance). Free distribution vs. cost sharing for health products has been a contentious issue in terms of targeting health products to those most likely to use them. Proponents of charging for health goods argue that people don't value products that are given away for free (PSI, 2003). Charging for products could increase use through the *sunk cost effect* (Thaler, 1980) and improve targeting and reduce wastage through the *selection* or *screening* effect (Ashraf et al., 2010; Cohen and Dupas, 2010). However, public health proponents often argue that charging for health products will reduce coverage by dampening demand particularly among the poor and vulnerable. Cohen and Dupas (2010) find no evidence of the sunk cost effect or the screening effect when comparing free distribution and cost-sharing for bed nets in Kenya—pregnant women who got free nets were no less likely to use them than women who paid for the nets. However, even highly subsidized prices dramatically reduced coverage of bednets relative to free distribution. This suggests that free distribution increased coverage without affecting wastage. Ashraf et al. (2010) also find no evidence of the sunk cost effect for point of use water treatment in Zambia, however they did find evidence of the screening effect—households that had a higher propensity to use the product were willing to pay a higher price. This suggests that increasing prices could indeed improve targeting but at the expense of reducing coverage. Dupas et al. (2016) revisit prices, take-up, and wastage of point-of-use water treatment in Kenya by comparing subsidized prices, free distribution upon retrieval (hassle cost), and free delivery. They found that the hassle cost of retrieving the free product vs. having it freely delivered reduced take-up by 60% but had no effect on product use. On the other hand, a 50% price discount reduced take-up by 50% but also reduced use by 62% relative to free delivery. This suggests that imposing non-monetary hassle prices could be a more efficient way of reducing wastage than charging for products.

Our experimental design is very similar to Dupas et al. (2016) in that we are comparing free delivery, free upon retrieval (hassle costs), and cost-sharing. Free delivery of ORS will result in take-up of close to 100%. However, the product could be delivered to caretakers that have a low propensity to use ORS resulting in wastage. Requiring that caretakers pay for ORS could reduce wastage through a screening effect but is also likely to exclude some caretakers who would use the product if it were provided for free. However, requiring the hassle of retrieving free ORS from the CHP's home could weed out the caretakers with low propensity to use the product without excluding those that would use the product if available for free, improving targeting without compromising coverage.

We will assess how well the subsidized ORS from our interventions are targeted to those with a high propensity for ORS use by comparing the share of participants that obtained subsidized ORS from the CHP and the share of participants that used ORS to treat a case of diarrhea. Let α_i be the share of respondents with a diarrhea episode in intervention i that acquired subsidized ORS from the CHP during our study period and μ_i be the share of respondents that used ORS

to treat a case of diarrhea during our study period. Let $\lambda_i = \frac{\mu_i}{\alpha_i}$, be the measure of how well the product was targeted to those that will use it (hence forth referred to as compliance). There are three important inequalities of the $\lambda's$ and $\mu's$:

1. $\lambda_i > \lambda_j$ and $\mu_i < \mu_j$: this implies that intervention i does a better job of targeting subsidies to people that will use the product appropriately than intervention j, but intervention j does a better job of getting ORS to all that need it.
2. $\lambda_i > \lambda_j$ and $\mu_i >= \mu_j$: this implies that intervention i does a better job of targeting subsidies to people that will use the product appropriately than intervention j, and also does at least as good of a job at getting ORS to all that need it.
3. $\lambda_i >= \lambda_j$ and $\mu_i > \mu_j$: this implies that intervention i does at least as good as good of a job of targeting subsidies to people that will use the product appropriately, but does a better job at getting ORS to all that need it.

Estimating λ and μ for each of our study arms will allow us to assess the trade-off of targeting ORS subsidies to those that will comply and increasing ORS coverage. However, we are underpowered to assess equality (i.e. precise zeros) of $\lambda's$ and $\mu's$ between the two study arms, which is a criteria for scenarios 2 and 3 above and therefore this analysis should be thought of as exploratory. For example, if we find no significant difference between μ_i and μ_j, the confidence interval of the difference will likely include both important positive and negative values, making it difficult to assess which of the above scenarios is satisfied. Moreover, since ORS is extremely cost-effective, even very small differences in coverage (μ) are important.

Regardless of the limitations of this analysis, we expect this exercise to contribute to the limited evidence on targeting subsidies with price and non-price mechanisms. Based on prior evidence, we would expect to find that preemptively selling ORS at a subsidized price will produce a larger λ (those that purchase ORS will be more likely to use it) relative to free delivery, but also a smaller β (fewer cases will be treated with ORS) (Dupas et al., 2016; Ashraf et al., 2010). Moreover, we would expect to find that the non-price hassle cost of free distribution upon retrieval from the CHP's home will produce a larger λ without compromising coverage (scenario 2 above), since such costs are not impeded by financial constraints (Dupas et al., 2016).

We will measure μ as specified in section 7.1.1, which is our primary outcome. To measure α and λ, we will use a survey question asking respondents if they received any ORS from the CHP in the last 4 weeks. For our statistical analysis of targeting, we will estimate differences in α and μ using equation 2. See table 10 for how our targeting analysis will be displayed.

In some cases a caretaker might use the subsidized ORS to treat a child older than 5-years-old and thus not have ORS available to treat her younger child. Our μ estimate will treat this scenario as "not using ORS to treat a case of diarrhea" thus reducing μ and λ, although this does not necessary indicate non-compliance. We will test for how sensitive our results are to this scenario by coding these both as .5 treated case and 1 treated case (in separate analysis).

In addition to our measures of efficient targeting above we will assess two additional measures that could indicate poor targeting. Among households that receive ORS/zinc, we will assess:

1. **Lost Packets:** They no longer have the product stored in their home and they did not use the product to treat a case of diarrhea (recorded for all households including those without diarrhea)

2. **Inappropriate Use:** A non-child family member used the product

This analysis is will be presented in table 11.

7.7 Cost-Effectiveness

Although the section above assesses the trade-off between targeting and coverage, it does not fully compare which intervention allocates resources in the most efficient way. In order to further assess this, we will compare the incremental cost of each intervention in terms of US dollars and the incremental benefit of each intervention in terms of DALYs averted. We will estimate the cost of each intervention as the cost the intervention itself (ORS packets), the cost of the CHPs time for the household visits, and the travel costs and time costs associated with a case of diarrhea. The cost of ORS packets will be estimated based on the share of households that acquired ORS packets (α) and the cost per packet incurred by BRAC (about \$0.07 for the free interventions and about \$0.03 for the sales interventions). The cost of the CHPs time will be estimated based on the time it takes to make household visits (about 3 days) and the daily wage for the CHP (based on BRAC administrative records). The time and transport costs for the caretaker will be estimated from our survey, which directly asks how long it took to arrive at an ORS distributor and the cost of travel. We will use local agriculture wages to estimate time cost. We will not account for time spent caring for the sick child since this is expected to be the same across all groups. All costs will be estimated as cost per household in the sample.

We will estimate the DALYs averted that correspond to incremental coverage with ORS. We will feed ORS coverage rates (μ) into the Lives Saved Tool (LiST) to estimate the incremental lives saved per case of diarrhea treated with ORS customized to parameters from Uganda. We will not include disability in our DALYs estimates since disability effects from ORS are negligible.

We will include all costs and DALYs averted that occur over the 4-week intervention period. This includes costs for households that did not have a diarrhea episode but excludes benefits for these households that are likely to occur in the future. Most households will eventually have a case of diarrhea (75% of households reported an episode at least every 4 months), which implies that these estimates will be a lower bound. This will also likely penalize the free delivery group more than the other groups since take-up will likely be near 100% even among households with no diarrhea case. As a result, we will also conduct our cost-effectiveness analysis restricting to only the sample that had a case of diarrhea during the study period.

When comparing intervention i to intervention j, we will estimate the following incremental cost-effectiveness ratio (ICER).

$$ICER_i = \frac{Cost_i - Cost_j}{DA_i - DA_j} \tag{8}$$

DA=DALYs averted.

We will conduct a 1 one way sensitivity analyses where we vary each parameters individually to test for the individual impact of each parameter on our ICERs. We will conduct a 2-way sensitivity analysis, where we re-estimate equation 8 using the full range of the differences in α's and μ's produced by the 95% confidence intervals estimated in table 10, varying the parameters simultaneously. Finally, we will also conduct a multi-way sensitivity analysis where we vary all parameters at once.

Table 12 presents example tables for the cost-effectiveness analyses described in this section.

7.8 Standard Error Adjustment

All standard errors will be clustered at the village-caretaker level, the level at which the randomization/intervention will occur. Multiple hypothesis testing adjustments will not be used for the primary outcome, ORS use, since there is only 1 primary outcome. We will us the free step-down resampling method to control the False Discovery Rate (FDR) to adjust standard errors for secondary and tertiary outcomes since we have multiple secondary/tertiary outcomes (Anderson, 2012). We will adjust for multiple hypotheses within the following categories.

1. Intermediate Outcomes: We will adjust for the 19 intermediate outcomes included in table 3
2. Secondary Treatment Outcome: We will adjust for the 3 secondary treatment outcomes (zinc+ORS use, antibiotic use, time to ORS use) in tables 5 and 6
3. Heterogeneous Treatment Effects: We will adjust for the 3 outcomes assessed for heterogeneity in table 9

7.9 Outcomes

We assess the impact of the interventions on the following outcomes:

- **Primary Outcome** (Table 4):
  - Used ORS in last 4 weeks
- **Secondary Outcomes** (Table 5 and Table 6):
  - Time to ORS use
  - Used ORS+Zinc
  - Used antibiotic
- **Tertiary Outcomes** (Table 3):
  - Obtained ORS in last 4-weeks
  - Obtained free ORS in last 4-weeks
  - ORS stored: currently
  - ORS stored: last 4 weeks
  - ORS stored: diarrhea initiation
  - Obtained zinc in last 4-weeks
  - Obtained free zinc in last 4-weeks
  - Zinc stored: currently
  - Zinc stored: last 4 weeks
  - Zinc stored: diarrhea initiation

- Home visit from CHP in last 4-Weeks
- Visited CHPs home in last 4-Weeks
- CHP provided any ORS in last 4-Weeks
- CHP provided free ORS in last 4-Weeks
- CHP provided ORS at home in last 4-Weeks
- CHP provided any zinc in last 4-Weeks
- CHP provided free zinc in last 4-Weeks
- CHP provided zinc at home in last 4-Weeks
- Caretaker visited health provider for treatment
- Heard of ORS
- Heard of zinc

8 Robustness Checks

8.1 Addressing Problems With Self-Reported Outcomes

Our main outcome measures are self-reported, which creates several problems. First, there is potential for social desirability bias where caretakers intentionally over-report ORS use. Second, there is potential for recall bias where caretakers mis-remember their past treatment behavior. If either type of measurement error in outcomes is correlated with treatment assignment, this would compromise the study's internal validity. We have several strategies for verifying and validating the self-reported outcomes, which are outlined below.

8.1.1 Intentional Over Reporting of ORS Use

It is possible that there is differential intentional over-reporting of ORS use in the treatment and control groups. For example, Group 2 and Group 4 respondents might over-report use since it was provided to them for free and they thought they would receive more free ORS in future if they reported using it. All treatment groups might over report ORS use relative to the control group since the CHP told them they were supposed to use it. We will account for this potentially differential over-reporting in several ways.

Counting Packets
In Group 2, we will provide incentives for caretakers to keep the ORS and zinc packets we provide (used and/or unused). Caretakers will be given $0.25 (USD) during the endline survey if they have any of the packets that were provided to them by the CHP as a result of the intervention. All packets delivered during the intervention will have a black mark with permanent marker for identification. Enumerators will record 1) if any packets was observed, 2) the number of used packets, and 3) the number of unused packets. We will then cross check the number of used packets with self-reported ORS use. We will code ORS use to 1 if the household was observed to have an empty packet and to zero otherwise. We will conduct a similar process for zinc use. We will then re-run equation 2 using this new ORS use variable and including only Group 2 and the Control Group (See Table 13). This will eliminate any

upward bias of our estimates that result from differential self-report bias in Group 2. However, it is important to note that this will account for any over-reporting of ORS/zinc use in Group 2, but will not account for over-reporting in the control group since we cannot verify packet use in the control group. Therefore, these estimates will represent a lower bound of the true effect of the group 2 intervention. Moreover, it would compromise our experimental design to do this procedure in the other treatment groups.

Placebo Tests
We will conduct a series of placebo tests to test for differences between treatment and control groups on self reported child health behaviors that should not be affected by the interventions. No effect on these outcomes will provide confidence that any effects on diarrhea treatment outcomes are a result of the intervention and not differential over-reported on healthy behaviors. We will use the following outcomes as placebo dependent variables in equation 3 with controls.

1. Gave child malaria treatment (conditional on symptoms)
2. Gave child food or liquid that was unclean
3. Child always slept under a bed net
4. Child washed hands at least twice per day

See table 14 for the table shell for this analysis.

8.1.2 Unintentional Misreporting Of ORS Use

Change in Recall Duration
We will ask respondents to recall diarrhea episodes and treatment behavior that occurred in the past 4 weeks. We use this recall period so that it is aligned with the period when the intervention was active and to satisfy our sample size criteria. However, it is possible that this duration is too long to produce valid estimates. Moreover, this measurement error from recall could be correlated with treatment assignment (e.g. free home delivery households might be better at remembering accurately). Such measurement error can cause unpredictable bias (Arnold et al., 2013). To account for this, we will restrict our analysis to 1) diarrhea cases that are ongoing during data collection (about one third of reported diarrhea cases), and 2) diarrhea cases that ended within 7 days of data collection which is the optimal time frame outline by Arnold et al. (2013). We will present this analysis in Table 15.

8.2 Attrition and Changes in Group Composition

8.2.1 CHP Attrition

In order for CHP's to carry out their randomly assigned intervention they must receive a training session that instructs them on the required tasks and provides them with enough ORS and zinc for all children under-5 in their village. In some cases a CHP/village could be randomly assigned to a treatment but not actually receive training on how to carry out the intervention or receive the ORS and zinc to distribute. This CHP attrition could occur in three main forms:

1. CHP at baseline is no longer a CHP at endline and there is no longer a CHP in the village

2. CHP at baseline has been replaced by a new CHP at endline in the same village and new CHP was trained on intervention protocol
3. CHP is unable to show up for training although she is assigned to treatment group

There is no reason to expect that (1) an (2) will occur differentially between groups. If (1) occurs in either the treatment or control groups, we will exclude households form this village from the analysis and test for sensitivity to including households from villages with no CHP at endline as a robustness check. If (2) occurs in either the treatment or control groups, we will include households from these villages in the main analysis, since the intervention was still carried out in treatment villages, and would have been carried out in the control villages had these villages been randomly assigned to treatment.

If a CHP is not trained due to inability to show up for the training session, this type of attrition could be differential between groups. It is possible that poor performing CHPs are more likely to not show up for training, and if households from villages with poor performing CHPs are excluded from the treatment villages but not the control villages, this could compromise the study's internal validity, since groups would no longer be comparable. If this occurs, we will address this using an intention-to-treat analysis as our *main analysis* where our indicator variables for treatment assignment will represent whether a CHP was assigned to a treatment, not whether she was actually trained on the intervention protocol. This will preserve the exchangability between groups generated by the randomization, however, it will provide a lower bound estimate the impact of the interventions, since whole villages assigned to treatment will not have received treatment. To get the treatment-on-the-treated effect, we will then use random group assignment as an instrument for whether the CHP was actually trained on the intervention protocol.

8.2.2 Differential Household Refusal

For some of the households in our sample, will have two observations (baseline and follow-up), but for many households, we will only have one observation (follow-up only or baseline only). One concern is that more households in villages where something was given away for free (groups 2 and 4) will agree to be surveyed at endline than in the groups where no gifts were given out (groups 1 and 3). If these additional households in the free distribution groups are systematically more or less likely to use ORS, this could compromise the study's internal validity, since groups would no longer being comparable. Since refusal to participate is very rare we don't expect differential refusal to be a huge issue. We will test for this in 2 ways. First, we will compare the refusal rate between two groups. Second, we will use Table 2 to assess whether groups are comparable at endline.

8.2.3 Differential Reporting of Diarrhea Episodes

Another more concerning channel through which group comparability could be compromised is through differential reporting of diarrhea episodes. Since the main outcome of interest (ORS use) is contingent on a child having had a recent case of diarrhea, we will only collect outcome information for children that had a recent diarrhea episode. Therefore, the children for whom we collect outcome information at baseline will mostly be different than the children for whom we collect outcome information at endline (since most children will not have two episodes during our study period). Equation 9 portrays the impact of intervention I relative to the control group c.

$$\frac{ORS_I + u_I}{D_I + \epsilon_I} - \frac{ORS_c + u_c}{D_c + \epsilon_c} \tag{9}$$

Where ORS represents the number of caretakers that reported using ORS to treat a case of diarrhea, D represents the number of caretakers that reported a diarrhea case, ϵ represents the number of caretakers that had a diarrhea episode but did not report it, and u represents the number of caretakers that used ORS to treat a case of diarrhea that was not reported. We will assume that:

$$\frac{ORS_j}{D_j} >= \frac{u_j}{\epsilon_j}, \forall j \tag{10}$$

This implies that the share of cases treated with ORS among unreported cases is not larger than that of reported cases. If for some I, $\epsilon_I \neq \epsilon_c$ this could be indicative of differential reporting of diarrhea episodes between caretakers in intervention I and the control group suggesting groups might not be exchangeable. We will test for this by estimating the following equation:

$$D_{ivt} = \beta_0 + \beta_1 Group2_{iv} + \beta_2 Group3_{iv} + \beta_3 Group4_{iv} + \beta_4 D_{v(t-1)} + \epsilon_{ivt} \tag{11}$$

Where D_{ivt} represents whether child i in village v at time t had a diarrhea episode reported. The coefficients β_1 - β_3 will indicate whether the treatment groups had a higher or lower probability of reported diarrhea relative to the control group, holding baseline village level diarrhea probability constant.

There are several potential scenarios that could arise that would be indicative of differential reporting.

Scenario 1 $\epsilon_i < \epsilon_c$: it is possible that caretakers in the treatment groups will be more likely to report a diarrhea episode at endline than caretakers in the control group for two reason: 1) treatment group caretakers could have the expectation that they will be provided additional free products if a child had an episode and thus overreport episodes (e.g $\epsilon_i < 0$) and 2) treatment group caretakers might be more likely to accurately recall a recent case of diarrhea as a result of the intervention. This scenario arises if any of β_1-β_3 are positive and statistically significant. Our assumption in equation 10 implies that this will bias our estimates towards the null. If this scenario arises we will provide a potential upper bound of our estimates by doing the following.

1. Assume that the control villages had the same diarrhea prevalence as treatment village by randomly assigning observations to have had a diarrhea episode among the sample in the control group that did not report an episode.

2. Assume $u_c = 0$, everyone in the control group that gets randomly assigned an episode did not use ORS (upper bound) .

Scenario 1 $\epsilon_i > \epsilon_c$: It is also possible that caretakers in the treatment groups under-report diarrhea episodes relative to the control group. For example, this could occur if caretakers who failed to use ORS that was provided for free were embarrassed to admit this, as it could be perceived as negligence, so they instead say there was no episode. This will result in an upward bias. If any of β_1-β_3 are negative will do the following.

1. Assume that the treatment village had the same diarrhea prevalence as the control village by randomly assigning observations to have had a diarrhea episode among the sample in the respective treatment group that did not report an episode.
2. Assume $u_I = 0$ everyone in the treatment group that gets randomly assigned an episode did not use ORS (lower bound).

We will only conduct this analysis if we find differences in diarrhea prevalence and therefore do not provide a table shell at this stage.

8.3 Ruling out alternative mechanisms

Our intervention arms are designed to isoloate for the role of price and convenience in ORS use. However, there potentially other mechanisms that differ between groups aside from price and convenience.

First, it is possible that providing ORS for free is a signal to caretakers that ORS is particularly important, which could increase product salience. If this is true, then comparing *free home delivery* to *home sales* does not isolate for the impact of free-distribution, but rather is the combined effect of free-distribution and increased salience. To test for whether groups differ in terms of ORS salience, we will use a question asking "If your child becomes sick with diarrhea, what do you think is the best way to treat the child?" (Q301). We will compare the share of caretakers in each group that reported ORS as the best way to treat their child's diarrhea.

Next, it is possible that CHPs in the preemptive home sales group will exert more effort in visiting households and distributing ORS, since they will receive payment for each packet sold. If this is true, then comparing *free home delivery* to *home sales* does not isolate for the impact of free-distribution, but rather compares *free distribution* to *cost-sharing plus increase effort.* To test for differential CHP effort, we will compare groups on two measures effort: number of visits in last four weeks and duration of longest visit in the last 4 weeks.

9 Discussion

This work will contribute to several bodies of existing literature. First, this work will evaluate the impact of a novel way of utilizing community health workers to increase ORS and zinc use (free and preemptive delivery). Community health worker programs are increasingly relied on to increase access to health care and improve health outcomes in rural communities throughout the developing world. This work will help understand how best to use community health workers to increase ORS and zinc use (free distribution, home delivery, or both).

Second, this work will be the first to provide experimental evidence on the role of price and convenience in ORS and zinc use. Although there are a lot of resources invested in programs aimed at increasing ORS and zinc use, evidence on the remaining barriers to ORS use and what works to increase use is scarce.

Third, this study will be the first to assess the causal impact of having ORS stored at home prior to the initiation of a diarrhea episode on ORS use. If home storage is found to positively influence ORS use, this would suggest that other interventions beyond CHP deliveries could be used to increase home storage (e.g. preemptive free distribution at clinics or distribution in larger quantities). Moreover, the share of caretakers that do not use ORS even if they had it stored at home allows for measurement of the extent to which other factors un-related to

price and convenience—cultural beliefs, preference for other medicines, poor taste—affect ORS use.

Fourth, our findings will contribute to the ongoing debate about free distribution vs. cost-sharing for health products. If caretakers appear to be sensitive to ORS price, consistent with the literature on preventive health products (Kremer et al., 2011a), then a program of free distribution could be more effective than the status quo of subsidized prices charged by CHPs.

Fifth, the comparison of hassle cost, monetary prices, and free delivery will contribute to the evolving literature on efficient targeting of subsidies. If our findings are consistent with Dupas et al. (2016) and we find that hassle costs (free retrieval) provide better targeting than free delivery without compromising coverage, this could imply that imposing small hassle costs is the preferred way to distribute ORS.

This work is limited in several ways. First, our main outcome measure relies on self reports. Although we have several strategies in place to validate our self-report measures, they could still introduce measurement error and potentially bias our results. Second, we only assess the short term affects of these interventions. It is unclear if the impacts will be sustained over time when the novelty and salience of the interventions wear off. Moreover it is unclear how well CHPs will perform at continually delivering ORS and zinc to households each month, which could diminish the intervention's effectiveness over time. Future work should aim to assess the long term impacts of these interventions as well as different ways of motivating community health workers to continually carry out the interventions. Third, we do not observe the same child at baseline and endline since many children will not have multiple cases of diarrhea. This makes it impossible to compare balance on and adjust for differences in baseline outcomes for the analysis sample of our four groups and requires us to instead use village-level baseline outcomes. However, in expectation randomization will result in balance on all baseline characteristics. Fourth, in our analysis of efficient targeting of subsidies (section 7.6), we do not observe what will happen for households with no diarrhea episode. It is possible that acquisition of ORS prior to initiation of a diarrhea episode is less efficient for episodes that happen farther in the future since this provides more opportunity to lose, use for undesired purpose, or give away the ORS. Moreover, some households that acquire ORS preemptively might never have a child with a diarrhea episode.

10 Conclusion

ORS and zinc are extremely effective at preventing mortality from diarrhea, yet they remain largely under-used. As a result children continue to die. This research will provide insight into the mechanisms that are likely help increase ORS use. The results could be used to improve CHP programs in Uganda and around the globe, and have the potential to reduce child mortality.

11 Research Team

This project is led by Zachary Wagner and John Bosco Asiimwe under the supervision of William H. Dow and David I. Levine. The research team works closely with Munshi Sulaiman and Robert Mpiira at BRAC and BRAC's community health promoters.

12 Deliverables

We will produce the following deliverables from this project.

- Job market paper for Zachary Wagner that includes everything outlined above (Authors ZW and JBA)
- Article aimed at medical journal audience that assesses the impact Group 2 on ORS and zinc use (Authors ZW JBA DIL WHD)
- Article aimed a health economics journal audience that assess mechanisms through which the intervention worked and highlights the role of price and convenience of ORS and incorporates the targeting analysis (Groups 3 and 4 compared to Groups 2) (Authors ZW JBA DIL WHD)
- Cost-effectiveness analysis (Authors ZW JBA DIL WHD)
- Report on the findings for BRAC

13 Calender

- June-July 2016: Baseline Surveys
- January 2017: Role out of interventions
- February-March 2017: Endline Surveys
- April 2017: Job Market Paper

References

Michael L Anderson. Multiple inference and gender differences in the effects of early intervention: A reevaluation of the abecedarian, perry preschool, and early training projects. *Journal of the American statistical Association*, 2012.

Benjamin F Arnold, Sebastian Galiani, Pavani K Ram, Alan E Hubbard, Bertha Briceño, Paul J Gertler, and John M Colford. Optimal recall period for caregiver-reported illness in risk factor and intervention studies: a multicountry study. *American journal of epidemiology*, 177(4):361–370, 2013.

Nava Ashraf, James Berry, and Jesse M. Shapiro. Can higher prices stimulate product use? evidence from a field experiment in zambia. *American Economic Review*, 100(5):2383–2413, 2010. doi: 10.1257/aer.100.5.2383.

Tin Aung, Willi McFarland, Hnin Su Su Khin, and Dominic Montagu. Incidence of pediatric diarrhea and public–private preferences for treatment in rural myanmar: a randomized cluster survey. *Journal of tropical pediatrics*, 59(1):10–16, 2013.

Tin Aung, Dominic Montagu, Hnin Su Su Khin, Zaw Win, Ang Kyaw San, Willi McFarland, ORS+ Zinc Study Group, et al. Impact of a social franchising program on uptake of oral rehydration solution plus zinc for childhood diarrhea in myanmar: a community-level randomized controlled trial. *Journal of tropical pediatrics*, 60(3):189–197, 2014.

Phyllis Awor, Henry Wamani, Thorkild Tylleskar, George Jagoe, and Stefan Peterson. Increased access to care and appropriateness of treatment at private sector drug shops with integrated management of malaria, pneumonia and diarrhoea: a quasi-experimental study in uganda. *PLoS One*, 9(12):e115440, 2014.

Abhijit Vinayak Banerjee, Esther Duflo, Rachel Glennerster, and Dhruva Kothari. Improving immunisation coverage in rural india: clustered randomised controlled evaluation of immunisation campaigns with and without incentives. *Bmj*, 340:c2220, 2010.

Abdullah H Baqui, Robert E Black, Shams El Arifeen, Mohammad Yunus, K Zaman, Nazma Begum, Amira A Roess, and Mathuram Santosham. Zinc therapy for diarrhoea increased the use of oral rehydration therapy and reduced the use of antibiotics in bangladeshi children. *Journal of Health, Population and Nutrition*, pages 440–442, 2004.

Nita Bhandari, Sarmila Mazumder, Sunita Taneja, Brinda Dube, RC Agarwal, Dilip Mahalanabis, Olivier Fontaine, Robert E Black, and Maharaj K Bhan. Effectiveness of zinc supplementation plus oral rehydration salts compared with oral rehydration salts alone as a treatment for acute diarrhea in a primary care setting: a cluster randomized trial. *Pediatrics*, 121(5):e1279–e1285, 2008.

Zulfiqar A Bhutta, Sheila M Bird, Robert E Black, Kenneth H Brown, et al. Therapeutic effects of oral zinc in acute and persistent diarrhea in children in developing countries: pooled analysis of randomized controlled trials. *The American journal of clinical nutrition*, 72(6): 1516–1522, 2000.

Richard A. Cash, David R. Nalin, Roger Rochat, L. Barth Reller, Zahedul A. Haque, and A. S. M. Mizanur Rahman. A clinical trial of oral therapy in a rural cholera-treatment center. *The American Journal of Tropical Medicine and Hygiene*, 19(4):653–656, 1970.

Jessica Cohen and Pascaline Dupas. Free distribution or cost-sharing? evidence from a randomized malaria prevention experiment. *The Quarterly Journal of Economics*, 125(1):1–45, 2010.

Jai K Das, Zohra S Lassi, Rehana A Salam, and Zulfiqar A Bhutta. Effect of community based interventions on childhood diarrhea and pneumonia: uptake of treatment modalities and impact on mortality. *BMC Public Health*, 13(3):1, 2013.

Stefano DellaVigna. Psychology and economics: Evidence from the field. *Journal of Economic literature*, 47(2):315–372, 2009.

Pascaline Dupas. Health behavior in developing countries. *Annu. Rev. Econ.*, 3(1):425–449, 2011.

Pascaline Dupas, Vivian Hoffmann, Michael Kremer, and Alix Zwane. Short-term subsidies, lasting adoption? habit formation in point-of-use water purification in kenya. *Unpublished manuscript*, 2011.

Pascaline Dupas, Vivian Hoffmann, Michael Kremer, and Alix Peterson Zwane. Targeting health subsidies through a nonprice mechanism: A randomized controlled trial in kenya. *Science*, 353(6302):889–895, 2016.

Birger Carl Forsberg, Max G Petzold, Gran Tomson, and Peter Allebeck. Diarrhoea case management in low- and middle-income countries: an unfinished agenda. *Bulletin of the World Health Organization*, 85:42–48, 2007.

Willa Friedman, Benjamin Woodman, and Minki Chatterji. Can mobile phone messages to

drug sellers improve treatment of childhood diarrhoea?a randomized controlled trial in ghana. *Health policy and planning*, 30(suppl 1):i82–i92, 2015.

Sethson Kassegne, Megan B Kays, and Jerome Nzohabonayo. Evaluation of a social marketing intervention promoting oral rehydration salts in burundi. *BMC public health*, 11(1):1, 2011.

M Kremer and E Miguel. The illusion of sustainability. *Quarterly Journal of Economics*, 122 (3), 2007.

Michael Kremer, Rachel Glennerster, et al. Improving health in developing countries. *Handbook of Health Economics*, 2:201–315, 2011a.

Michael Kremer, Jessica Leino, Edward Miguel, and Alix Peterson Zwane. Spring cleaning: Rural water impacts, valuation, and property rights institutions. *The Quarterly journal of economics*, 126(1):145–205, 2011b.

Michael Kremer, Edward Miguel, Sendhil Mullainathan, Clair Null, and Alix Peterson Zwane. Social engineering: Evidence from a suite of take-up experiments in kenya. *Work. Pap., Univ. Calif., Berkeley*, 2011c.

Lancet. Water with sugar and salt. *Lancet*, 2(8084):300–1, 1978.

Lindsey M Lenters, Jai K Das, and Zulfiqar A Bhutta. Systematic review of strategies to increase use of oral rehydration solution at the household level. *BMC public health*, 13(Suppl 3):S28, 2013.

Ruth Levine. *Millions saved: proven successes in global health*, volume 3. Peterson Institute, 2004.

Li Liu, Hope L. Johnson, Simon Cousens, Jamie Perin, Susana Scott, Joy E. Lawn, Igor Rudan, Harry Campbell, Richard Cibulskis, Mengying Li, Colin Mathers, and Robert E. Black. Global, regional, and national causes of child mortality: an updated systematic analysis for 2010 with time trends since 2000. *The Lancet*, 379(9832):2151–2161, 2012.

Li Liu, Shefali Oza, Daniel Hogan, Jamie Perin, Igor Rudan, Joy E Lawn, Simon Cousens, Colin Mathers, and Robert E Black. Global, regional, and national causes of child mortality in 2000–13, with projections to inform post-2015 priorities: an updated systematic analysis. *The Lancet*, 385(9966):430–440, 2015.

David McKenzie. Beyond baseline and follow-up: The case for more t in experiments. *Journal of Development Economics*, 99(2):210–221, 2012.

Manoj Mohanan, Marcos Vera-Hernández, Veena Das, Soledad Giardili, Jeremy D Goldhaber-Fiebert, Tracy L Rabin, Sunil S Raj, Jeremy I Schwartz, and Aparna Seth. The know-do gap in quality of health care for childhood diarrhea and pneumonia in rural india. *JAMA pediatrics*, 169(4):349–357, 2015.

Melinda K. Munos, Christa L Fischer Walker, and Robert E Black. The effect of oral rehydration solution and recommended home fluids on diarrhoea mortality. *International Journal of Epidemiology*, 39(suppl 1):i75–i87, 2010.

World Health Organization et al. *Programme for Control of Diarrhoeal Diseases: Seventh Programme Report 1988-1989*. The Organization, 1990.

Birte Pantenburg, Theresa J. Ochoa, Lucie Ecker, and Joaquim Ruiz. Use of commercially available oral rehydration solutions in lima, peru. *The American Journal of Tropical Medicine and Hygiene*, 86(6):922–924, 2012.

N. F. Pierce, R. B. Sack, R. C. Mitra, J. G. Banwell, K. L. Brigham, D. S. Fedson, and A. Mondal. Replacement of water and electrolyte losses in cholera by an oral glucose-electrolyte solution. *Annals of internal medicine*, 70(6):1173–1181, 1969.

PSI. What is social marketing? *Population Services International*, 2003. available online at http://www.psi.org/resources/pubs/what is smEN.pdf.

Pavani Kalluri Ram, Misun Choi, Lauren S Blum, Annah W Wamae, Eric D Mintz, and Alfred V Bartlett. Declines in case management of diarrhoea among children less than five years old. *Bulletin of the World Health Organization*, 86:E–F, 2008.

K Vaninadha Rao, Vinod K Mishra, and Robert D Retherford. Knowledge and use of oral rehydration therapy for childhood diarrhoea in india: effects of exposure to mass media. 1998.

Mathuram Santosham. Oral rehydration therapy of infantile diarrhea a controlled study of well-nourished children hospitalized in the united states and panama. *The New England journal of medicine*, 306(18):1070–1076, 1982.

Mathuram Santosham, Aruna Chandran, Sean Fitzwater, Christa Fischer-Walker, Abdullah H. Baqui, and Robert Black. Progress and barriers for the control of diarrhoeal disease. *The Lancet*, 376(9734):63–67, 2010.

Ian Smillie. *Freedom from want: The remarkable success story of BRAC, the global grassroots organization that's winning the fight against poverty.* Kumarian Press, 2009.

Neeraj Sood and Zachary Wagner. Private sector provision of oral rehydration therapy for child diarrhea in sub-saharan africa. *The American Journal of Tropical Medicine and Hygien*, (Forthcoming), 2013.

Philip R. Spandorfer, Evaline A. Alessandrini, Mark D. Joffe, Russell Localio, and Kathy N. Shaw. Oral versus intravenous rehydration of moderately dehydrated children: A randomized, controlled trial. *Pediatrics*, 115(2):295–301, 2005.

Dean Spears. Bounded rationality as deliberation costs: Theory and evidence from a pricing field experiment in india. *Available at SSRN 1444912*, 2009.

Richard Thaler. Toward a positive theory of consumer choice. *Journal of Economic Behavior & Organization*, 1(1):39–60, 1980.

Rebecca L Thornton. The demand for, and impact of, learning hiv status. *The American economic review*, 98(5):1829–1863, 2008.

UDHS. Uganda demographic and health survey 2011 [dataset]. *Uganda Bureau of Statistics [Uganda] and ICF International*, 2011.

USAID. Diarrhoea treatment guidelines: Including new recommendations for the use of ors and zinc supplementation for clinic-based healthcare workers. Technical report, USAID Micronutrient Program, 2005.

Cesar G. Victora, Jennifer Bryce, Olivier Fontaine, and Roeland Monasch. Reducing deaths from diarrhoea through oral rehydration therapy. Technical report, World Health Organization, 2000.

Zachary Wagner, Manan Shah, and Neeraj Sood. Barriers to use of oral rehydration salts for child diarrhea in the private sector: evidence from india. *Journal of tropical pediatrics*, page fmu063, 2014.

Justin White and William H Dow. Intertemporal choices for health. *Behavioral Economics and Public Health*, 2015.

14 Tables

Table 1: Balance Between Groups (Exogenous Variables Assessed at Endline)

	(1)<br>Ctrl Mean	(2)<br>Grp 2-Ctrl	(3)<br>Grp 2-Grp 3	(4)<br>Grp 2-Grp 4
**Caretaker Characteristics**				
Caretaker Age				
Education:				
None				
Primary				
Secondary+				
Employed (Last 7 Days)				
Number of Children				
**Child Characteristics**				
Gender				
Age (months)				
Birth order				
Diarrhea Frequency				
Diarrhea Last 4 Weeks				
Blood In Stool				
Concurrent Fever				
**Household Characteristics**				
Water Source:				
Pipe				
Protected Well/Borehole				
Unprotected Well				
Surface Water				
Main Income:				
Agriculture				
Public Sector				
Private Sector				
Self-employed/informal				

Includes only post-intervention data for households with a case of diarrhea
Sample is identical to main analysis sample
$***p < .01, **p < .05, *p < .1$
Statistical differences assessed using t-tests
Village Clustered SEs in parentheses
Unit of observation=Child

Table 2: Balance Between Groups (Endogenous Variables Assessed at Baseline)

	(1) Ctrl Mean	(2) Grp 2-Ctrl	(3) Grp 2-Grp 3	(4) Grp 2-Grp 4
**Diarrhea Treatment**				
Used ORS for recent case				
Used zinc+ORS for recent case				
Used antibiotic for recent case				
Time to ORS use				
ORS same day as initiation				
Ever used ORS				
Ever used zinc				
ORS stored in home				
**Knowledge**				
Heard of ORS				
Heard of zinc				
**Access**				
Free ORS in village				
Free zinc in village				
**CHP Contact**				
Visited by CHP in past 4 weeks				

Includes only pre-intervention data

$***p < .01, **p < .05, *p < .1$

Village Clustered SEs in parentheses

Unit of observation=Child

Table 3: Intermediate Outcomes

	Ctrl Mean	Grp 2-Ctrl	Grp 2-Grp 3	Grp 2-Grp 4
**Intermediate ORS Outcomes**				
Obtained ORS[1]				
Obtained free ORS[1]				
Obtained ORS at home[1]				
ORS stored: currently[1]				
ORS stored: diarrhea initiation				
**Intermediate Zinc Outcomes**				
Obtained zinc[1]				
Obtained free zinc[1]				
Obtained zinc at home [1]				
Zinc stored: currently[1]				
Zinc stored: last 4 weeks				
Zinc stored: diarrhea initiation				
**Intermediate CHP Outcomes**				
Home visit from CHP				
Visited CHP's home				
Visited health provider for treatment				

[1]Assessed for entire sample, not just those with diarrhea episode

$***p < .01, **p < .05, *p < .1$

Village Clustered SEs in parentheses, adjusted for multiple outcomes

Unit of observation=Caretaker

Table 4: Impact On ORS Use

	ORS Use	
	Unadjusted	Adjusted
Group 2-Control		
Group 2-Group 3		
Group 2-Group 4		
Controls	No	Yes
Control Mean		
Obs		

$***p < .01, **p < .05, *p < .1$
Village Clustered SEs in parentheses
Unadjusted = Equation 1; Adjusted = Equation 2
Covariates for adjusted model described in section 7.4.1
Unit of observation=Child

Table 5: Impact On Secondary Treatment Outcomes

	Zinc+ORS		Antibiotics	
	Unadjusted	Adjusted	Unadjusted	Adjusted
Group 2-Control				
Group 2-Group 3				
Group 2-Group 4				
Controls	No	Yes	No	Yes
Control Mean				
Obs				

$***p < .01, **p < .05, *p < .1$

Village Clustered SEs in parentheses

SEs adjusted for multiple outcomes as described section 7.8

Unadjusted = Equation 1; Adjusted = Equation 2

Covariates for adjusted model described in section 7.4.1

Unit of observation=Child

Table 6: Impact On Time to ORS Use After Diarrhea Initiation

	ORS Same Day (LMP)		Days to ORS (Cox PHM)	
	Unadjusted	Adjusted	Unadjusted	Adjusted
Group 2-Control				
Group 2-Group 3				
Group 2-Group 4				
Controls	No	Yes	No	Yes
Control Mean				
Obs				

$***p < .01, **p < .05, *p < .1$

Village Clustered SEs in parentheses

SEs adjusted for multiple outcomes

LMP=Linear Probability Model; PHM=Proportional Hazard Model

Unadjusted = Equation 1; Adjusted = Equation 2

Covariates for adjusted model described in section 7.4.1

Unit of observation=Child

Table 7: Impact of Home Storage: 2SLS

	ORS Use			
	First Stage	Second Stage	First Stage	Second Stage
Group 2				
Storage				
Controls	No	No	Yes	Yes
F-Stat				
Obs				

$***p < .01, **p < .05, *p < .1$
Village Clustered SEs in parentheses
SEs adjusted for multiple outcomes
Columns 1 and 3=Equation 3, Columns 2 and 4=Equation 4
Covariates for adjusted model described in section 7.4.1
Unit of observation=Child

Table 8: Heterogeneous Treatment Effects: Access

	Outcome = ORS use	
	(1)	(2)
Group 2		
Group 3		
Group 4		
Distance		
Group 2 X Dist		
Group 3 X Dist		
Group 4 X Dist		
Controls	No	Yes
Obs		

$***p < .01, **p < .05, *p < .1$
Village Clustered SEs in parentheses
SEs adjusted for multiple outcomes
Estimated using equation 5
Unit of observation=Child

Table 9: Heterogeneous Treatment Effects: Child Vulnerability

	Outcome = ORS use			
	(1)	(2)	(3)	(4)
Group 2				
Group 3				
Group 4				
Age< 1				
Group 2 X Age< 1				
Group 3 X Age< 1				
Group 4 X Age< 1				
Severe				
Group 2 X Severe				
Group 3 X Severe				
Group 4 X Severe				
Controls	No	Yes	No	Yes
Obs				

$***p < .01, **p < .05, *p < .1$
Village Clustered SEs in parentheses
SEs adjusted for multiple outcomes
Estimated using equation 5
Unit of observation=Child

Table 10: Targeting ORS Subsidies (Take-Up and Use)

	Means (95% Confidence Intervals)		
	ORS Take-Up[1] (α)	ORS Use[2] (μ)	Ratio (λ)
Control			
Group 2			
Group 3			
Group 4			
	Linear Probability Models (Equation 2)		
	ORS Take-Up	ORS Use	
Group 2-Control			
Group 2-Group 3			
Group 2-Group 4			
Controls	Yes	Yes	
Obs			

[1]ORS take-up = 1 if obtained from CHP in last 4-weeks

[2]ORS use = 1 if use ORS to treat child diarrhea in last 4-weeks

Sample includes only households with diarrhea episode

Unit of observation=Caretaker

Table 11: Targeting ORS Subsidies (Lost Packets and Non-Child Use)

	Means (95% Confidence Intervals)	
	Lost packet[1]	Used for non-child[2]
Control		
Group 2		
Group 3		
Group 4		
	Linear Probability Models (Equation 1)	
	Lost packet	Used for non-child
Group 2-Control		
Group 2-Group 3		
Group 2-Group 4		
Controls	Yes	Yes
Obs		

[1]ORS take-up = 1 if obtained from CHP in last 4-weeks
[2]ORS use = 1 if use ORS to treat child diarrhea in last 4-weeks
[1]Sample includes only households with diarrhea episode
[2]Sample includes only households with diarrhea episode
Unit of observation=Caretaker

Table 12: Cost-Effectiveness Analysis

	Costs and DALYs Averted				
	Cost	Incr Cost	DALYs Averted	Incr DALYs Averted	ICER
Control		N/A		N/A	N/A
Group 2					
Group 3					
Group 4					

Costs include both ORS costs and cost of time for CHP and caretaker
Incremental costs are relative to control

Table 13: Impact On ORS Use (Packet Counting)

	ORS Use	
	Unadjusted	Adjusted
Group 2-Control		
Controls	No	Yes
Control Mean		
Obs		

$***p < .01, **p < .05, *p < .1$
Village Clustered SEs in parentheses
Unadjusted = Equation 1; Adjusted = Equation 2
Covariates for adjusted model described in section 7.4.1

Table 14: Placebo Tests (Health Behaviors That Should Not Be Affected)

	Malaria Treatment	Unclean Water/Food	Always Bednet	Hand Washing
Group 2-Control				
Group 2-Group 3				
Group 2-Group 4				
Controls	Yes	Yes	Yes	Yes
Control Mean				
Obs				

$***p < .01, **p < .05, *p < .1$

Village Clustered SEs in parentheses

Controls described in section 7.4.1

Malaria Treatment=child given malaria treatment in last 4 weeks (if malaria symptoms)

Unclean Water/Food=child given unclean water or food in a last 4 weeks

Always Bednet=child "always" slept under a bed net during last 4-weeks

Hand Washing=child washed hands at least once per day in last 4 weeks

Table 15: Impact On ORS Use (Shorter Recall Periods)

	ORS Use	
	7-days	Current Case
Group 2-Control		
Group 2-Group 3		
Group 2-Group 4		
Controls	Yes	Yes
Control Mean		
Obs		

$***p < .01, **p < .05, *p < .1$

Village Clustered SEs in parentheses

Controls described in section 7.4.1

7-Days implies case ended within 7 days

Current case implies case at time of survey

15 Appendix

15.1 Appendix 1: Appendix Tables

Table 16: Balance Between Groups (Summary Statistics-Exogenous Variables)

	(1)<br>Control	(2)<br>Group 2	(3)<br>Group 3	(4)<br>Group 4	(5)<br>χ^2
**Caretaker Characteristics**					
Caretaker Age					
Education:					
None					
Primary					
Secondary+					
Employed (Last 7 Days)					
Number of Children					
**Child Characteristics**					
Age (months)					
Birth order					
Diarrhea Frequency					
Diarrhea Last 4 Weeks					
**Household Characteristics**					
Water Source:					
Pipe					
Protected Well/Borehole					
Unprotected Well					
Surface Water					
Main Income:					
Agriculture					
Public Sector					
Private Sector					
Self-employed/informal					

Includes only post-intervention data for households with a case of diarrhea
Sample is identical to main analysis sample
$***p < .01, **p < .05, *p < .1$
χ^2 estimated using multinomial logit with treatment at dependent variable
Standard Deviations in parentheses
Unit of observation=Child

Table 17: Balance Between Groups (Summary Statistics - Endogenous Variables Assessed at Baseline)

	(1) Control	(2) Group 2	(3) Group 3	(4) Group 4	(5) χ^2
**Diarrhea Treatment**					
Used ORS for recent case					
Used zinc for recent case					
Used zinc+ORS for recent case					
Used antibiotic for recent case					
Ever used ORS					
Ever used zinc					
Ever used antibiotic					
**Knowledge**					
Heard of ORS					
Heard of zinc					
**Access**					
Free ORS in village					
Free zinc in village					
Time to ORS distributor					
Time to zinc distributor					
**CHP Contact**					
Visited by CHP in past 4 weeks					
Ever visited by CHP					

Includes only pre-intervention data

$***p < .01, **p < .05, *p < .1$

χ^2 estimated using multinomial logit with treatment at dependent variable

Standard Deviations in parentheses

Unit of observation=Child

Table 18: Impact On ORS Use (Full Regression Results)

	ORS Use	
	Unadjusted	Adjusted
Group 2-Control		
Group 2-Group 3		
Group 2-Group 4		
**Caretaker Characteristics**		
Age (years)		
Education:		
None		
Primary		
Secondary+		
Number of Children		
**Child Characteristics**		
Age (months)		
Diarrhea Frequency		
**Household Characteristics**		
Water Source:		
Pipe		
Protected Well/Borehole		
Unprotected Well		
Surface Water		
**Baseline Village Characteristics:**		
%ORS use		
%Visited by CHP last month		
%Aware of free ORS		
Minutes to nearest ORS		
%ORS Stored in Home		
Control Mean		
Obs		
R^2		

$***p < .01, **p < .05, *p < .1$

Village Clustered SEs in parentheses

Unadjusted = Equation 1; Adjusted = Equation 2

Unit of observation=Child

Table 19: Impact On Secondary Treatment Outcomes (Full Regression Results)

	Zinc + ORS	Antibiotics
Group 2-Control		
Group 2-Group 3		
Group 2-Group 4		
**Caretaker Characteristics**		
Age (years)		
Education:		
None		
Primary		
Secondary+		
Number of Children		
**Child Characteristics**		
Age (months)		
Diarrhea Frequency		
**Household Characteristics**		
Water Source:		
Pipe		
Protected Well/Borehole		
Unprotected Well		
Surface Water		
**Baseline Village Characteristics:**		
%ORS use		
%Visited by CHP last month		
%Aware of free ORS		
Minutes to nearest ORS		
%ORS Stored in Home		
Control Mean		
Obs		
R^2		

$***p < .01, **p < .05, *p < .1$

Village Clustered SEs in parentheses

Unadjusted = Equation 1; Adjusted = Equation 2

Covariates for adjusted model described in section 7.4.1

Unit of observation=Child

Table 20: Impact On Time to ORS and Zinc Use (Full Regression Results)

	Time to ORS		Time to Zinc+ORS	
	LMP	Cox PHM	LMP	Cox PHM
Group 2-Control				
Group 2-Group 3				
Group 2-Group 4				
**Caretaker Characteristics**				
Age (years)				
Education:				
None				
Primary				
Secondary+				
Number of Children				
**Child Characteristics**				
Age (months)				
Diarrhea Frequency				
**Household Characteristics**				
Water Source:				
Pipe				
Protected Well/Borehole				
Unprotected Well				
Surface Water				
**Baseline Village Characteristics:**				
%ORS use				
%Visited by CHP last month				
%Aware of free ORS				
Minutes to nearest ORS				
%ORS Stored in Home				
Control Mean				
Obs				
R^2				

$***p < .01, **p < .05, *p < .1$

Village Clustered SEs in parentheses

Unadjusted = Equation 1; Adjusted = Equation 2

Covariates for adjusted model described in section 7.4.1

Unit of observation=Child

15.2 Appendix 2: Survey Instrument

ENGLISH VERSION – DIARRHEA MODULES

2016 UGANDA DIARRHEA PREVENTION AND TREATMENT RESEARCH

TARGET: CAREGIVERS OF CHILDREN BETWEEN 0 AND 59 MONTHS WITH DIARRHEA IN PAST 4 WEEKS

Confidential: Data used for research purposes only

IDENTIFICATION
**HOUSEHOLD UNIQUE ID:** |___| |___|___|___|___|___|___|___| |___|___|

**REGION:** ____________________________	|__|__|
**DISTRICT:** ____________________________	|__|__|
**COUNTY :** ____________________________	|__|__|
**SUBCOUNTY/TOWN :** ____________________________	|__|__|
**PARISH:** ____________________________	|__|__|
**LCI NAME:** ____________________________	|__|__|
**ENUMERATION AREA:** ____________________________	|__|__|
**AREA (URBAN=1; RURAL=2) :**____________________________	|__|
**NAME OF HEAD OF HOUSEHOLD:** ____________________________	

INTERVIEWER'S VISITS				
	1	2	3	**FINAL VISIT**
DATE	________	________	________	**DAY** |__|__| **MONTH** |__|__| **YEAR** |2|0|1|6|
INTERVIEWER'S NAME	________	________	________	**INITIAL** |__|__|
RESULT*	________	________	________	**RESULT*** |__|__|
NEXT VISIT : DATE	________	________		TOTAL NBR OF VISITS |__|
TIME	________	________		

TO BE FILLED BY SUPERVISOR :	
TOTAL MAIN CAREGIVERS OF CHILDREN AGED 0-59 MONTHS WITH DIARRHEA IN PAST 4 WEEKS IN HOUSEHOLD (FORM A)	|__|__|
TOTAL CHILDREN AGED 0-59 MONTHS WITH DIARRHEA IN PAST 4 WEEKS (FORM B)	|__|__|

FIELD SUPERVISOR:	QUALITY CONTROLLER:	DATA ENTRY:
NAME______________/___/___/	NAME ______________/___/___/	NAME ______________/___/___/

*CODES FOR RESULT
1= Completed
2= No HH member at home/no competent respondent
3= Entire HH absent for extended period
4= Refused to be interviewed
5= Was not at home
6= Dwelling vacant/address not a dwelling
7= Deaf/Did not speak a survey language
8= No adults in household
9= Interview postponed
10= Interview partially completed
11= Other (specify)____________________

START TIME /___/___/___/___/

FORM A.

CAREGIVER SELECTION TOOL FOR USE WITHIN HOUSEHOLDS SELECTED FOR DIARRHEA MODULE

1. **What is the total number of MAIN caregivers of children aged 0-59 months that are presently home? A MAIN caregiver is the person that makes decisions about how to care for the children in your household. _____**

2. Please provide the First name and Last initial of all these MAIN caregivers *(Interviewer: list in second column below):*

Serial no.	First name, Last initial<br>(all MAIN caregivers of children 0-59 months and with diarrhea in past 4 weeks)
1	
2	
3	
4	
5	

RANDOM SELECTION (if 2 MAIN caregivers or more are listed in table above):

Interviewer: Take out your phone and use the random number generator application. Set the Min to 1 and the Max to the number of caretakers in the household. Press 'generate' and select the caretaker that corresponds to the number that is generated.

3. The person I need to speak to is _____________ *(insert the first name and last initial)*. May I please talk to this person now?

   Yes...........1 ***[Interviewer: move to informed consent]***

   Refuse.........2 ***[Interviewer: Thank the respondent and move to next eligible household]***

4. Were you or someone from your household asked to complete a questionnaire about your children's health about 4 weeks ago?

INTRODUCTION AND ORAL CONSENT

Good morning/afternoon. My name is _____________. I am a researcher working for Makerere University and the University of California, Berkeley in the United States. We are conducting a survey on child health and treatment practices among the residents of Uganda. We are inviting you to participate in this study because you have at least one child under 5-years-old living in your household. This information will be used to inform programming efforts by the Uganda Ministry of Health and other organizations that focus on diarrhea treatment in the country.

PROCEDURES

If you agree to take part, some of the questions that we ask will be about health practices and diarrhea treatment. We will interview you in a private place. The interview will take no more than 30 minutes to complete. To further protect your privacy, your name will not appear on any questionnaire. The answers we collect from you will not be shown to anyone outside of the study team.

RISKS/DISCOMFORTS

It is possible that some of the research questions may make you uncomfortable or upset. You are free to decline to answer any questions you don't wish to, or to stop the interview at any time. As with all research, there is a chance that confidentiality could be compromised; however, we will do everything we can to make sure that this does not happen. Whatever information you provide will be kept strictly confidential and will not be shown to other persons. The answers you give will not be shared with anyone outside of the study team.

BENEFITS

There is no direct benefit to you from being in this study. However, the information we collect will help develop better programs and health services for people in Uganda.

CONFIDENTIALITY

Your study data will be handled as confidentially as possible. If results of this study are published or presented, individual names and other personally identifiable information will not be used.
To minimize the risks to confidentiality, we will do the following:

- The data will be collected anonymously. We will not maintain a link between your identity and the research data. Personal identifiers will be removed as soon as data is entered into our computers.
- Your research records will be stored on a password-protected computer
- Only I/the researcher(s) will have access to your study records.

FUTURE USE OF STUDY DATA

The research data will be maintained for possible use in future research by the study team.

COMPENSATION/PAYMENT

You will receive a bar of soap for your participation in this study.

VOLUNTARY PARTICIPATION

You do not have to agree to be in this study, and you may change your mind at any time without penalty or loss of benefits to which you are otherwise entitled.

QUESTIONS

If you have any questions about this study, you may call Dr. John Bosco Asiimwe at Makerere University at 772-428-489. He will answer any questions or address any concerns you may have. If you have any questions about your rights as a study participant, or if you think you have not been treated fairly, you may call the National Council for Science and Technology, telephone 0-414 250 499.

PERMISSION TO PROCEED

You have been given a copy of this consent form to keep.

Do you have any questions about the survey? Yes/No

Do I have permission to interview you now? Yes / No

Interviewer: If no, thank the respondent and end the questionnaire. Indicate Result in identification table.

______________________	______________________________	__________
Print name of Person Obtaining Consent	Signature of Person Obtaining Consent	Date

FORM B.

CHILD SELECTION TOOL FOR USE WITHIN HOUSEHOLDS SELECTED <u>FOR DIARRHEA MODULE</u>

1. **Interviewer, ask respondent: "Do you have ORS stored in your household?"**

Yes…………1
No…………..2

2. **Interviewer, ask respondent: "Have any of your children under 5 years old had diarrhea in the past 4 weeks"**

Yes…………1 **(Continue to 3)**
No…………..2 **(Skip to Form C.)**

3. **What is the total number of children under 5 years old with diarrhea in the last 4 weeks for whom you are responsible: _____**

4. Please provide the First name of all these children *(Interviewer: list in second column below)*

Serial no.	First name<br>(children 0-59 months with diarrhea in the past 4 weeks)
1	
2	
3	
4	
5	

5. We will be discussing the health of these children in the interview today.

HOUSEHOLD QUESTIONNAIRE: SOCIOECONOMIC MODULE

Section 1: Socioeconomic Module				
**No**	**Questions and Filters**	**Responses**	**Codes**	**Skip To**
101	How old are you?		[ ][ ]	
102	What is the **highest** level of school you attended?	None/ Nursery<br>Primary<br>Vocational<br>Secondary<br>Tertiary/College (Middle level)<br>University	1<br>2<br>3<br>4<br>5<br>6	
103	What is your marital status?	Never married<br>Married/Living together<br>Widowed<br>Divorced<br>Separated	1<br>2<br>3<br>4<br>5	
104	a. How many children do you have?<br><br>b. How many of these children are under 5-years-old?	[ ][ ] Children<br><br>[ ][ ] Children		
105	Which of the following items are available in this household?<br><br>***Interviewer: Read list. Multiple responses allowed.***	Electricity<br>Radio<br>Television<br>Refrigerator<br>Cell phone/Mobile<br>Landline phone<br>Gas/electric cooker<br>Bicycle<br>Sofa set<br>Water tank<br>NONE OF THE ABOVE	1<br>2<br>3<br>4<br>5<br>6<br>7<br>8<br>9<br>10<br>11	
106	MAIN MATERIAL OF FLOOR<br><br>***(Interviewer: Record observation. If interview is not inside house, ask to see inside)***	NATURAL FLOOR<br>Earth/Sand<br>Earth and Dung<br>FINISHED FLOOR<br>Stones<br>Bricks<br>Parquet or Polished Wood<br>Mosaic or tiles<br>Cement<br>Other (Specify):<br>____________________	 1<br>2<br><br>3<br>4<br>5<br>6<br>7<br>99	

107	MAIN MATERIAL OF WALL<br><br>***(Interviewer: Record observation)***	NATURAL WALLS<br>Thatched/Straw<br>RUDIMENTARY WALLS<br>Mud and poles<br>Un-burnt bricks<br>Un-burnt bricks with plaster<br>Burnt bricks with mud<br>FINISHED WALLS<br>Cement blocks<br>Stone<br>Timber<br>Burnt bricks with cement<br>Other (Specify):<br>____________________	 1<br><br>2<br>3<br>4<br>5<br><br>6<br>7<br>8<br>9<br>99	
108	MAIN MATERIAL OF ROOF<br><br>***(Interviewer: Record observation)***	NATURAL ROOFING<br>Thatched<br>Mud<br>FINISHED ROOFING<br>Wood/planks<br>Iron sheets<br>Asbestos<br>Tiles<br>Tin<br>Cement<br>Other (specify):<br>____________________	 1<br>2<br><br>3<br>4<br>5<br>6<br>7<br>8<br>99	
109	What type of toilet does your household use **most** of the time?	Flush toilet<br>VIP latrine<br>Covered pit latrine no slab<br>Covered pit latrine w/ slab<br>Uncovered pit latrine no slab<br>Uncovered pit latrine w/slab<br>Composting toilet<br>Bush<br>Other (Specify):<br>____________________	1<br>2<br>3<br>4<br>5<br>6<br>7<br>8<br>99	

110	What is the main source of drinking water for the members of your household?	PIPED WATER<br>Piped - into house<br>Piped to yard/plot<br>Public tap/standpipe<br>WATER FROM OPEN WELL/SPRING<br>Open well/spring in yard/plot<br>Open public well/spring<br>WATER FROM PROTECTED WELL/SPRING<br>Protected well/spring in yard/plot<br>Protected public well/spring<br>WATER FROM BOREHOLE<br>Borehole in yard/plot<br>Public borehole<br>SURFACE WATER (RIVER/DAM ETC)<br>River/stream<br>Pond/lake<br>Dam<br>Rain water<br><br>Tanker truck<br>Vendor<br>Other (Specify):<br>________________	 1<br>2<br>3<br><br>4<br>5<br><br>6<br>7<br><br>8<br>9<br><br>10<br>11<br>12<br>13<br><br>14<br>15<br>99	
111	How much time does it take you to obtain drinking water (round trip)?	Water on premises<br>Less than 30 minutes<br>30 minutes or longer<br>Don't know	1<br>2<br>3<br>88	
112	What type of cooking fuel does your household use **most** of the time?	Fire wood<br>Charcoal<br>Kerosene/paraffin<br>Gas/Biogas/LPG<br>Electricity<br>Straw/shrubs/grass<br>No food cooked in the household<br>Other (Specify):<br>________________	1<br>2<br>3<br>4<br>5<br>6<br>7<br>99	
113	What is the **main** source of income for the household?	Farming<br>Employment: private sector/NGO<br>Employment: Civil service<br>Self employed/own business<br>Jua kali/informal<br>Casual/contract jobs<br>Spousal support<br>Parental support<br>Domestic work<br>Pension<br>Other (Specify):<br>________________	1<br>2<br>3<br>4<br>5<br>6<br>7<br>8<br>9<br>10<br>99	
114	Does your household own any agricultural land?	Yes<br>No<br>Don't know	1<br>2<br>88	

115	Have you worked to earn income in the last **7 days**?<br><br>***(Interviewer: include both wage and self employment work)***	Yes<br>No	1<br>2	
116	Have you worked to earn income in the last **12 months**?<br><br>***(Interviewer: include both wage and self employment work)***	Yes<br>No	1<br>2	
117	How often do you read the newspaper?	Daily<br>Several times a week<br>Once a week<br>Occasionally<br>Never<br>Don't know	1<br>2<br>3<br>4<br>5<br>88	 <br><br><br>**→Q119**<br>**→Q119**
118	In the **past 1 month**, have you read a newspaper?	Yes<br>No<br>Don't know	1<br>2<br>88	
119	Do you have access to and/or do you use the Internet?	Yes<br>No<br>Don't know	1<br>2<br>88	

END OF SOCIOECONOMIC MODULE

120	Have you heard of a treatment for child diarrhea called Oral Rehydration Salts (ORS)	Yes<br>No	1<br>2 →**Q126**
121	Have you ever used ORS to treat a child's diarrhea	Yes<br>No	1<br>2
122	Is there anywhere in your village where you can go to get oral rehydration salts (ORS) to treat your child's diarrhea?	Yes<br>No	1<br>2 →Q126
123	Where can you go to get oral rehydration salts (ORS) to treat your child's diarrhea?<br><br>**Circle all that apply**	Government Health Center<br>Private Health Center<br>Pharmacy/Drug Shop<br>Village Health Team<br>Community Health Promoter<br>Other_________<br>Don't Know	1<br>2<br>3<br>4<br>5<br>88<br>99
124	Is there anywhere in your village where you can go to get **FREE** oral rehydration salts (ORS)?	Yes<br>No	1<br>2 →Q126
125	Where can you go to get **FREE** oral rehydration salts (ORS)?<br><br>**Circle all that apply**	Government Health Center<br>Private Health Center<br>Pharmacy/Drug Shop<br>Village Health Team<br>Community Health Promoter<br>Other_________<br>Don't Know	1<br>2<br>3<br>4<br>5<br>88<br>99
126	Have you heard of a treatment for child diarrhea called Zinc	Yes<br>No	1<br>2 →132
127	Have you ever used Zinc to treat a child's diarrhea	Yes<br>No	1<br>2
128	Is there anywhere in your village where you can go to get Zinc to treat your child's diarrhea?	Yes<br>No	1<br>2 →132
129	Where can you go to get Zinc to treat your child's diarrhea?<br><br>**Circle all that apply**	Government Health Center<br>Private Health Center<br>Pharmacy/Drug Shop<br>Village Health Team<br>Community Health Promoter<br>Other_________<br>Don't Know	1<br>2<br>3<br>4<br>5<br>88<br>99
130	Is there anywhere in your village where you can go to get **FREE** Zinc?	Yes<br>No	1<br>2 →132
131	Where can you go to get **FREE** Zinc?<br><br>**Circle all that apply**	Government Health Center<br>Private Health Center<br>Pharmacy/Drug Shop<br>Village Health Team<br>Community Health Promoter<br>Other_________<br>Don't Know	1<br>2<br>3<br>4<br>5<br>88<br>99

132	a. How many minutes does it take to travel to the nearest **government health center**?	☐☐ **minutes**	
	b. Have you visited the **government health center** in the past 4 weeks?	Yes<br>No	1<br>2
133	a. How many minutes does it take to travel to the nearest **private health center**?	☐☐ **minutes**	
	b. Have you visited the **private health center** in the past 4 weeks?	Yes<br>No	1<br>2
134	a. How many minutes does it take to travel to the nearest **pharmacy/Drug Shop**?	☐☐ **minutes**	
	b. Have you visited the **Pharmacy/Drug Shop** in the past 4 weeks?	Yes<br>No	1<br>2
135	Is there a **Village Health Team (VHT)** in your village?	Yes<br>No<br>DK	1<br>2 →Q138<br>99 →Q138
136	Do you know where the VHT's household is located?	Yes<br>No	1<br>2 →Q137b
137	a. How many minutes does it take to travel to the house of the **Village Health Team (VHT)**	☐☐ **minutes**	
	b. Have you had a visit with the **Village Health Team (VHT)** in the past 4 weeks?	Yes<br>No	1<br>2
138	Is there a **Community Health Promoter (CHP)** in your village?	Yes<br>No<br>DK	1<br>2 →Section 2<br>99 → Section 2
139	Do you know where the CHP's household is located?	Yes<br>No	1<br>2 →Q140b
140	a. How many minutes does it take to travel to the house of the **Community Health Promoter (CHP)**	☐☐ **minutes**	
	b. Have you had a visit with the **Community Health Promoter (CHP)** in the past 4 weeks?	Yes<br>No	1<br>2

SECTION 2 – DIARRHEA TREATMENT<br>*Interviewer:* Aks Q201-279 for all children 0-59 months who had diarrhea in the past 4 weeks				
**No**	**Questions and Filters**	**Responses**	**Codes**	**Skip To**
201	First name of selected child<br>***Interviewer: check Form B***	First name: ______________________		
202	What is the sex of the child?	Male<br>Female	1<br>2	
203	How old is the child?<br><br>***Interviewer: record age in months***	☐☐ months		
204	What is your relationship with the child?	Mother<br>Grandmother<br>Aunt<br>Sister<br>Other (specify): ____________	1<br>2<br>3<br>4<br>99	
205	How many older siblings does (NAME) have?	☐☐ Siblings		
206	How frequently does (NAME) come down with diarrhea?	At least once per month<br>Once every 2 months<br>Once every 3 months<br>Once every 4 months<br>Less than once every 4 months	1<br>2<br>3<br>4<br>5	
207	Can you confirm that (NAME) had diarrhea in the last 4 weeks?	Yes<br>No	1<br>2	 **→See instructions in footnote[1]**
208	Does the child currently have diarrhea?	Yes<br>No	1<br>2	
209	For how many days has/did the child had diarrhea?	☐☐ days		
210	Has (NAME) also had a fever during this diarrhea episode?	Yes<br>No<br>Don't know	1<br>2<br>88	
211	Did (NAME) have any blood in the stools when he or she had diarrhea in the last 4 weeks?	Yes<br>No<br>Don't know	1<br>2<br>88	
212	How much was (NAME) given to drink during the recent episode of diarrhea?<br><br>***Interviewer: Read list. Mark only one answer***	Much less than usual<br>Somewhat less than usual<br>About the same<br>Somewhat more than usual<br>Much more than usual<br>Nothing to drink<br>Don't know/Don't remember	1<br>2<br>3<br>4<br>5<br>6<br>88	
213	Is (NAME) usually breastfed?	Yes<br>No	1<br>2	 ***→Q215***

[1] This question is a second check to make sure that we did the screening/selection correctly. If the child did have diarrhea according to the screening information, but they say NO diarrhea here, then stop the interview. First, check if the same caregiver has another child 0-59 months with diarrhea in the past 4 weeks and select that child (or randomly select if more than one). If that selected caregiver doesn't have another child in the same age range w/diarrhea, then check if another caregiver in the household has a child w/diarrhea and re-do the child selection with that different caregiver. If there are no more caregivers in the household with a child with diarrhea in past 4 weeks, then stop and move to next household.

214	How much was (NAME) breastfed during the recent episode of diarrhea?<br><br>***Interviewer: Read list. Mark only one answer***	Breastfed less<br>Breastfed about the same<br>Breastfed more<br>Not breastfed at all<br>Too old for breastfeeding<br>Don't know/Don't remember	1<br>2<br>3<br>4<br>5<br>88	
215	How much was (NAME) given to eat during the recent episode of diarrhea? Less than usual to eat, about the same amount, more than usual to eat, or nothing to eat?<br><br>***Interviewer: Read list. Mark only one answer***	Much less than usual<br>Somewhat less than usual<br>About the same<br>Somewhat more than usual<br>Much more than usual<br>Nothing to eat<br>Don't know/Don't remember	1<br>2<br>3<br>4<br>5<br>6<br>88	
216	Did you seek **advice** from someone outside the home for the diarrhea?	Yes<br>No<br>Don't know	1<br>2<br>88	 **→Q220**<br>**→Q220**
217	How many days after the diarrhea began did you first seek advice?<br><br>***Interviewer: If the same day, record '00.'***	[ ][ ] days		
218	Indicate the place where you received the main advice for (NAME)?<br><br>***Interviewer: Do not read list. Mark only one answer.***	**Public Sector**<br>Health center<br>Community distributor (VHT or CHP)<br>Other public sector<br><br>**Private Sector**<br>Private Clinic/provider<br>Private pharmacy/drug store<br>Faith-based, NGO/CBO<br>Friends/Relatives<br>Traditional healer<br>Don't know<br>Other (specify): ______________	 1<br>2<br>3<br><br><br>4<br>5<br>6<br>7<br>8<br>88<br>99	
219	What advice did you receive from this place?<br><br>***Interviewer: Multiple responses allowed. Do not read list.***<br>***Probe: Any other advice?***<br><br>****Note:***<br>• ***Antidiarrheals include products to slow frequency of stools (i.e. Imodium, Lomotil), and bismuth subsalicylate (i.e. Pepto-Bismol)***	Give fluids<br>Give ORS<br>Give zinc<br>Give antibiotic<br>Give antidiarrheal*<br>Give fever medicine<br>Give anti-nausea (vomitting) medicine<br>Give more than usual amount of fluid<br>Give more than usual to eat<br>Continue breastfeeding<br>Take to clinic or hospital<br>Don't know<br>Other (specify): ______________	1<br>2<br>3<br>4<br>5<br>6<br>7<br>8<br>9<br>10<br>11<br>88<br>99	

220	Did you **seek treatment** from someone outside the home for the diarrhea?<br><br>***Interviewer: make sure respondent understands that "treatment" includes medicine, ORS, zinc, etc.***	Yes<br>No<br>Don't know	1<br>2<br>88	 →**Q226**<br>→**Q226**
221	How many days after the diarrhea began did you first seek treatment?<br><br>***Interviewer: If the same day, record '00.'***	☐☐ days		
222	Indicate the first place where you first sought treatment for (NAME)?<br><br>***Interviewer: Mark only one answer.***	**Public Sector**<br>Health center<br>Community distributor (VHT or CHP)<br>Other public sector<br><br>**Private Sector**<br>Private Clinic/provider<br>Private pharmacy/drug store<br>Faith-based, NGO/CBO<br>Friends/Relatives<br>Traditional healer<br>Don't know<br>Other (specify): ______________	 1<br>2<br>3<br><br><br>4<br>5<br>6<br>7<br>8<br>88<br>99	
223	Did request a specific type of treatment or did you let the provider determine the treatment?	Requested Treatment<br>Let Provider Decide<br>Don't know	1<br>2<br>88	 →**Q226**<br>→**Q226**
224	What treatment(s) did you ask for?<br><br>***Interviewer: Do not prompt. Multiple responses allowed***	ORS<br>Restors<br>Zinc<br>Zinkid<br>Antibiotic<br>Antidiarrheal<br>Don't know<br>Other (specify): ______________	1<br>2<br>3<br>4<br>5<br>6<br>88<br>99	
225	Why did you ask for this treatment?<br><br>***Interviewer: Read list. Multiple responses allowed.***	I always use it<br>Most effective<br>Saw it advertised<br>Other (specify): ______________	1<br>2<br>3<br>99	
226	Now, I would like to ask you some questions regarding diarrhea treatment.<br><br>Did you give (NAME) anything to treat the Diarrhea?<br><br>***Interviewer: make sure respondent understands that "treatment" includes medicine, ORS, zinc, solutions, pills, etc…***	Yes<br>No<br>Don't know	1<br>2<br>88	 →**Q228**<br>→**Q228**

227	a. If yes, can you tell me or show me what treatments you gave (NAME) (either home-prepared or from outside of home)<br><br>Interviewer: DO NOT PROMPT. RECORD ALL THAT APPLY.<br><br>b. If yes, can you tell me if you gave your child any of the following treatments<br><br>***Interviewer: READ LIST.***<br><br>***Multiple responses allowed; circle all that apply. If respondent still has medicine package, ask to show.***<br><br>***Note:***<br>• ***<u>Home-prepared treatment</u> include: Sugar Salt Solution, Maize/millet Porridge, Herbal remedies, Passion fruit juice***<br>• ***<u>Antidiarrheals</u> include products to slow frequency of stools (i.e. Imodium, Lomotil), and bismuth subsalicylate (i.e. Pepto-Bismol)***	ORS<br>Zinc<br>Home-prepared treatment<br>Antibiotic<br>Anti-diarrheal*<br>Intravenous fluid<br>Injection<br>Fever medicine<br>Anti-nausea (vomitting) medicine<br>Other pill/syrup<br>Vitamins<br>Don't know<br>Other (specify): ______________	1<br>2<br>3<br>4<br>5<br>6<br>7<br>8<br>9<br>10<br>11<br>88<br>99	
228	Did you have ORS stored in your home when (NAME) started having diarrhea?	Yes<br>No<br>Don't Know	1<br>2<br>88	
229	Did you have Zinc stored in your home when (NAME) started having diarrhea?	Yes<br>No<br>Don't Know	1<br>2<br>88	
230	***Interviewer: check if q227=1: ORS was given to the child.***	**Yes**<br>**No**	**1**<br>**2**	 **→Q241**
231	You mentioned that you have given (NAME) an ORS. Is that correct?<br><br>***Interviewer: If did not give ORS, correct q226 and q227.***	Yes<br>No<br>Don't know	1<br>2<br>88	 **→Q241**<br>**→Q241**
232	How many days after the diarrhea began did you first give (NAME) ORS?<br><br>***Interviewer: If the same day, record '00.'***	[ ][ ] days		

233	From where was the ORS obtained?	**Public Sector**<br>Health center<br>Community distributor (VHT or CHP)<br>Other public sector<br><br>**Private Sector**<br>Private Clinic/provider<br>Private pharmacy/drug store<br>Faith-based, NGO/CBO<br>Friends/Relatives<br>Traditional healer<br>Don't know<br>Other (specify): ______________	 1<br>2<br>3<br><br><br>4<br>5<br>6<br>7<br>8<br>88<br>99	
234	How often did you give the ORS treatment to (NAME)?<br><br>***Interviewer: Read the list and ask the respondent to select one response.***	After each liquid stool<br>Morning, mid-day, and night<br>Whenever the child wanted it<br>Once per day<br>Don't know<br>Other (specify): ______________	1<br>2<br>3<br>4<br>88<br>99	
235	How many packets of ORS did you give to (NAME) during the episode of diarrhea?	Less than 1packet<br>1 packet<br>2 packets<br>3 packets<br>More than 3 packets	1<br>2<br>3<br>4<br>5	
236	How many days did you give the child the ORS?	☐☐ days		
237	Did you use ordinary water or did you use treated or boiled water when you prepared the ORS?	Ordinary (Non Purified) Water<br>Treated Water<br>Boiled Water<br>Other (specify): ______________	1<br>2<br>3<br>99	
238	About how much water did you use for each packet of ORS?	Less than half a liters<br>half a liter<br>1 liter<br>2 liters<br>more than 2 liters<br>Don't know	1<br>2<br>3<br>4<br>5<br>88	
239	Did you purchase the ORS or obtain it free?	Purchased<br>Free<br>Don't know	1<br>2<br>88	 →<br>→
240	What price did you pay for each packet of ORS?	☐☐☐☐ ***UGX***<br>Don't know	 88	
241	***Interviewer: check if q227=2: Zinc was given to the child.***	**Yes**<br>**No**	**1**<br>**2**	 **→Q252**
242	You mentioned that you have given (NAME) Zinc. Is that correct?<br><br>***Interviewer: If did not give Zinc, correct q226 and q226.***	Yes<br>No<br>Don't know	1<br>2<br>88	 **→Q252**<br>**→Q252**
243	How many days after the diarrhea began did you first give (NAME) Zinc?<br><br>***Interviewer: If the same day, record '00.'***	☐☐ days		

244	From where was the Zinc obtained?	**Public Sector**<br>Health center<br>Community distributor (VHT or CHP)<br>Other public sector<br><br>**Private Sector**<br>Private Clinic/provider<br>Private pharmacy/drug store<br>Faith-based, NGO/CBO<br>Friends/Relatives<br>Traditional healer<br>Don't know<br>Other (specify): _____________	 1<br>2<br>3<br><br><br>4<br>5<br>6<br>7<br>8<br>88<br>99	
245	How often did you give the Zinc treatment to (NAME)?<br><br>***Interviewer: Read the list and ask the respondent to select one response.***	After each liquid stool<br>Once per day<br>Morning, mid-day, and night<br>Every other day<br>Whenever the child wanted it<br>Don't know<br>Other (specify): _____________	1<br>2<br>3<br>4<br>5<br>88<br>99	
246	How many tablets were you given/or did you purchase in total?<br><br>*Interviewer: Clarify that this tablet NOT packets and includes tablets received from all sources including neighbors.*	Number<br>Don't know	☐☐<br>88	
247	How many Zinc tablets did you give the child in total?	☐☐ tablets		
248	***INTERVIEWER***: If respondent did not give all the tablets to the child ask to see remaining tablets.<br><br>**RECORD NUMBER OF REMAINING TABLETS**	number of remaining tablet_____		
249	How many days did you give the child the Zinc?	☐☐ days		
250	Did you purchase the zinc or obtain it free?	Purchased<br>Free<br>Don't know	1<br>2<br>88	
251	What price did you pay for each package of zinc (10 tablets)?	☐☐☐☐ ***UGX***<br>Don't know	 88	→253<br>For all responses
252	Can you tell me why you did not give your child zinc to treat the diarrhea?	Did not know where to buy<br>Zinc is too expensive<br>Used a product I had confidence in<br>Other (specify): _____________	1<br>2<br>3<br>99	
253	***Interviewer: check if q227=3: Home prepared solution was given to the child.***	**Yes**<br>**No**	**1**<br>**2**	 **→Q259**
254	You mentioned that you have given (NAME) a home-prepared solution. Is that correct?<br><br>***Interviewer: If did not give home-prepared solution, correct q226 and q227.***	Yes<br>No<br>Don't know	1<br>2<br>88	 **→Q259**<br>**→Q259**

255	Was (NAME) given a Sugar Salt Solution (SSS) during the episode of diarrhea?	Yes<br>No<br>Don't know	1<br>2<br>88	
256	What was recipe you used for the SSS	RECORD VERBATIM		
257	How often did you give the home-prepared solution to (NAME)?<br><br>***Interviewer: Read the list and ask the respondent to select one response.***	Frequently<br>After each liquid stool<br>Morning, mid-day, and night<br>Whenever the child wanted it<br>Don't know<br>Other (specify): _____________	1<br>2<br>3<br>4<br>88<br>99	
258	Did you use ordinary water or did you use treated water when you prepared the home-based treatment?	Ordinary (Non Purified) Water<br>Treated Water<br>Other (specify): _____________	1<br>2<br>99	
259	***Interviewer: check Q227 for whether EITHER 1 or 3 are selected: ORS/SSS was given to the child.***	**ORS/SSS was given**<br>**No ORS/SSS was given**	**1**<br>**2**	**→Q261**
260	Why did you not give (NAME) any ORS or SSS solutions?<br><br>***Interviewer: Do not read list. Multiple responses allowed.***	Child not seriously ill<br>Could not find anywhere to get ORS<br>Did not know how to prepare SSS<br>Products too costly<br>Child does not like the taste<br>Didn't know about ORS/SSS<br>It is not a real treatment<br>Not very effective treatment<br>Too far to go to retreive<br>Other (specify): _____________	1<br>2<br>3<br>4<br>5<br>6<br>7<br>8<br>9<br>99	
261	***Interviewer: check if q227=4: Antibiotic was given to the child.***	**Yes**<br>**No**	**1**<br>**2**	 **→Q265**
262	You mentioned that you have given (NAME) an antibiotic. Is that correct?<br><br>***Interviewer: If did not give antibiotic, correct q226 and q227.***	Yes<br>No<br>Don't know	1<br>2<br>88	 **→Q265**<br>**→Q265**
263	Where did you obtain this antibiotic?<br><br>***Interviewer: Mark only one answer.***	**<u>Public Sector</u>**<br>Health center<br>Community distributor (VHT or CHP)<br>Other public sector<br><br>**<u>Private Sector</u>**<br>Private Clinic/provider<br>Private pharmacy/drug store<br>Faith-based, NGO/CBO<br>Friends/Relatives<br>Traditional healer<br>Don't know<br>Other (specify): _____________	 1<br>2<br>3<br><br><br>4<br>5<br>6<br>7<br>8<br>88<br>99	
264	Why did you give (NAME) an antibiotic to treat diarrhea?<br><br>***Interviewer: Do not read list. Multiple responses allowed.***	Child had blood in stool<br>Child had fever with diarrhea<br>Health provider said it is more effective<br>I asked for an antibiotic<br>Other (specify): _____________	1<br>2<br>3<br>4<br>99	

265	***Interviewer: check if q227=5: Antidiarrheal was given to the child.***	**Yes**<br>**No**	**1**<br>**2**	 **→Q269**
266	You mentioned that you have given (NAME) an Antidiarrheal. Is that correct?<br><br>***Interviewer: If did not give Antidiarrheal, correct q226 and q227.***	Yes<br>No<br>Don't know	1<br>2<br>88	 **→Q269**<br>**→Q269**
267	Where did you obtain the antidiarrheal?<br><br>***Interviewer: Mark only one answer.***	**Public Sector**<br>Health center<br>Community distributor (VHT or CHP)<br>Other public sector<br><br>**Private Sector**<br>Private Clinic/provider<br>Private pharmacy/drug store<br>Faith-based, NGO/CBO<br>Friends/Relatives<br>Traditional healer<br>Don't know<br>Other (specify): _______________	 1<br>2<br>3<br><br><br>4<br>5<br>6<br>7<br>8<br>88<br>99	
268	Why did you give (NAME) an antidiarrheal to treat diarrhea?<br><br>***Interviewer: Do not read list. Multiple answers allowed***	Health provider said it is more effective<br>I think it is most effective<br>I asked for an antidiarrheal<br>This treatment has worked well for me in the past<br>Only treatment available in shop<br>Other (specify): _____________<br>________________________________<br>________________________________	1<br>2<br>3<br>4<br>5<br>99	
269	***Interviewer: check if q227=6: Intravenous fluid was given to the child.***	**Yes**<br>**No**	**1**<br>**2**	 **→Q272**
270	You mentioned that you gave (NAME) an intravenous fluid treatment. Is that correct?<br><br>***Interviewer: If did not give an Intravenous fluid, correct q226 and q227.***	Yes<br>No<br>Don't know	1<br>2<br>88	 **→Q272**<br>**→Q272**
271	Where did you obtain this intravenous treatment?<br><br>***Interviewer: Mark only one answer.***	**Public Sector**<br>Health center<br>Community health worker<br>Other public sector<br><br>**Private Sector**<br>Private Clinic/provider<br>Private pharmacy/drug store<br>Community distributor<br>Faith-based, NGO/CBO<br>Friends/Relatives<br>Traditional healer<br>Don't know<br>Other (specify): _______________	 1<br>2<br>3<br><br><br>4<br>5<br>6<br>7<br>8<br>9<br>88<br>99	

272	***Interviewer: check if q227=7:<br>Injection was given to the child.***	**Yes**<br>**No**	**1**<br>**2**	 **→Q275**
273	You mentioned that you gave (NAME) an injection.<br>Is that correct?<br><br>***Interviewer: If did not give an injection, correct q223 and q251.***	Yes<br>No<br>Don't know	1<br>2<br>88	 **→Q275**<br>**→Q275**
274	Where did you obtain this injection?<br><br>***Interviewer: Mark only one answer.***	**Public Sector**<br>Health center<br>Community health worker<br>Other public sector<br><br>**Private Sector**<br>Private Clinic/provider<br>Private pharmacy/drug store<br>Community distributor<br>Faith-based, NGO/CBO<br>Friends/Relatives<br>Traditional healer<br>Don't know<br>Other (specify): _____________	 1<br>2<br>3<br><br><br>4<br>5<br>6<br>7<br>8<br>9<br>88<br>99	
275	**Interviewer: Check if Q226=1:<br>Child received treatment for their diarrhea.**	**Yes**<br>**No**	**1**<br>**2**	**→279**
276	Can you tell me why you did not provide any treatment to (NAME) during this recent episode of diarrhea?	Child not very sick<br>Could not afford<br>Did not know where to purchase treatment<br>Child too young for drugs<br>No treatment available in my area<br>Don't know<br>Other (specify): _____________	1<br>2<br>3<br>4<br>5<br>88<br>99	
277	Did (NAME) have **symptoms of malaria** in the last 4 weeks?	Yes<br>No<br>Don't know	1<br>2<br>88	 **→279**
278	Did you give (NAME) any treatment for his/her malaria symptoms?	Yes<br>No<br>Don't know	1<br>2<br>88	

ORS use of other household members

Interviewer: Ask once all children with diarrhea in the past 4 weeks have been inquired about (Q201-Q278)

279	Did anyone else in your household aside from the children we discussed use ORS for any reason in the past 4 weeks?	Yes<br>No<br><br>Don't Know	1<br>2<br>88	 **→282**
280	Was this ORS stored in the household?	Yes<br>No<br>Don't Know	1<br>2<br>88	
281	Who used the ORS?	Child older than 5<br>Sibling<br>Husband<br>Other		

282	Do you have any salt stored in your household?	Yes<br>No<br>Don't Know	1<br>2<br>88	
283	Do you have any sugar stored in your household?	Yes<br>No<br>Don't Know	1<br>2<br>88	

Use of Other Health Products

284	In the past 4 weeks did you use any chlorine tablets to make your water clean and safe for your child/children to drink?	Yes<br>No<br>Don't know	1<br>2<br>88	→286
285	How often did you use chlorine tablets to clean the water you gave to your child?	Rarely<br>Some of the time<br>Most of the time<br>Always	1<br>2<br>3<br>4	
286	In the past 4 weeks did your children sleep under a bed net?	Yes<br>No<br>Don't know	1<br>2<br>88	**→288**
287	How often did your children sleep under a bed net?	Rarely<br>Some of the time<br>Most of the time<br>Always	1<br>2<br>3<br>4	

HOUSEHOLD QUESTIONNAIRE: DIARRHEA MODULE

Contact with Community Health Promoter

"Now I am going to ask you some questions about the community health promoter (CHP) in your village. A CHP is someone in your village that visits households and sells health products and other household goods."

288	Is there a community health promoter in your village?	Yes<br>No<br>Don't know	1<br>2<br>88	 →Q301<br>→Q301
289	How often does the CHP visit your household?	Every Week<br>Every Month<br>Every 3 Months<br>Less Than Every 3 Months<br>Never Visited My Household	1<br>2<br>3<br>4<br>5	 <br><br><br>→Q301
290	What does the CHP do when they visit your household?<br><br>**Do Not Prompt. Circle all that apply.**	Hygiene Training<br>Diarrhea Treatment Training<br>Child Health Training<br>Product Sales<br>Other (Record Verbatim)___________	1<br>2<br>3<br>4<br>5	
291	Does the CHP ever talk to you about how to treat your child's diarrhea?	Yes<br>No<br>Don't know	1<br>2<br>88	
292	Has the CHP Visited Your Household in the **past 4 weeks**?	Yes<br>No<br>Don't know	1<br>2<br>88	 →Q301<br>→Q301
293	What did the CHP do when they visit your household?<br><br>**Do Not Prompt. Circle all that apply.**	Hygiene Training<br>Diarrhea Treatment Training<br>Child Health Training<br>Product Sales<br>Deliver ORS+Zinc<br>Other (Record Verbatim) ___________	1<br>2<br>3<br>4<br>5	
294	Did the CHP talk to you about how to treat your child's diarrhea?	Yes<br>No<br>Don't know	1<br>2<br>88	

SECTION 3 – BELIEFS ABOUT DIARRHEA AND TREATMENT

301	If your child becomes sick with diarrhea, what do you think the best way to treat the child is?<br><br>***Instructions to enumerators: Do not prompt. Record all that apply.***	No treatment<br>Increased Fluids<br>Increased Food<br>Herbal remedies<br>Antibiotics<br>Antidiarrheals<br>Zinc<br>ORS<br>Home-made sugar salt solution<br>Others specify_____<br>Don't know	1<br>2<br>3<br>4<br>5<br>6<br>7<br>8<br>9<br>99<br>88	
Interviewer: **Check if 301=8 (Respondent chose ORS as best way to treat diarrhea). Ask 302 and 303 if 301=8. Otherwise skip to 304.**				
302	How soon after the childs diarrhea symptoms begin should you begin giving the child ORS?<br><br>***Interviewer: Do not read responses. Probe to classify as one of the response options***	Immediately (after 1st loose stool)<br>After child has multiple loose stools<br>After 1 day if diarrhea persists<br>After 2 days if diarrhea persists<br>Other specify____	1<br>2<br>3<br>4<br>99	
303	How frequently should the child be given ORS?<br><br>***Interviewer: Do not read responses. Probe to classify as one of the response options***	Once per day<br>Twice per day<br>Three times per day<br>Four times per day<br>After each loose stool<br>Other specify____	1<br>2<br>3<br>4<br>5<br>99	
Interviewer: **Check if 301=9 (Respondent chose Zinc as best way to treat diarrhea). Ask 304-306 if 301=9. Otherwise skip to 307.**				
304	How soon after the childs diarrhea symptoms begin should you begin giving the child Zinc?<br><br>***Interviewer: Do not read responses. Probe to classify as one of the response options***	Immediately (after 1st loose stool)<br>After child has multiple loose stools<br>After 1 day if diarrhea persists<br>After 2 days if diarrhea persists<br>Other specify____	1<br>2<br>3<br>4<br>99	
305	How frequently should the child be given Zinc?<br><br>***Interviewer: Do not read responses. Probe to classify as one of the response options***	Only one time per episode<br>Every other day<br>Once per day<br>Twice per day<br>Three times per day<br>Four times per day<br>After each loose stool<br>Other specify____	1<br>2<br>3<br>4<br>5<br>6<br>7<br>99	
306	For how many days should the child continue to receive Zinc.<br><br>***Interviewer: Record number of days. Record 99 if respondents reports "until 10 tablets used" or "until packet it empty".***	[ ][ ] ***days***<br><br>Until 10 tablets used	 <br>99	

*This section asks your opinion on certain issues. Please tell me if you **believe that** the following statements **are true or false.***

**1. Ability: Knowledge**				
		**True**	**False**	**Don't know**
307	Diarrhea can be caused by lack of cleanliness	1	0	88
308	Diarrhea can be associated with lack of cleanliness, such as not washing hands with water and soap before eating	1	0	88
309	Diarrhea can be caused by drinking unsafe water	1	0	88
310	Diarrhea can be caused by eating unclean food	1	0	88
311	Antibiotics should only be used for certain kinds of diarrhea	1	0	88
312	Most diarrhea can be managed at home without any treatment	1	0	88
313	Giving food-based fluids is equally as effective as giving ORS	1	0	88
314	Diarrhea can be caused by growing teeth	1	0	88

*Please tell me if you **"agree strongly," "agree somewhat," "disagree strongly,"** or **"disagree somewhat"** with the following statements.*

**2. Motivation: Threat Severity**					
		**Strongly Agree**	**Agree Somewhat**	**Disagree Somewhat**	**Strongly Disagree**
315	Children can die from diarrhea	4	3	2	1
316	Your family will have a problem if one of the members has diarrhea	4	3	2	1
317	It does not seem like anyone around here has a problem because of diarrhea	4	3	2	1
318	Diarrhea is a major health problem in your community	4	3	2	1
319	Diarrhea is a problem in the poorer segment of the community only	4	3	2	1

**3. Motivation: Threat Susceptibility (Children Under Five)**					
		**Strongly Agree**	**Agree Somewhat**	**Disagree Somewhat**	**Strongly Disagree**
320	If your child gets diarrhea it is best just to do nothing and it will pass in time	4	3	2	1
321	The children under five in your household are healthy so their bodies can fight off diarrhea without doing anything	4	3	2	1
322	Children under five are too young to experience serious medical problems from getting diarrhea	4	3	2	1
323	You are not worried about the children (child) under five in your household getting diarrhea	4	3	2	1
324	Children are more likely to get diarrhea than adults	4	3	2	1

HOUSEHOLD QUESTIONNAIRE: DIARRHEA MODULE

Interviewer: Skip the following questions if respondent has not heard about ORS in Q120.

**4. Opportunity: Availability**						
		**Strongly Agree**	**Agree Somewhat**	**Disagree Somewhat**	**Strongly Disagree**	**Don't know**
325	Drug stores nearby always have ORS for sale	4	3	2	1	88
326	ORS treatments are difficult to get around here	4	3	2	1	88
327	There is a place nearby where you can get ORS when your child needs it	4	3	2	1	88
328	You don't know where to get ORS	4	3	2	1	88
329	ORS treatments are too expensive	4	3	2	1	88
330	You are willing to pay the current price for ORS (UGX 400-500 per sachet)	4	3	2	1	88
331	ORS treatment products are available within walking distance from your home	4	3	2	1	88
**5. Motivation: Outcome Expectations**						
		**Strongly Agree**	**Agree Somewhat**	**Disagree Somewhat**	**Strongly Disagree**	**Don't know**
332	ORS is effective for treatment of diarrhea	4	3	2	1	88
333	ORS reduces the duration of a diarrheal episode	4	3	2	1	88
334	ORS does not help in reducing the severity of a diarrheal episode	4	3	2	1	88
335	Use of ORS reduces the risk of dehydration in children	4	3	2	1	88
336	ORS reduces the risk of a new diarrheal episode in the following 2 to 3 months	4	3	2	1	88
337	ORS helps to strengthen the immune system of children	4	3	2	1	88
**6. Capacity/Ability: Use of Products**						
		**Strongly Agree**	**Agree Somewhat**	**Disagree Somewhat**	**Strongly Disagree**	**Don't know**
338	ORS should be used for every type of child diarrhea	4	3	2	1	88
339	All child diarrhea should be treated with an antibiotic	4	3	2	1	88
340	ORS has too many side effects, so you don't feel safe giving ORS to your small child	4	3	2	1	88
341	ORS tastes bad so your child won't take it.	4	3	2	1	88
342	You would use ORS the next time your child has diarrhea if you had to pay a small fee for it.	4	3	2	1	88
343	You would use ORS the next time your child has diarrhea if it were free.	4	3	2	1	88

Interviewer: Skip the following questions if respondent has not heard about zinc in Q126.

**4. Opportunity: Availability**						
		**Strongly Agree**	**Agree Somewhat**	**Disagree Somewhat**	**Strongly Disagree**	**Don't know**
344	Drug stores nearby always have zinc for sale	4	3	2	1	88
345	Zinc treatments are difficult to get around here	4	3	2	1	88
346	There is a place nearby where you can get zinc when your child needs it	4	3	2	1	88
347	You don't know where to get zinc	4	3	2	1	88
348	Zinc treatments are too expensive	4	3	2	1	88
349	You are willing to pay the current price for zinc (UGX 1000 per 10 tablets)	4	3	2	1	88
350	Zinc treatment products are available within walking distance from your home	4	3	2	1	88
**5. Motivation: Outcome Expectations**						
		**Strongly Agree**	**Agree Somewhat**	**Disagree Somewhat**	**Strongly Disagree**	**Don't know**
351	Zinc is effective for treatment of diarrhea	4	3	2	1	88
352	The child should stop receiving Zinc once the diarrhea stops	4	3	2	1	88
353	Zinc reduces the duration of a diarrheal episode	4	3	2	1	88
354	Zinc does not help in reducing the severity of a diarrheal episode	4	3	2	1	88
355	Use of zinc reduces the risk of dehydration in children	4	3	2	1	88
356	Zinc reduces the risk of a new diarrheal episode in the following 2 to 3 months	4	3	2	1	88
357	Zinc helps with the ability of my child to stay healthy	4	3	2	1	88
**6. Capacity/Ability: Use of Products**						
		**Strongly Agree**	**Agree Somewhat**	**Disagree Somewhat**	**Strongly Disagree**	**Don't know**
358	Zinc should be used for every type of child diarrhea	4	3	2	1	88
359	Child diarrhea should be treated with an antibiotic	4	3	2	1	88
360	Zinc has too many side effects, so you don't feel safe giving zinc to your small child	4	3	2	1	88
361	Zinc tastes bad so your child won't take it.	4	3	2	1	88
362	Zinc is only a nutritional supplement, not an effective treatment for pediatric diarrhea.	4	3	2	1	88
363	Zinc should be given along with	4	3	2	1	88

	ORS to be most effective.					
364	It is difficult to remember to give a child zinc when the diarrhea has stopped	4	3	2	1	88
365	You would use zinc the next time your child has diarrhea if you had to purchase it	4	3	2	1	88
366	You would use zinc the next time your child has diarrhea if it were free	4	3	2	1	88

END OF DIARRHEA MODULE

FORM C.

Checking Packaging and Incentive Payment

1. **Did the community health promoter in your village provide you with any ORS and zinc packets about 4 weeks ago?**

   Yes………..1
   No…………2 →[End Interview]

2. **Do you still have any of the packaging, used or unused, from the ORS and zinc you were provided?**

   Yes………..1
   No…………2 →[End Interview]

3. **Can I please see the packaging you still have?**

   Yes………..1
   No…………2 →[End Interview]

4. **Interviewer: record observation of packets**

   a. **Total number of ORS packets (full and empty)____________**

   b. **Total number of empty ORS packets___________**

   c. **Total number of full ORS packets___________**

   d. **Total number of zinc packets (full and empty)____________**

   e. **Total number of zinc tablets used___________**

   f. **Total number of zinc tablets remaining___________**

Interviewer: provide respondents who had at least one ORS or zinc packet with incentive payment

THANK YOU FOR PARTICIPATING IN THIS STUDY!

END TIME /___/___/___/___/

INTERVIEWER: PLEASE MAKE SURE HOUSEHOLD UNIQUE ID IS INDICATED ON TOP OF THE IDENTIFICATION TABLE
